# Effect of Antibiotics on Gut Microbiota, Gut Hormones and Glucose Metabolism

**DOI:** 10.1371/journal.pone.0142352

**Published:** 2015-11-12

**Authors:** Kristian H. Mikkelsen, Morten Frost, Martin I. Bahl, Tine R. Licht, Ulrich S. Jensen, Jacob Rosenberg, Oluf Pedersen, Torben Hansen, Jens F. Rehfeld, Jens J. Holst, Tina Vilsbøll, Filip K. Knop

**Affiliations:** 1 Center for Diabetes Research, Gentofte Hospital, University of Copenhagen, Hellerup, Denmark; 2 NNF Centre for Basic Metabolic Research and Department of Biomedical Sciences, Faculty of Health and Medical Sciences, University of Copenhagen, Copenhagen, Denmark; 3 Endocrine Research Unit, University of Southern Denmark, Odense, Denmark; 4 National Food Institute, Technical University of Denmark, Søborg, Denmark; 5 Department of Clinical Microbiology, Rigshospitalet, University of Copenhagen, Copenhagen, Denmark; 6 Department of Surgery, Herlev Hospital, University of Copenhagen, Herlev, Denmark; 7 Department of Clinical Biochemistry, Rigshospitalet, University of Copenhagen, Copenhagen, Denmark; Vanderbilt University, UNITED STATES

## Abstract

**Objective:**

The gut microbiota has been designated as an active regulator of glucose metabolism and metabolic phenotype in a number of animal and human observational studies. We evaluated the effect of removing as many bacteria as possible by antibiotics on postprandial physiology in healthy humans.

**Methods:**

Meal tests with measurements of postprandial glucose tolerance and postprandial release of insulin and gut hormones were performed before, immediately after and 6 weeks after a 4-day, broad-spectrum, per oral antibiotic cocktail (vancomycin 500 mg, gentamycin 40 mg and meropenem 500 mg once-daily) in a group of 12 lean and glucose tolerant males. Faecal samples were collected for culture-based assessment of changes in gut microbiota composition.

**Results:**

Acute and dramatic reductions in the abundance of a representative set of gut bacteria was seen immediately following the antibiotic course, but no changes in postprandial glucose tolerance, insulin secretion or plasma lipid concentrations were found. Apart from an acute and reversible increase in peptide YY secretion, no changes were observed in postprandial gut hormone release.

**Conclusion:**

As evaluated by selective cultivation of gut bacteria, a broad-spectrum 4-day antibiotics course with vancomycin, gentamycin and meropenem induced shifts in gut microbiota composition that had no clinically relevant short or long-term effects on metabolic variables in healthy glucose-tolerant males.

**Trial Registration:**

clinicaltrials.gov NCT01633762

## Introduction

The human gut is populated by a dense community of microbes (the gut microbiota) that many-fold outnumbers our eukaryotic cell count and provides the host with an enormous complimentary microbial gene set (the gut microbiome). Several metabolic disease states such as obesity and type 2 diabetes have been linked with alterations in the microbiota composition and function [1–3], and in animal models, it has been demonstrated that the microbiota actively contributes to a number of host metabolic pathways such as energy harvesting potential, regulation of gut hormone secretion and nutrient storage [4–6].

Antibiotics cause marked short-term disturbances in the human gut microbiota, and incomplete recovery of the microbiota to its initial composition has been found in some patients following antibiotic treatment [7–10]. In observational studies, exposure to antibiotics has been linked with development of obesity and type 2 diabetes [11–13]. Moreover, a 7-day course of vancomycin, but not ampicillin, was followed by a reduction in peripheral insulin sensitivity in a randomized controlled trial [14]. Increased body mass index (BMI) and altered levels of appetite regulating gut hormones were found following antibiotic eradication of *Helicobacter pylori* in a cohort of patients referred to upper endoscopy [15]. Decreased insulin sensitivity following a one-week antibiotic course with vancomycin was reported in obese males with metabolic syndrome, whereas no change in insulin sensitivity was observed following administration of amoxicillin for one week [16]. Thus, antibiotics seem to possess the potential to affect host metabolism by altering the gut microbiota configuration, while the physiological importance and potential clinical implications of antibiotic-induced microbiota alterations remain to be demonstrated.

We designed a prospective clinical study in order to elucidate how a short-term broad-spectrum antibiotics course targeting intestinal bacteria affects postprandial physiology in healthy glucose tolerant male adults.

## Methods

### Study approval and registration

The study was approved by the Ethical Committee in the Capital Region of Denmark (H3-2011-144, S1 File and S1 Protocol) and registered on clinicaltrials.gov (NCT01633762), although the subject enrolment began 4 months before registration on clinicaltrials.gov (unawareness). The study was conducted according to the principles of the Helsinki Declaration II and written informed consent was obtained from all study participants.

### Study design

In addition to a screening visit the study design encompassed 5 study visits (day 0, 4, 8, 42 and 180) and a 4-day 3-drug antibiotic course (see below) between the first 2 study visits (day 0 and 4) composed to eradicate as many gut bacteria as possible (S1 Fig). At all 5 study visits, bodyweight, height and blood pressure were measured, health questionnaires were completed and fasting blood samples and a faecal sample were collected. In addition, on 3 of the study days (day 0, 4 and 42) a standardised meal test with repeated blood sampling was performed. Except from keeping the diet unaltered throughout the study period, and avoiding yoghurt products on the 4 days preceding each visit, no dietary regulations were required.

### Participants

Twelve healthy male volunteers were recruited (between March and September 2012) using the following inclusion criteria: age 18–40 years, Caucasian ethnicity, informed written consent, glycated haemoglobin A1c (HbA_1c_) <43 mmol/mol (<6.1%), normal bowel function (bowel movements 1-3/day) and fasting plasma glucose (FPG) <6.0 mmol/l. Exclusion criteria included any use of antibiotics 6 months prior to inclusion, BMI <18.5 kg/m^2^ or >25 kg/m^2^, smoking, abnormal serum/plasma levels of electrolytes, lipids, creatinine, liver enzymes (alanine transaminase, aspartate aminotransferase, alkaline phosphatase), thyroid stimulating hormone or haemoglobin, any current or existing disease in the gastrointestinal system or family history of inflammatory bowel disease or diabetes, lactose intolerance or coeliac disease, allergy against the used antibiotics or use of medication that could not be paused during the study period. Subjects had a mean age of 23.4 years (standard deviation 5.3 years) at the start of the study.

### Study visits

Study visits were conducted between April 2012 (first participant, first visit) and April 2013 (last participant, last visit). At all 5 visits, study participants arrived in the laboratory in the morning after an overnight fast, having avoided strenuous physical exercise and alcohol consumption for the last 24 h. Participants’ height and weight were measured after voiding.

On study visits days 0, 4 and 42 standardised liquid meal tests were performed: Participants were sitting in a semi recumbent position in a hospital bed, blood pressure was measured (Microlife BP A100, Microlife, Widnau, Switzerland), an intravenous catheter was inserted in a cubital vein for collection of blood samples and the catheterised forearm was wrapped in a heating pad (50°C) throughout the experiment in order to arterialise the venous blood. After baseline sampling, participants ingested, within 5 minutes, a 2,205 kJ-liquid mixed meal (Nutridrink, Nutricia, Allerød, Denmark) with 64.4 g carbohydrate, 20.3 g fat, 21.0 g protein, and, for evaluation of gastric emptying, 1.5 g paracetamol. Blood samples were drawn 30, 15, and 0 minutes before and 15, 30, 45, 60, 75, 90, 120, 150, 180, 210 and 240 minutes after ingestion of the mixed liquid meal. For bedside measurement of plasma glucose concentrations, blood was added to fluoride tubes and centrifuged immediately at 7,400 *g* for 2 minutes at room temperature. For measurements of gut hormone concentrations in plasma, blood was collected in chilled tubes containing ethylenediaminetetraacetic acid (EDTA) and a specific dipeptidyl peptidase 4 (DPP-4) inhibitor (valine-pyrrolidide, a gift from Novo Nordisk A/S, Bagsværd, Denmark; final concentration in sample: 0.03 mmol/l). For the analyses of insulin and C-peptide blood was sampled in dry tubes, which were left to coagulate for 20 minutes at room temperature before centrifugation. For the analyses of C-reactive protein (CRP) and lipids, blood was collected in lithium-heparin tubes. All samples were centrifuged for 20 minutes at 1,200 *g* and 4°C except for one EDTA tube for the analysis of HbA_1c_. Plasma and serum from day 0, 4 and 42, respectively, were stored at -20°C until analysis, whereas plasma from day 8 and 180, for practical reasons, was stored at -80°C. Resting metabolic rate was measured from time 210 to 225 min after meal intake by indirect calorimetry using a tight facemask connected to a calorimeter, which measures the gas exchange breath-by-breath via an O_2_ alkali cell and an infrared CO_2_ sensor (CCMexpress®, Medgraphics, St. Paul, Minnesota, USA). Metabolic rates are represented as averages of measures carried out every 10 second over the 15 minute-period. Gallbladder dimensions were determined by ultrasound examination before, 25, 55, 90 and 235 minutes after meal intake using a 3.5 MHz-transducer (Logiq 9, GE Healthcare, Little Chalfont, UK). Appetite ratings (hunger, fullness, desire to eat, nausea, bloating and thirst) were assessed using validated visual analogue scales (VASs) [17,18] at baseline and with 30 minute-intervals during the meal test as previously described [18]. At time point 270 min, participants were served a standardised solid *ad libitum* meal consisting of minced meat, pasta, corn, carrots, and green pepper (5.9 g fat/100 g, 5.6 g protein/100 g, and 17.2 g carbohydrate/100 g, corresponding to 6.1 kcal/g). The participants were instructed to eat until they felt pleasantly satiated and intake was measured as the difference between the weight of the served meal and the remaining food. This test has been validated to reproduce spontaneous food intake [19].

On study visits day 8 and 180, blood pressure was measured in the sitting position (Microlife BP A100, Microlife, Widnau, Switzerland) and a blood sample was taken from a cubital vein.

### Antibiotics treatment

At the end of the day 0 meal test, participants ingested an antibiotic ‘cocktail’ containing 500 mg meropenem (powder for infusion; Farmaplus, Oslo, Norway), 500 mg vancomycin (powder for infusion; Fresenius Kabi, Bad Homburg, Germany) and 40 mg gentamicin (solution for injection; Sandoz, Basel, Switzerland) dissolved in 150 ml of apple juice. The ‘cocktail’ was a modified version of previous protocols used for prophylactic treatment of intensive care unit patients [20,21]. It was designed to eradicate as many gut bacteria as possible with the lowest possible risk of side effects. None of the 3 types of antibiotics are absorbed by the healthy mucosa, and therefore have no direct effects on metabolism [22–24]. On day 1, 2 and 3, participants ingested the same antibiotic ‘cocktail’ at the same time of the day.

### Collection of faecal samples

The day before each of the 5 study visits, the participants collected a faecal sample into a vial (Sarstedt feces tube, 76 × 20 mm, Sarstedt, Nümbrecht, Germany), which was placed in a transport container (Sarstedt, Nümbrecht, Germany) and stored in the participant’s own -20°C freezer overnight. During the transportation to the hospital, the sample was kept frozen in a thermo bag with 2 ice packs, and upon arrival in the laboratory, the sample was immediately transferred to a -80°C freezer.

### Evaluation of diet, gastrointestinal function and health-related symptoms

At each of the 5 study days, participants completed a standardised questionnaire about their intake of common food items, number of bowel movements per day and stool consistency (Bristol Stool Scale, with a value above 4 defined as loose stool [25]) on each of the 4 preceding days. Diarrhoea was defined according to World Health Organization (≥3 loose stools per day [26]). Participants were given a personal dairy and were asked to note any health-related symptom, use of medication or change in activity or eating habits throughout the study period.

### Laboratory methods

Plasma glucose was measured by the glucose oxidase method, using a glucose analyzer (Yellow Springs Instrument model 2300 STAT plus analyzer; YSI, Yellow Springs, Ohio, USA). Serum insulin and C-peptide concentrations were measured using a 2-sided direct chemiluminescence immunoassay (ADVIA Centaur XP; Siemens, Erlangen, Germany). Plasma CRP was measured using an immunochemical assay, with a high-sensitive test used for CRP levels below 9.00 mg/l (Vitros 5.1 FS; Ortho Clinical Diagnostics, Johnson & Johnson Medical, Birkerød, Denmark). Plasma lipids (total cholesterol, high-density lipoprotein (HDL), and triglyceride) and paracetamol were quantified using an enzymatic colorimetric assay (Vitros 5.1 FS; Ortho Clinical Diagnostics, Johnson & Johnson Medical, Birkerød, Denmark). By use of Friedewald’s formula very-low-density lipoprotein (VLDL) was calculated from the amount of triglycerides multiplied by 0.45 and low-density lipoprotein (LDL) from the amount of total cholesterol minus the amount of HDL and VLDL. HbA_1c_ was measured by high Performance Liquid Chromatography (Variant II TURBO, Bio-Rad, Hercules, California, USA). Plasma concentrations of cholecystokinin (CCK), glucose-dependent insulinotropic polypeptide (GIP), glucagon-like peptide-1 (GLP-1) and gastrin were measured by specific radioimmunoassays, as previously described [27–29]. Peptide YY (PYY)3-36 (the major circulating molecular form of PYY generated by DPP-4 mediated degradation of PYY1-36) was measured by a commercial radioimmunoassay (cat#PYY-67HK, Milipore, Billerica, Massachusetts, USA) according to the manufacturer’s recommendations. All quality controls were within acceptable limits.

Enumeration of bacteria in faecal samples was performed by the plate counting method. From partially thawed faecal samples 0.1 g was transferred to 0.9 ml maximum recovery diluent and a 10-fold dilutions series was prepared and plated on 5 different agar media for enumeration of colony forming units (CFU). Plate count agar, McConkey agar No. 3 and Slanetz and Bartley agar (Oxoid, Roskilde, Denmark) were used for enumeration of total aerobic bacteria (and facultative anaerobes), Coliform bacteria and Enterococci respectively following aerobic incubation at 37°C for 1, 2 or 3 days. Wilkins-Chalgren agar (Oxoid) and BSM agar (Sigma-Aldrich, Brøndby, Denmark) were used for enumeration of total anaerobic bacteria and bifidobacteria following incubation in an anaerobic chamber for 2 or 3 days. The choice of the 3 specific bacterial groups was based on their taxonomic diversity (belonging to 3 different phyla) and their natural presence in the human gut microbiota.

Concentration of vancomycin and gentamicin in faecal samples was determined by chemiluminescent immunoassay (Architect c4000, Abbott, Chicago, Illinois, USA) [30], while faecal concentrations of meropenem were measured indirectly using an agar-cup assay [31].

### Calculations and statistical analysis

Statistical analyses were carried out using SAS 9.3 (SAS Institute, Cary, North Carolina, USA). Results are reported as means and 95% confidence intervals (CI) unless otherwise stated. For comparison of differences between baseline value and day 4, 8, 42 and 180 values, the PROC MIXED procedure was applied with participant and day as class variables, participant as random effect and day 0 value (baseline) as reference. Faecal bacterial counts, lipid measurements and all tAUC values were log10-transformed in order to improve the fit to the model. Test for interaction between day and time was performed in order to see if the relationship between time and postprandial excursions of glucose, pancreatic hormones and gut hormones were different on day 0, 4 and 42. If no interaction was detected, postprandial excursions of glucose, insulin and gut hormones were summarised into area under the curve (AUC) values, calculated using the trapezoidal rule and presented as total AUC (tAUC) values.

Gallbladder volume (V) was expressed in cm^3^ and calculated using the ellipsoid formula: V = 1/6 *π*abc, where a = maximum length, b = maximum width, and c = maximum depth, as described previously [32]. Matsuda index of insulin sensitivity, insulinogenic index and oral disposition index were calculated as described previously [33–35]. Homeostasis model assessments of insulin resistance (HOMA2-IR) was calculated using the HOMA Calculator version 2013 (http://www.dtu.ox.ac.uk, accessed July 2014). An indirect measure of gastric emptying rate was obtained by calculating time-to-peak of plasma acetaminophen concentration.

## Results

### Participants

**Fig 1:**
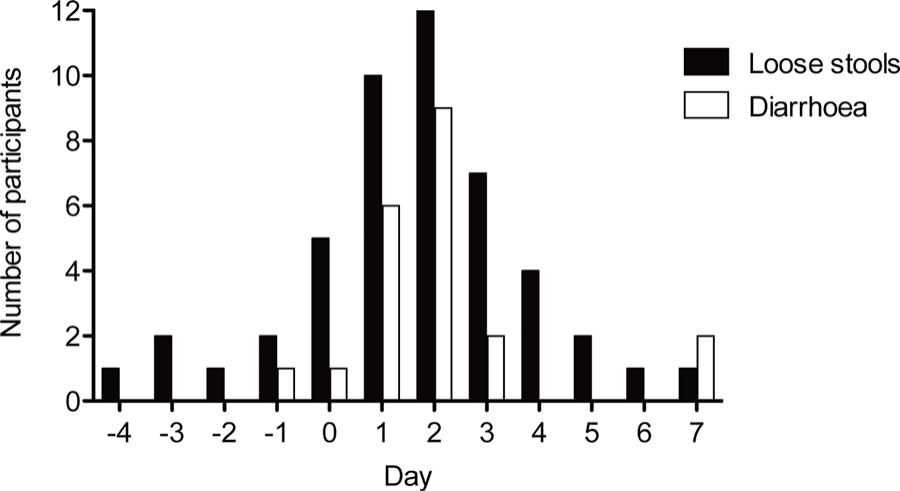
Number of participants reporting loose stools and diarrhoea in the days before, during and immediately after the antibiotics course. Loose stools are defined as stool consistency on the Bristol Stool Scale chart of >4. Diarrhoea was defined as 3 or more loose stools per day. All participants had loose stools after having received the antibiotic course for 3 days.

Antibiotic treatment did not result in any serious or unexpected adverse events. During the treatment period, loose stools were frequent and 9 of 12 participants had diarrhoea on 1 or more of the 4 treatment days as shown in Fig 1. None of the 12 participants reported diarrhoea on day 4 when the second meal test was conducted, and gastrointestinal symptoms (mild abdominal pain and loose stools for a few days) were only reported by 1 participant during day 8 to day 180 (day 25–27).

No changes in bodyweight were observed between day 0 and day 4, 8 or 42, but a small increase in mean bodyweight from 78.1 (73.9–82.4) to 79.4 (78.2–80.6) kg was seen from day 0 to day 180 (p = 0.04) with a corresponding increase in mean BMI from 22.6 (21.3–23.8) to 22.9 (21.3–23.8) kg/m^2^ (p = 0.04) (Table 1). There were no changes in mean blood pressure. No health complaints or symptoms were reported during the study period, apart from a case of epididymitis (treated with per oral doxycycline for 3 days on day 26–28 of the study), a common cold and the above-mentioned gastrointestinal symptoms.

**Table 1:** 

	Day 0	95% CI	Day 4	95% CI	Day 8	95% CI	Day 42	95% CI	Day 180	95% CI
**Bodyweight (kg)**	78.1	(73.9–82.4)	78.2	(76.4–78.7)	77.6	(76.4–78.7)	77.7	(76.5–78.9)	79.4†	(78.2–80.6)
**BMI (kg/m** ^**2**^ **)**	22.6	(21.3–23.8)	22.6	(22.1–22.7)	22.4	(22.1–22.7)	22.4	(22.1–22.8)	22.9†	(22.6–23.3)
**Glucose (mmol/l)**	5.09	(4.91–5.28)	5.11	(4.99–5.24)	-		5.21	(5.09–5.34)		
**HbA**_**1c**_ **(mmol/mol)**	32	(31–33)	-		-		31	(30–32)	32	(31–33)
**HbA**_**1c**_ **(%)**	5.08	(4.96–5.20)					5.00	(4.88–5.11)	5.06	(4.94–5.17)
**Cholesterol (mmol/l)**	3.6	(3.3–4.1)	3.6	(3.4–3.9)	3.9†	(3.7–4.2)	3.6	(3.4–3.9)	3.9†	(3.7–4.2)
**LDL (mmol/l)**	1.9	(1.6–2.3)	1.9	(1.8–2.2)	2.2†	(2.0–2.5)	1.9	(1.7–2.1)	2.2†	(2.0–2.4)
**HDL (mmol/l)**	1.1	(1.0–1.2)	1.1	(1.0–1.2)	1.2	(1.1–1.3)	1.1	(1.0–1.1)	1.1	(1.0–1.2)
**VLDL (mmol/l)**	0.6	(0.4–0.8)	0.5	(0.4–0.7)	0.5	(0.4–0.6)	0.5	(0.4–0.7)	0.5	(0.4–0.7)
**Triglyceride (mmol/l)**	1.2	(0.9–1.6)	1.2	(0.9–1.5)	1.0	(0.8–1.3)	1.2	(0.9–1.5)	1.1	(0.9–1.5)
**CRP (mg/l)**	0.4	(0.2–0.7)	0.3	(0.1–0.7)	0.4	(0.2–0.8)	0.6	(0.3–1.4)	0.5	(0.2–1.1)

Bodyweight, body mass index (BMI), blood concentration of glycated haemoglobin (HbA_1c_) and plasma concentration of glucose, cholesterol, low-density lipoprotein (LDL), high-density lipoprotein (HDL), very-low-density lipoprotein (VLDL), triglyceride and C-reactive protein (CRP) measured in the fasting state on each of the 5 study days. Data are expressed as mean (geographical mean for log-transformed data) and 95% confidence intervals (CI) in brackets.

**†** denotes p<0.05, indicating a significant change compared to day 0.

### Blood samples taken in the fasting state

No change from baseline in FPG or HbA_1c_ was seen and no changes in plasma concentrations of total cholesterol, LDL, HDL, VLDL or triglyceride were seen on the meal test days (day 0, 4 and 42). The basal concentrations of total cholesterol and LDL were slightly higher on day 8 and 180 compared to day 0 (p<0.05), with no significant changes in triglyceride concentrations (Table 1). The plasma concentration of CRP was below 3 mg/l in 59 of the 60 samples, and was unchanged by the antibiotic course (Table 1).

### Cultivation of faecal samples

**Fig 2:**
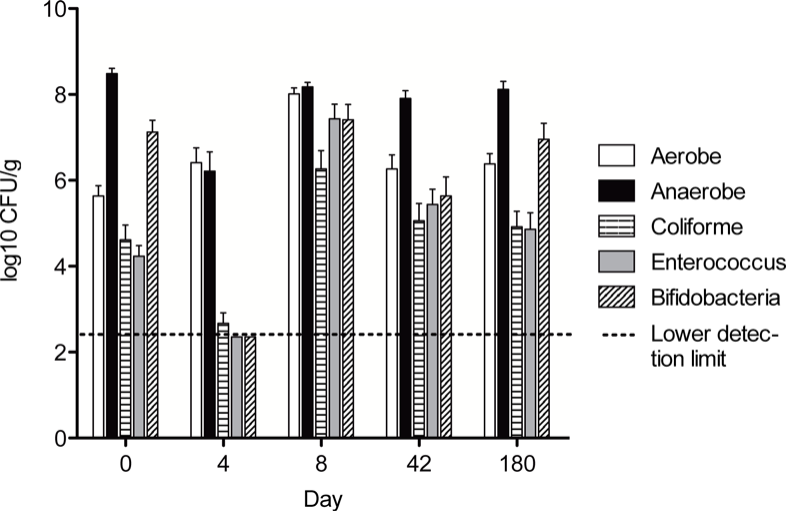
Abundance of faecal bacteria expressed as means (±standard error of the mean) of log_10_-transformed number of colony forming units (CFU) per gram of faeces upon cultivation. The dotted line shows the lower detection limit. Reductions were observed in the abundance of faecal anaerobes, coliform, enterococci and bifidobacteria from day 0 to day 4. At 180 days after the antibiotics course, both the total aerobic and total anaerobic bacterial counts as well as the 3 specific bacterial groups had returned to the same levels as prior to the antibiotics course.

As shown in Fig 2, the total anaerobic bacterial count decreased from 8.5 (7.9–9.0) log_10_ CFU/g before antibiotics to 6.2 (5.5–6.9) log_10_ CFU/g immediately following the antibiotic course (p<0.0001). Enterococci, coliforms and bifidobacteria also decreased markedly following the antibiotic course (falling from 4.2, 4.6 and 7.1 log_10_ CFU/g, respectively, on day 0, to below the detection limit on day 4 (p<0.01 for all groups)). On day 8, the abundance of aerobic (and facultative anaerobic) bacteria, enterococci and coliform bacteria was significantly above the pre-antibiotic level indicating increased growth and colonisation of these groups in the period immediately after the antibiotic course. 180 days after the antibiotic course, both the total bacterial counts and the 3 specific bacterial groups had returned to the same levels as prior to the antibiotic treatment.

Antibiotics were measurable in day 4 faecal samples from all participants indicating compliance to the antibiotic prescription.

### Postprandial plasma glucose excursions and serum insulin responses

**Fig 3:**
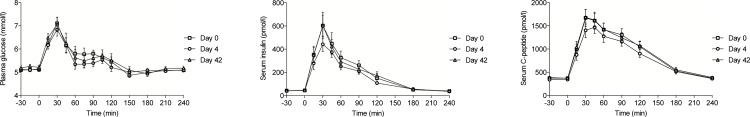
Mean (±standard error of the mean) postprandial excursions of plasma glucose, insulin and C-peptide on day 0, 4 and 42. No significant change in postprandial plasma glucose, insulin or C-peptide responses (assessed as area under curve) was observed.

No change in fasting serum insulin or C-peptide concentration, tAUC_glucose_, tAUC_insulin_, tAUC_C-peptide_, HOMA, Matsuda, insulinogenic or disposition indices were observed between baseline and day 4 or 42 (Table 2 and Fig 3). However, there was a tendency (p = 0.05) towards a slightly reduced tAUC_insulin_ on day 4.

**Table 2:** 

	Day 0	Day 4	95% CI	Day 42	95% CI
**Glucose**	1	0.98	(0.95–1.01)	1.00	(0.97–1.04)
**Insulin**	1	0.85	(0.73–1.00)	1.06	(0.90–1.24)
**C-peptide**	1	0.90	(0.81–1.00)	1.03	(0.93–1.15
**Gastrin**	1	1.07	(0.94–1.22)	0.95	(0.84–1.09)
**CCK**	1	0.97	(0.86–1.10)	0.97	(0.86–1.10)
**GIP**	1	1.06	(0.92–1.22)	1.09	(0.94–1.25)
**GLP-1**	1	1.05	(0.75–1.48)	1.20	(0.85–1.68)
**PYY**	1	1.40†	(1.20–1.63)	1.08	(0.92–1.25)

Relative changes in postprandial plasma/serum excursions of glucose, pancreatic and gut hormones on day 4 and 42 expressed as mean change in total area under curve (tAUC) with day 0 as reference (with 95% confidence intervals (CI) in brackets), e.g. tAUC_PYY_ increased 40% from day 0 to day 4.

**†** denotes p<0.05. CCK, cholecystokinin, GIP, glucose-dependent insulinotropic polypeptide, GLP-1, glucagon-like peptide-1, PYY, peptide YY.

### Postprandial plasma gut hormone responses

**Fig 4:**
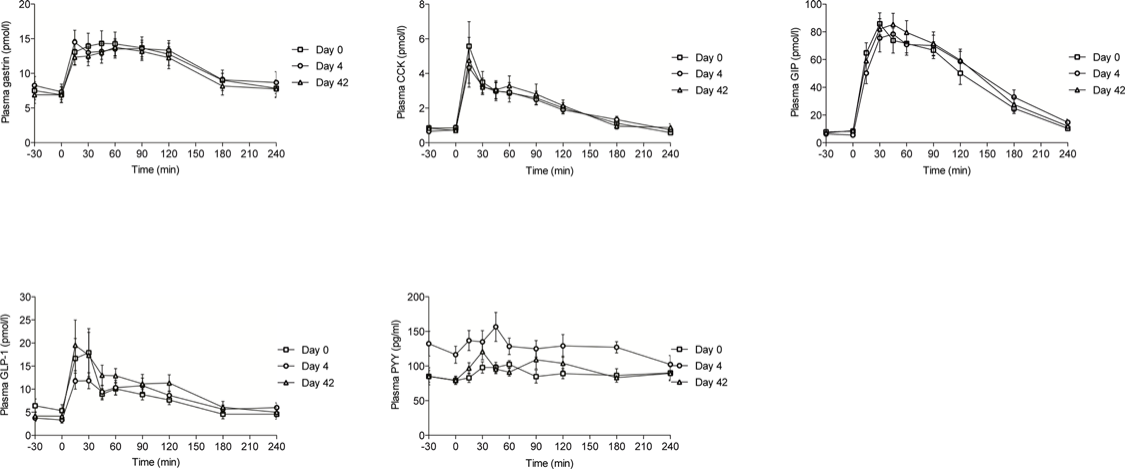
Mean (±standard error of the mean) postprandial excursions of plasma gastrin, cholecystokinin (CCK), glucose-dependent insulinotropic polypeptide (GIP), glucagon-like-peptide 1 (GLP-1) and peptide YY (PYY) on day 0, 4 and 42. An acute reversible increase in plasma PYY concentration was seen following the antibiotics course, whereas no significant changes were seen in the plasma concentrations of the other gut hormones.

No change in basal or postprandial (tAUC) concentrations of the gut hormones gastrin, CCK, GIP or GLP-1 was observed from day 0 to 4 or 42 (Table 2). In contrast, tAUC_PYY_ increased by 40% from day 0 to day 4, whereas no change in tAUC_PYY_ was found from baseline to day 42 (Table 2 and Fig 4).

### Gastric emptying, gallbladder emptying, appetite, food intake and basal metabolic rate

No change in resting metabolic rate, composite appetite score or *ad libitum* food intake was found between day 0, 4 and 42 (S1 Table). No significant change in gall bladder volume at any of the 5 time points (-15, 25, 55, 90 and 235 min) on day 0, 4 and 42 were found and no significant changes in time-to-peak plasma paracetamol concentration were seen (S1 Table).

## Discussion

We report that a short-term broad-spectrum antibiotic course in healthy young males results in 1) acute and dramatic reductions in the abundance of a representative set of gut bacteria confirmed by cultivation of faecal samples, 2) acutely and reversibly increased circulating PYY3-36 concentrations, but 3) no changes in glucose tolerance or release of gastrin, CCK, GIP and GLP-1.

Although substantial changes in metabolic phenotype has been previously seen in animals exposed to antibiotics [36–38], few human interventional studies have investigated the effect of antibiotic treatment on gut hormone secretion or glucose metabolism. In a Dutch study, 20 obese patients with metabolic syndrome were randomised to receive 7 days of per oral treatment with either vancomycin (500 mg thrice-daily) or amoxicillin (500 mg thrice-daily) [16]. Mixed meal tests performed before and immediately after the treatment period showed no changes in postprandial glucose tolerance or secretion of the gut-derived incretin hormones GIP and GLP-1, but hyperinsulinaemic euglycaemic clamps showed a slight decrease in peripheral insulin sensitivity in the group receiving vancomycin compared to no change in the amoxicillin group. In contrast, a recent follow-up to this study showed no difference in insulin sensitivity following vancomycin or amoxillin compared to placebo treatment in obese males with impaired glucose tolerance [39]. In line with the latter, we found no change in tAUC_insulin_, tAUC_C-peptide_ or HOMA, Matsuda or insulinogenic indices between study days in the present study with a 4 day-duration of antibiotics intervention.

The gut microbiota has been suggested to affect host glucose metabolism by at least 4 different mechanisms: Through modulation of the bile acid pool [16,40], stimulation of the innate immune system [41], or by fermentation of complex carbohydrates to short-chain fatty acids that can either function directly as metabolic substrates or influence the secretion of gut hormones [42]. Regarding the first 2 mechanisms, we did not find any change in gallbladder volume or emptying, and we saw no changes in CRP. In relation to the latter mechanism it was recently reported that germ-free rodents with low intestinal levels of short-chain fatty acids had higher expression of proglucagon, higher GLP-1 levels and higher density of enteroendocrine L cells, the cell responsible for secretion of GLP-1 as well as PYY [43]. The high GLP-1 secretion was abolished upon bacterial colonisation of the germ free animals, but could be re-established by subjecting conventionally raised rodents to a short course of broad-spectrum antibiotics expected to eradicate most of the gut bacteria [43]. Although PYY has well-established effects on appetite sensation and food intake, a direct effect of PYY on glucose metabolism remains controversial [44]. Increased secretion of PYY has previously been reported in patients with diarrhoea following gastrointestinal infection [45] and an important function of PYY may be to limit nutrient and water excretion by reducing colonic transit time and fluid secretion [46,47]. In our study, several participants reported diarrhoea during the antibiotic course, and after completion of the course, plasma PYY concentrations were increased. It might therefore be hypothesised that the increased PYY secretion is a physiologic response to limit water and nutrient loss following (antibiotic-induced) diarrhoea. We saw a decrease in faecal abundance of short-chain fatty acid producing genera (bifidobacteria) [48] at the same time that plasma concentrations of PYY went up, raising the question of the role of short-chain fatty acids for this response. However, the general belief is that short-chain fatty acids stimulate L cell secretion [49] and perhaps it is rather increased exposure of the L cells with loose stool fluid containing other L cell stimulants (unabsorbed nutrients or bile acids), that is responsible for the increased PYY secretion.

Several limitations of the current study should be addressed. Firstly, as we did not include an untreated comparison group, we cannot rule out random variation or study effects as explanations for the negative results or the observed differences between baseline and subsequent study days. The finding of a small increase in bodyweight on day 180 is in line with observational studies reporting long-term increased risk of obesity following antibiotics [11,12,15,50], but could also reflect aging of the participants or seasonal variation [51]. The findings of increased plasma cholesterol concentrations on day 8 and 180, with no changes on day 4 and 42 are not supported by previous studies, do not seem biologically meaningful and may result from natural variation and/or slight deviations in study procedures on day 8 and 180 compared to day 0, 4 and 42 (see methods). Secondly, the low participant number means a low statistical power for detecting small changes. However, the confidence intervals of the observed variables (Table 1 and Table 2) were generally narrow, indicating that we did not ignore a clinically relevant metabolic difference. Thirdly, for ethical reasons, considering the risk of antibiotic resistance development and the lack of knowledge about metabolic effects of antibiotics at the time of the study initiation, we chose to employ a short-term antibiotics course. Although this antibiotics course indeed led to dramatic acute changes in microbiota load and composition, we cannot exclude the possibility that other antibiotic courses could induce even more dramatic effects on the gut microbiota, and subsequently could have more pronounced or sustained metabolic effects. This possibility seems particularly plausible if antibiotics are given multiple times, in higher dosages or during critical periods of life such as early life [12,38,50]. Lastly, although we found no long-term changes in the abundance of several common gut bacterial groups, it is important to emphasize that compositional changes at the phylum, genus or species level will not be detected by the applied cultivation technique, nor did our investigation assess if metabolically relevant changes occurred in the gut microbiome.

Interestingly, we observed an increase in faecal abundance of several bacterial groups sub-acutely following antibiotics, which is in line with a recent human study using qPCR to assess changes in gut microbiota following antibiotic treatment [9]. A likely explanation is that the increased bacterial groups have occupied new ecological niches in the gut, which were made available by the antibiotic treatment.

## Conclusion and Future Directions

An acute reversible increase in the postprandial plasma level of the gut hormone PYY, but no clinically relevant changes in insulin sensitivity, insulin release or release of other gut hormones were seen following 4-day broad-spectrum antibiotics course in healthy young males. There was an apparent lack of association between substantial shifts in gut microbiota as evaluated by cultivation and overall unchanged metabolic variables, contrasting the current dogma, which implies the gut microbiota to be a key player in metabolic regulation. However, sustained or pronounced metabolic effects of antibiotics given in other dosages or to other populations cannot be excluded based on this study. In a recent population-based case-control study, exposure to narrow-spectrum antibiotics was more strongly associated with development of type 2 diabetes, than exposure to general or broad-spectrum antibiotics [13]. Data on the long-term metabolic effect of commonly used narrow-spectrum antibiotics (such as phenoxymethylpenicillin) therefore seems warranted.

## Supporting Information

S1 TREND Checklist TREND Checklist. (PDF)

S1 Fig Study design. The study encompassed 5 study visits (arrows) and a 4-day 3-drug antibiotic course. At all 5 study visits, bodyweight, height and blood pressure were measured, health questionnaires were completed and fasting blood samples and a faecal sample were collected. In addition, on 3 of the study days (day 0, 4 and 42) a standardised meal test with repeated blood sampling was performed.
(TIFF)

S1 File Study protocol approved by ethical committee, in Danish. (DOCX)

S1 Protocol Study protocol approved by ethical committee, in English. (DOCX)

S1 Table Resting metabolic rate, gastric emptying, fasting and postprandial gallbladder volumes, composite appetite score and food intake during ad libitum meal. No significant changes were observed in any of the above variables from before to immediately after or 42 days after the antibiotics course. Data shown as mean values (with 95% confidence intervals (CI)).
(DOCX)
