# Supplementary figures and images for: Effect of Antibiotics on Gut Microbiota, Gut Hormones and Glucose Metabolism
Source: PLoS One. 2015 Nov 12;10(11):e0142352. doi: 10.1371/journal.pone.0142352 (PMC4643023; doi:10.1371/journal.pone.0142352)

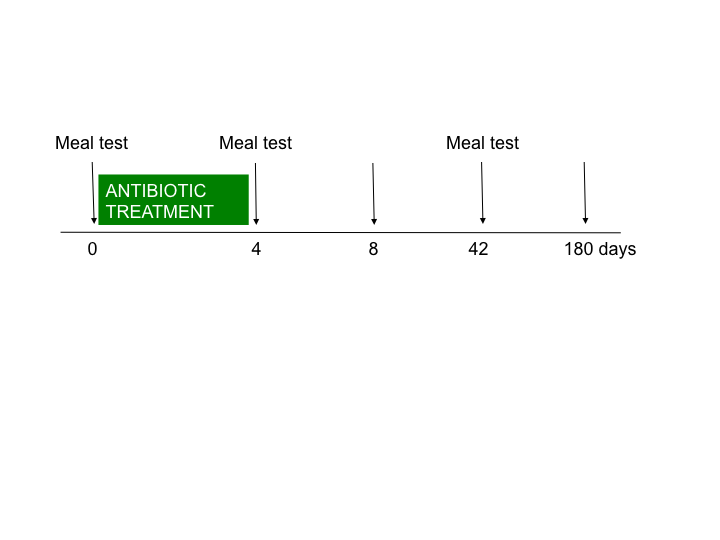

Supplement: S1 Fig — The study encompassed 5 study visits (arrows) and a 4-day 3-drug antibiotic course. At all 5 study visits, bodyweight, height and blood pressure were measured, health questionnaires were completed and fasting blood samples and a faecal sample were collected. In addition, on 3 of the study days (day 0, 4 and 42) a standardised meal test with repeated blood sampling was performed. (TIFF) [file pone.0142352.s002.tiff]
