# Supplementary material for: Effect of Antibiotics on Gut Microbiota, Gut Hormones and Glucose Metabolism
Source: PLoS One. 2015 Nov 12;10(11):e0142352. doi: 10.1371/journal.pone.0142352 (PMC4643023; doi:10.1371/journal.pone.0142352)
Supplement: S1 File — (DOCX) [file pone.0142352.s003.docx]

**Eradikation af den humane tarmflora**

*Effekt på postprandial tarmhormonsekretion, glukosemetabolisme, knogleomsætning og tarmmikrobiom*

Materiale til den Videnskabsetiske Komité for Region Hovedstaden

Version 3

*Et samarbejde mellem*

Diabetologisk Forskningsenhed, Gentofte Hospital, Københavns Universitet

Læge Kristian Hallundbæk Mikkelsen

læge, ph.d. Morten Frost Nielsen

Overlæge, dr. med. Tina Vilsbøll

1. reservelæge, ph.d. Filip Krag Knop

Klinisk mikrobiologisk afdeling, Rigshospitalet, Københavns Universitet

Ledende overlæge Michael Tvede

Novo Nordisk Foundation Center for Basic Metabolic Research, Københavns Universitet

Professor, ph.d. Torben Hansen

Professor, dr. med. Oluf Borbye Pedersen

Gastroenheden, Kirurgisk sektion, Herlev Hospital, Københavns Universitet

Professor, dr. med. Jacob Rosenberg

Biomedicinsk Institut, Panum, Københavns Universitet

Professor, dr. med. Jens Juul Holst

**OVERSIGT OVER DET INDSENDTE MATERIALE**

FORSØGSPROTOKOL

BILAG 1, LÆGMANDSRESUMÉ

BILAG 2, DELTAGERINFORMATION

BILAG 3, SAMTYKKEERKLÆRING, DELTAGELSE I FORSKNINGSPROJEKT

BILAG 4, SAMTYKKEERKLÆRING, GENNEMFØRELSE AF KIKKERTUNDERSØGELSER

BILAG 5, ANNONCETEKST PÅ WWW.FORSOEGSPERSON.DK

BILAG 6, KOSTREGISTRERINGSSKEMA

BILAG 7, FORSØGSPERSONERS RETTIGHEDER I ET BIOMEDICINSK FORSKNINGSPROJEKT

BILAG 8, FØR DU BESLUTTER DIG.......................................................................................

# FORSØGSPROTOKOL

## INTRODUKTION

Type 2-diabetes mellitus (T2DM) og osteoporose er hyppigt forekommende sygdomme forårsaget af blandt andet genetisk disposition samt uhensigtsmæssig livsstil^1,2,3,4^. Nyere studier tyder på, at tarmens bakterieflora kan have betydning ved disse sygdomme. Således er ændringer i tarmbakteriekvantitet og -kvalitet associeret med forekomsten af T2DM og osteoporose. Indtil nu har intet studie undersøgt effekten af at modulere bakteriefloraen på glukose- og knoglemetabolismen hos mennesker.

## FORMÅL

Vi ønsker at afdække, om eradikation af tarmbakteriefloraen hos raske unge mænd indvirker på sekretionen af tarmhormoner med betydning for en række fysiologiske mekanismer (herunder inkretinhormoners indvirkning på appetit, mæthed, den endokrine pancreasfunktion og glukosemetabolismen), knogleomsætning samt inflammationsmarkører. Specifikt ønsker vi at undersøge effekten af at eradikere den normalt forekommende tarmbakterieflora ved hjælp af 4-dags bredspektret antibiotikabehandling hos raske unge mænd. Forhåbentlig vil resultaterne fra nærværende undersøgelse bidrage til en forståelse af den nyopdagede sammenhæng mellem tarmbakterieflora og henholdsvis metaboliske sygdomme og osteoporose.

## BAGGRUND

T2DM og osteoporose er hyppigt forekommende sygdomme med multifaktorielle ætiologier herunder genetisk disposition og livsstil^1,2,3,4^. Til trods for sygdommenes kliniske forskelligheder lader det til, at knogle- og sukkeromsætning indvirker på hinanden^5^, og for nyligt er man blevet opmærksom på, at den intestinale tarmflora kan spille en væsentlig rolle i forhold til begge disse sygdomme.

Den humane tarmbakterieflora bidrager til vigtige metaboliske funktioner, herunder evnen til at optage næringsstoffer fra ellers ufordøjelige fødeemner^6^. Ud fra afføringsprøver fra tvillinger er det foreslået, at der findes en kernefunktionalitet af tarmens bakterieflora, men at hvert individ har sin egen helt unikke sammensætning af bakteriearter^7^. Modsat har *Arumugam et al*. foreslået, at den humane tarmflora hos det enkelte individ, på tværs af kontinenter, er stratificeret til 1 af 3 ”enterotyper” med hver deres specialiserede metabolisme og artssammensætning^8^. Endnu vides det ikke, hvordan disse enterotyper korrelerer med sygdomsforekomst eller metabolisme. For nyligt er det vist, at enterotyperne korrelerer til kostens indhold af henholdsvis kulhydrater og fedt/protein^9^, og et dansk studie har vist, at den intestinale bakteriesammensætning i kvalitet og kvantitet er korreleret til forekomsten af T2DM samt plasmaglukosekoncentrationer i en gruppe af voksne mennesker^10^.

Præliminære data fra *Vrieze et al*.^11^har vist, at transplantation af fæces fra slanke individer til overvægtige med metabolisk syndrom forbedrede sidstnævntes insulinfølsomhed sammenlignet med autolog fæcestransplantation i samme gruppe. Yderligere fandt *Gordon et al*.^12^, at gnavere, opvokset under kimfri forhold, udviklede nedsat kropsfedt trods øget energiindtag sammenlignet med gnavere opvokset under normale betingelser. Transplantation af fæces fra de normalt opfostrede til de sterilt opfostrede mus var endvidere forbundet med en vægtstigning samt en øget insulinresistens hos sidstnævnte. Eradikation af den intestinale bakterieflora hos overvægtige, insulinresistente gnavere med perorale antibiotika er tilsvarende vist at kunne forbedre disse dyrs fasteblodglukose samt resultatet af oral glukosetolerancetest (OGTT)^13^.

Kostens sammensætning synes at påvirke den intestinale bakteriesammensætning hos gnavere uafhængigt af deres vægt/sygdomsstatus: Efter en fedtrig diæt har man fundet en tilsvarende ændring i tarmflorasammensætning mellem henholdsvis genmodificerede ’vægtstigningsresistente’ mus og *wildtype*-mus på trods af, at der kun sås vægtstigning hos sidstnævnte gruppe^14^.

Hvorledes den intestinale bakterieflora medierer ovenstående effekter er ikke afklaret, men på følgende fysiologiske variabler har man fundet effekter af en moduleret tarmbakterieflora^15^:

- Koncentration og sekretion af lokalt- og systemisk virkende tarmpeptider og -hormoner
- Koncentration og sekretion af galdesyrer
- Produktion af kortkædede fedtsyrer i tarmlumen
- Produktion af serumlipopolysakkarider
- Produktion af en række cytokiner

*Glucose-dependent insulinotropic polypeptide* (GIP) og *glucagon-like peptide-1* (GLP-1) er tarmhormoner, som potenserer glukosestimuleret insulinsekretion og er ansvarlige for ca. 70% af insulinsekretionen efter peroral indtagelse af glukose^16^. Således kan enhver faktor, der nedsætter GIP- og GLP-1-sekretionen, øge det postprandiale plasmaglukoseniveau. Det er tidligere vist, at rotter fodret med præbiotika (ikke-fordøjelige men fermentarbare fødemidler som stimulerer vækst og/eller aktivitet af en eller flere tarmmikrober med gavnlig virkning på den humane vært^17^) udvikler højere postprandiale GIP- og GLP-1-responser^18^. Man kunne derfor forestille sig, at en modificeret tarmflora ville indvirke på glukosemetabolismen.

Gnavere opvokset under kimfri forhold er vist at have en 3 gange forøget galdesyrekoncentration i galden samt en 25 % forøget kolesterolabsorption sammenlignet med naturligt opfostrede dyr^19^. Samtidig er en 3-dags ampicillinkur vist at kunne øge galdesyresekretionen med en faktor 3 samt nedsætte fækalt galdesyretab med 70 % hos mus^20^. Eftersom galdesyrer for nylig er vist at påvirke glukosemetabolismen (via påvirkning af den G-proteinkoblede receptor TGR5 på GLP-1-secernerende L-celler i tarmslimhinden)^21^ indikerer ovenstående fund et hidtil uerkendt samspil mellem bakterieflora, galdesyresekretion og glukosemetabolisme.

I forhold til osteoporose er der over de senere år også fundet interessante koblinger til den intestinale flora samt udskillelsen af tarmhormoner. I et nyligt studie fandt man, at ændringer i tarmfloraen hos mus påvirker essentielle knoglekvaliteter som densitet og struktur^22^. Forfatterne fandt en betydeligt forøget knogletæthed korreleret til et lavere serumserotoninniveau hos mus, der voksede op under sterile vs. almindelige forhold. Imidlertid var det ikke muligt ud fra forsøget at afklare, om dette fund var et resultat af øget knogledannelse eller nedsat knogleresorption.

*Abrams et al*. fandt i et humant studie fra 2005, at tilskud med præbiotika vs. kontroldiæt medførte en signifikant forøget knoglemasse og -tæthed samt et øget kalciumoptag i en gruppe af teenagere^23^. Gnaverstudier har bekræftet dette fund^17^.

I lyset af de tidligere beskrevne sammenhænge mellem tarmhormoner og præbiotika er det desuden interessant, at samme tarmhormoner er fundet associerede med knogleomsætning. Hos mus har man påvist en nedsat knogleformation og en øget osteoklastkoncentration hos GIP-receptor-*knockout*-mus sammenlignet med *wildtype*-mus^24^. Tilsvarende fandt man kortikal osteopeni, øget osteoklastaktivitet og øget knogleresorption hos GLP-1-receptor-*knockout*-mus sammenlignet med *wildtype*-mus^25^.

Perorale antibiotikakure er tidligere anvendt til profylaktisk at eradikere intestinale bakterier forud for kolonkirurgi^26^, hos intensivpatienter^27^, cirrosepatienter med hepatisk encefalopati^28^ og neutropene patienter med hæmatologiske sygdomme^29^. Mens effekten af disse kure er dokumenteret med hensyn til infektionsrisiko og mortalitet, er effekten på knogle- og sukkeromsætning indtil videre ikke belyst. I et nyligt studie af patienter eradikeret for *helicobacter pylori* med amoxicillin og clarithromycin fandt man, at de eradikerede patienter syv måneder efter eradikationen havde et signifikant højere *bodymass index* (BMI) samt signifikant højere postprandial plasmaleptin- og -ghrelinresponser end før eradikation^30^.

Således har et stigende antal studier fundet, at den intestinale bakterieflora har stor betydning for glukose- og knoglemetabolismen. Det står dog ikke klart, hvordan de involverede effekter er medieret. Endvidere synes eradikation af tarmfloraen at kunne have modsatrettede virkninger på glukosemetabolismen - muligvis afhængig af hvilken tarmflorasammensætning, der forudgående eksisterer i individet.

I en tid med stigende forbrug af antibiotika^31^ og stigende forekomst af T2DM^32^ og osteoporose^33^ synes det relevant at undersøge, om der er en sammenhæng mellem de ovenfor gennemgåede fænomener og ændringer i tarmbakteriefloraen, hvilket er sigtet med nærværende studie.

## ENDEPUNKTER

I denne undersøgelse vil vi undersøge, om eradikation af tarmbakterierne hos mennesker medfører forandringer i glukose- og knoglemetabolismen. Vi vil desuden undersøge sammensætningen af bakterier i fæces, blod og spyt såvel før som efter intervention (eradikation - se nedenfor), vurderet ved både dyrkning af bakterier og *deep metagenomic next-generation sequencing* af bakterie-DNA. Ved hjælp af bioinformatiske analyser undersøges, hvilke funktionelle implikationer ændringerne i bakteriesammensætning på species- og phylumniveau har lokalt i tarm/blod/mundhule og på helkropsniveau*.* Endelig vil vi belyse effekten af tarmbakterieeradikation på genaktiviteten i tarmslimhinde samt spyt og urin foruden sammensætningen af inflammationsmarkører før og efter interventionen. Hos deltagere, der - udover at samtykke til deltagelse i hovedprotokollen - samtykker til at få foretaget gastroduodenoskopi vil effekten af interventionen på sammensætningen af bakterier i duodenalsekret og genekspression i duodenalslimhinden blive vurderet. Undersøgelsens endepunkter i relation til de specifikke formål er anført nedenfor:

- Effekten af tarmbakterieeradikation på sekretionen af tarm- og pancreashormoner undersøges ved hjælp af postprandiale plasmakoncentrationsmålinger af inkretinhormonerne GIP og GLP-1, CCK, gastrin, ghrelin, PYY, GLP-2, oxyntomodulin, insulin, C-peptid og glukagon før og efter 4-dags eradikationskur (se nedenfor)
- Effekten af tarmbakterieeradikation på metaboliske variable i plasma og urin vurderes ved hjælp af *metabolomics*-analyse af henholdsvis plasma fra fasteblodprøver og urin indsamlet før og efter 4-dags eradikationskur (se nedenfor)
- Effekten af tarmbakterieeradikation på hvilestofskifte vurderes ved hjælp af indirekte kalorimetri foretaget før og efter 4 dags eradikationskur (se nedenfor)
- Effekten af tarmbakterieeradikation på kropsvægt, appetit, mæthed og fødeindtag vil blive vurderet ved hjælp af vægtregistrering, standardiserede spørgeskemaer (vedrørende appetit og mæthed før, under og efter måltidstest (visuel analogskala (VAS)) samt kostregistrering) og registrering af indtagen fødemængde i forbindelse med et ad libitum-måltid før og efter 4-dags eradikationskur (se nedenfor)
- Effekten af tarmbakterieeradikation på knogleomsætning undersøges ved hjælp af fasteplasmakoncentrationsmålinger af markører for knogleformation (osteocalcin, P1NP) og -resorption (CTX, 1CTP, sklerostin) samt serotonin før og efter 4-dags eradikationskur (se nedenfor)
- Effekten af tarmbakterieeradikation på bakteriesammensætningen i fæces, spyt og blod vurderes ved såvel bakteriedyrkninger som *deep metagenomic next-generation sequencing* af fæces, spyt og blod (opsamlet før og efter interventionen beskrevet nedenfor); hos deltagere, der samtykker til gastroduodenoskopi, vil effekten af interventionen på bakteriesammensætningen i duodenalsekret blive vurderet på tilsvarende vis
- Effekten af tarmbakterieeradikation på systemisk inflammation vurderes ved måling af følgende serum-/plasmamarkører for inflammation (fasteblodprøver): *high sensitive* CRP, *lipopolysaccharide binding protein* (LPBP), fibrinogen, TNF-, IL-6, PAI-1, leptin og adiponectin
- Effekten af tarmbakterieeradikation på sekretionen af galde før og efter tarmbakterieeradikation vurderes ved hjælp af ultralydsundersøgelse af den postprandiale galdeblæretøming samt måling af koncentrationen af de forskellige galdesyrer i blodet før og efter 4-dags eradikationskur (se nedenfor)
- Effekten af tarmbakterieeradikation på genekspression i duodenalslimhinde vurderes ved eksplorative og hypotesegenererende genekspressionsanalyser (analyse af kandidatgener samt *microarray*-analyser) udført på duodenale mucosabiopsier udtaget under standard gastroduodenoskopi før og efter interventionen beskrevet nedenfor

## METODER OG DESIGN

### Forsøgsdeltagere

12 raske, unge mænd rekrutteres ved hjælp af opslag på Gentofte Hospital og annoncering (bl.a. på www.forsoegsperson.dk).

#### Inklusionskriterier

- Mænd, alder 18-40 år
- Dansk kaukasisk etnicitet
- Evne til at afgive informeret samtykke
- Normal HbA1_c_ (<6 %) og/eller normal glukosetolerance vurderet ved 75 g-OGTT
- Normal fasteplasmaglukose (<6 mM)
- Fastende serumlipider indenfor normalområde
- Normal thyroideafunktion
- Spiser gængs og varieret dansk kost
- Er ikke-ryger
- Normale afføringsvaner, dvs. 1-3 gange i døgnet

#### Eksklusionskriterier

- Diabetes, forhøjet fasteplasmaglukose og/eller nedsat glukosetolerance
- Kendt knoglelidelse
- Leversygdom (ALAT eller ASAT >2 gange over øvre normalværdi)
- Nyreinsufficiens (serumkreatinin>130 μM)
- Anæmi
- BMI <18.5 kg/m^2^ eller BMI >25 kg/m^2^
- Kendt gastrointestinal sygdom (inklusiv tidligere fedmekirurgi, laktoseintolerans, cøliaki, inflammatorisk tarmsygdom) eller kendt familiær disposition til inflammatorisk tarmsygdom, laktoseintolerans eller cøliaki.
- Antibiotisk behandling inden for 6 måneder før forsøget (inklusiv malariaprofylakse)
- Enhver medicinsk behandling, som ikke kan pauseres under forsøget
- Kontraindikationer mod/allergi over for de anvendte antibiotika (inkl. tidligere allergisk reaktion i forbindelsen med indgift af beta-lactamantibiotika, aminoglykosider eller vancomycin)
- Kontraindikationer mod/allergi over for det anvendte sedativum, der anvendes ved gastroduodenoskopierne (propofol) samt allergi overfor soja, æg eller jordnødder (krydsallergi med propofol); dette eksklusionskriterium gælder kun deltagere, der har ytret ønske om at få foretaget gastroduodenoskopi

### Eksperimentelt design

Undersøgelsen strækker sig over 6 måneder og indebærer for den enkelte deltager i alt 6 fremmøder (8 fremmøder for deltagere, der samtykker til gastroduodenoskopi) samt en 4-dages antibiotikakur. Personer, som efter skriftlig og mundtlig information accepterer deltagelse i undersøgelsen og bekræfter dette skriftligt, gennemgår følgende program (vist i figur 1):

<6 uger inden forsøget: screeningsbesøg

Dag 0 Møder fastende til standardiseret bakteriefri måltidstest forudgået af aflevering af afførings-, blod-, urin- og spytprøve (afføringsprøve opsamlet i hjemmet under standardiserede omstændigheder forud for undersøgelsen)

Dag 0-3 Tarmbakterieeradikation: Umiddelbart efter måltidstesten startes 4-dags antibiotikakur (se nedenfor); herefter fører deltagerne et almindeligt liv inkl. vanlig kost i perioden (forsøgsdeltagerne er i denne periode i telefonisk kontakt med forsøgsansvarlige læge mindst 1 gang dagligt)

Dag 4: Møder fastende til standardiseret bakteriefri måltidstest forudgået af aflevering af afførings-, blod, urin- og spytprøve (afføringsprøve opsamlet i hjemmet umiddelbart før undersøgelsen)

Dag 8: Møder fastende til blodprøver samt aflevering af afførings-, blod- urin- og spytprøve (afføringsprøve opsamlet i hjemmet umiddelbart før fremmødet)

Dag 42: Møder fastende til standardiseret bakteriefri måltidstest forudgået af aflevering af afførings-, blod-, urin- og spytprøve (afføringsprøve opsamlet i hjemmet umiddelbart før undersøgelsen)

Dag 180: Møder fastende til blodprøver samt aflevering af afførings-, blod, urin- og spytprøve (afføringsprøve opsamlet i hjemmet umiddelbart før fremmødet)

Hos deltagere, der - udover at samtykke til deltagelse i hovedprotokollen - samtykker til at få foretaget gastroduodenoskopier (med opsamling af biopsier og duodenalsekret), vil disse blive udført dels på en selvstændig dag før interventionsstart (fra efter inklusion frem til dag -1) og på dag 3 (umiddelbart før sidste antibiotikadosering). Ved begge fremmøder skal deltagerne være fastende.

### Screening

Deltagerne møder i laboratoriet efter 10 timers faste. Her måles og vejes deltagerne, medicin og anamnese noteres, der måles blodtryk samt tages screeningsblodprøver (kreatinin, elektrolytter (Na^+^ og K^+^), TSH, ALAT, ASAT, basisk fosfatase, hæmoglobin, HbA_1_c og fasteplasmaglukose samt fastende lipidprofil). Der udtages til screeningsblodprøverne ca. 10 ml blod. Såfremt deltagerne på basis af resultaterne fra screeningsmødet kan deltage i projektet, aftales datoer for undersøgelsesdagene. Hvis de biokemiske undersøgelser er abnorme, vil deltagerne blive rådgivet med hensyn til videre udredning og undersøgelser såfremt dette ønskes.

Ved samtlige af de følgende 5 besøg gennemgår forsøgsdeltagerne en kort lægelig vurdering, hvor puls, blodtryk og vægt registreres.

### Måltidstest (dag 0, dag 4 og dag 42)

Forsøgspersonen møder i laboratoriet klokken 8 efter 10 timers faste/tørste (inkl. kaffe, the og evt. medicin). Der anlægges en perifer venekanyler (til blodprøvetagning)i vene på håndryggen, som holdes varm (ca. 50 °C) - for at arterialisere veneblodet - ved hjælp af varmelampe igennem hele forsøget. Inden forsøgsstart tømmes urinblæren og 10 ml tages fra (fordeles i to rør og indfryses ved -20 °C indtil senere analyse). Forsøgspersonen skal herefter og igennem hele forsøget være siddende. 30 minutter før indtagelse af måltidet (tid -30 min) påbegyndes indirekte kalorimetrisk måling af den respiratoriske gasudveksling til *steady-state* over 30 minutter ved hjælp af kalorimeter (CCM Express, Indirect Calorimeter, Medgraphics). Til tiden 0 min påbegyndes indtagelse af et flydende steriliseret måltid over 10 minutter (tid 0-10 min) bestående af 200 ml Nutridrink med kakaosmag (Nutricia, Allerød, Danmark) (300 kcal: 55 g kulhydrat, 17 g fedt og 18 g protein) tilsat 1,5 g paracetamol (brusetabletter) opløst i 50 ml sterilt vand til et samlet volumen på 250 ml (under sterile forhold tages 1 ml-prøve fra til kontroldyrkning og -sekventering for at sikre måltidets sterilitet). Der tages blodprøver fra den perifere venekanyle til tiderne -30, -15, 0, 15, 30, 45, 60, 75, 90, 105, 120, 150, 180, 210 og 240 min. Ved hver blodprøve udtages 0,2 ml blod i natriumflouridrør til bestemmelse af plasmaglukose, 12 ml blod i EDTA-glas til bestemmelse af plasmakoncentrationer af GLP-1, GIP, CCK, gastrin, ghrelin, PYY, GLP-2, oxyntomodulin og glukagon, og 2 ml i tørglas til bestemmelse af serumkoncentrationer af insulin og C-peptid samt paracetamol (til estimering af ventrikeltømningshastighed). Til tiden 0 min udtages herudover ca. 40 ml blod til bestemmelse af knogle- og inflammationsmarkører samt glas til RNA-bestemmelse. Blod/plasma/serum opbevares ved -20/-80 °C indtil analyse. I alt tages ikke over 300 ml blod. Galdeblærevolumen måles ved ultralydsscanning til tiderne 0, 30 og 60 min. Til tiderne 0, 30, 60, 90, 120, 150, 180, 210 og 240 min vurderes personernes appetit (*hunger*), mæthed (*satiety*), fylde (*fullness*) og prospektivt fødeindtag (*prospective food consumption*) ved hjælp af standardiserede VAS’er. Til tiden 60 min foretages igen kalorimetrisk måling over 30 minutter. Til tiden 240 min opsamles total urin til analyse for indhold af glukose og nitrogen og10 ml tages fra (fordeles i to rør og indfryses ved -20 °C indtil senere analyse). Herefter indtages et standardiseret ad libitum-måltid bestående af hakket oksekød, pasta, majs, gulerødder, peberfrugter og fløde samt salt og peber (50 energi (E)% kulhydrat, 37 E% fedt, 13 E% protein). Forsøgspersonerne instrueres i at spise så meget, som de kan, indtil de føler sig behageligt mætte. Måltidet indtages i løbet af maksimalt 30 minutter. Ved afslutning af ad libitum-måltidet noteres tidspunktet samt vægten og energimængde af det indtagne ad libitum-måltid. Forsøgsdeltagerne vurderer endvidere måltidets smag, lugt, visuel appel, eftersmag og samlet velsmag (*palatability*), når de er færdige med måltidet, ved hjælp af standardiserede VAS’er. Umiddelbart efter ad libitum-måltidet på dag 0 indtages første dosis af eradikationskuren (se nedenfor). Efter ad libitum-måltidet på dag 42 tages blodprøve til måling af hæmoglobin og på baggrund af resultatet af denne prøve institueres evt. almindelig peroral jernbehandling i 14 dage og der måles ny hæmoglobin efter yderligere 14 dage.

### Registrering af kostsammensætning og -mængde (dag 0, dag 4, dag 8 og dag 42)

På et tidspunkt under hver måltidstest på dag 0, 4 og 42 (samt ved fremmøde på dag 8) udfyldes valideret *food frequency*-spørgeskema vedrørende deltagernes kostsammensætning og -mængde i de 4 forudgående dage (skemaet er vedlagt som bilag 6).

### Antibiotikakur (dag 0- dag 3)

Efter den første måltidstest opstartes eradikation af den intestinale tarmbakterieflora med 4-dages varende 3-stofs antibiotikakur bestående af 500 mg vancomycin (pulver til infusionsvæske), 500 mg meropenem (pulver til infusionsvæske) og 40 mg gentamicin (pulver til infusionsvæske) opløst i 1 glas (200ml) frugtjuice. Opløsningen gives per oralt 1 gang dagligt i 4 på hinanden følgende dage. Første dosis indtages umiddelbart efter måltidstesten på dag 0, mens forsøgspersonen stadig er på hospitalet. Anden, tredje og fjerde dosis udleveres (i alt 9 hætteglas) til forsøgspersonerne på dag 0 og skal indtages opløst i 200 ml juice på hver af de følgende 3 dage ved aftenstid. Deltagerne opfordres til ikke at ændre deres normale fødeindtag i løbet af undersøgelsen. Forsøgsansvarlig læge kontakter forsøgsdeltagerne pr telefon 1 gang dagligt på dag 1, 2 og 3 med henblik på at sikre korrekt medicinindtag samt udelukke tegn på medicinbivirkninger.

### Fæcesprøver (dag 0, dag 4, dag 8, dag 42 og dag 180)

Afføringsprøverne anvendes til bestemmelse af bakterieantal og -subtyper ved hjælp af to metoder: sekventering af bakterie-DNA samt dyrkning af tarmbakterier. Deltagerne får udleveret opsamlingssæt bestående af køletaske med køleelementer, emballage samt afføringsglas. Afføringsprøverne opsamles i forsøgspersonernes hjem så tæt på leveringstidspunktet som muligt. Den eksakte behandling af afføringsprøverne er forskellig for de to bestemmelsesmetoder.

#### Fæcesopsamling til DNA-sekventering

Forud for defækation placeres en engangsopsamler i toilettet hvorfra afføring overføres til to glas som straks overføres til en transportcontainer og anbringes ved -20 °C i egen fryser. Forud for transport til Gentofte Hospital overføres transportcontaineren til køletasken og ved ankomsten til hospitalet overføres prøverne straks til -80 °C-fryser, hvor prøverne gemmes i biobank til senere analyse.

#### Fæcesopsamling til bakteriedyrkning

Afføring opsamles som ovenfor anført til transportcontainer og anbringes herefter i eget køleskab. Forud for transport til Gentofte Hospital overføres transportcontaineren til køletasken og ved ankomsten til hospitalet sendes prøven til undersøgelse på Klinisk mikrobiologisk afdeling, Rigshospitalet.

Foruden afføringsprøver til mikrobiologisk undersøgelse opsamles der forud for antibiotikakuren en afføringsprøve til behandling af eventuel antibiotikaassocieret svær og langvarig diarré. Denne prøve håndteres på samme måde som prøven til bakteriedyrkning beskrevet ovenfor. Prøven kan efter lægelig vurdering og samtykke fra forsøgspersonen genindgives til samme forsøgsperson på rektalsonde, såfremt der måtte være indikation herfor (se afsnittet ”Bivirkninger, risici og ulemper”).

I forbindelse med opsamling af afføringsprøven i slutningen af antibiotikakuren (dag 3) opsamles desuden en ekstra afføringsprøve (tages og behandles svarende til prøverne til DNA-sekventering (se ovenfor)) til bestemmelse af fæces-vancomycinkoncentration med henblik på at vurdere farmakokinetiske aspekter af antibiotikakuren. Bestemmelsen foregår på Klinisk mikrobiologisk afdeling, Rigshospitalet.

### Blodprøver (dag 0, dag 4, dag 8, dag 42 og dag 180)

Blodprøve til oprensning af bakteriel DNA tages på hospitalet i fastetilstanden forud for ad libitum-måltidet. Blodprøvetagning og opbevaring er som med henblik på human DNA-purifikation. I purifikationstrinnet anvendes imidlertid glaskugler for at øge udbyttet af bakteriel DNA.

### Spytprøver(dag 0, dag 4, dag 8, dag 42 og dag 180)

Spytprøver opsamles på hospitalet (på dag 0, dag 4 og dag 42 i minutterne umiddelbart før ad libitum-måltidet). Følgende procedure anvendes: Forsøgspersonen tager et stykke paraffin i munden og tygger i et tempo, som synes naturlig for vedkommende indtil paraffinen er blevet til et sammenhængende stykke (ca. et minut). Forsøgspersonen synker det spyt, som måtte blive produceret i det ene minut. Uret startes, og derefter opsamles det dannede spyt i de næste 3 minutter ved at forsøgspersonen spytter ud i et bæger. Spytprøverne fordeles i to rør af henholdsvis 1,8 ml og 4,5 ml. Det lille rør nedfryses direkte på tøris så hurtigt som muligt, og opbevares derefter ved -80 °C. Det andet rør tilsættes RNA*later* i forholdet 1:3 (spyt:RNA*later*) og stilles i køleskab i ca. 24 timer, hvorefter det fryses til -80 °C indtil analyse.

Af hensyn til bakteriebestemmelsen skal forsøgsdeltagerne undlade at børste tænder om morgenen før spytprøveopsamlingen.

### Urinprøver(dag 0, dag 4, dag 8, dag 42 og dag 180)

Urinprøve fra midtstråle af anden vandladning på dagen opsamles i steril beholder på laboratoriet om morgenen. Prøven nedfryses og opbevares ved -80 °C.

### Optional gastroduodenoskopi (dag -1 (eller før) og dag 3)

Udføres i Endoskopienheden på Gentofte Hospital efter 8 timers faste. Undersøgelsen udføres af en trænet endoskopør efter afdelingens standardinstruks. Undersøgelsen varer cirka 15 minutter, men der påregnes yderligere 5 minutter til forudgående sedation jf. instruks. I forbindelse med undersøgelsen foretages følgende: Der opsamles (aspireres) - såfremt det er muligt - sekret til bestemmelse af bakterieforekomst vurderet ved hjælp af sekventering af bakterie-DNA og dyrkning. Der udtages endvidere 11 standard slimhindebiopsier (ca. 3-5 mg) - 6 til genekspressionsanalyser og 5 til immunohistokemi - fra duodenums andet stykke. Efter undersøgelsen geninstrueres deltageren i, at han ikke må være fører af et motorkøretøj resten af dagen. Gastroduodenoskopi udføres som anført kun på deltagere, som har givet specifikt samtykke til denne undersøgelse. I tilfælde af moderate/svære antibiotikaassocierede bivirkninger på dag 3 (hvor 2. gastroduodenoskopi er planlagt til), vil undersøgelsen blive aflyst. Ligeledes vil undersøgelsen blive aflyst i ethvert tilfælde af almenpåvirkning eller utilpashed hos forsøgsdeltageren.

## RATIONALE BAG STUDIEDESIGN

Der er tale om et fysiologisk interventionsforsøg, hvor deltagerne er deres egne kontroller. Effekten af eradikation af tarmens naturlige bakterieflora vurderes på effektvariable angivet i afsnittet ”Endepunkter” ovenfor. Idet den basale viden på området er yderst sparsom, ønsker vi i første omgang at se på forholdene hos raske unge mænd uden sygdomme, der teoretisk kan tænkes at påvirke resultatet (se eksklusionskriterier). Som et mål for de faktiske ændringer i bakteriefloraen analyseres afføringens, duodenums og mundhulens sammensætning og funktion af bakterier. Den benyttede antibiotika-’*cocktail*’ er et værktøj til at opnå tarmbakterieeradikation og forventes ikke at have terapeutiske effekter eller væsentlige effekter på kroppen i det hele taget.

## BEREGNINGER OG STATISTIK

Det primære endepunkt (postprandialeplasma-GLP-1- og -GIP-responser) vurderes som *area under curve* (AUC) for plasma-GLP-1- og -GIP-værdierne efter måltidsindtagelse. Det samme gælder for de øvrige postprandiale hormonresponser i plasma (sekundære endepunkter). Ændringer i øvrige plasma-/serumkoncentrationer inkl. knogle- og inflammationsmarkører vurderes ved sammenligning af fasteværdier før og efter eradikation af tarmfloraen.

Til volumenbestemmelse af galdeblæren ved hjælp af ultralyd anvendes ellipsoidmetoden. Uddrivningsfraktionen, *ejection fraction* (EF), fra galdeblæren kan bestemmes ved hjælp af følgende formel:


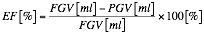


FGV = fastegaldeblærevolumen; PGV = postprandial galdeblærevolumen; EF = *ejection fraction*.

Data vil blive bearbejdet og præsenteret ved hjælp af standard deskriptiv statistik. Sammenligning af normalfordelte data udføres ved hjælp af uparrede to-halede t-tests. Data, der ikke er normalfordelte, sammenlignes ved hjælp af Mann-Whitney U-test. To-faktor *repeated measurement analysis of variance* anvendes til statistisk analyse af gentagne målinger hos den samme forsøgsdeltager.

Ved at inkludere 10 personer i undersøgelsen vil vi med udgangspunkt i standarddeviationen af postprandiale GLP-1-responser (vurderet ved hjælp af AUC_GLP-1_) kunne detektere en mindste relevant differens på 10% for den primære effektparameter (ændring i AUC_GLP-1_efter tarmbakterieeradikation) meden styrke på 85% og et signifikansniveau på 5 %. For at sikre den nævnte styrke samt øge chancerne for at opnår relevante og signifikante fund med hensyn til de sekundære effektparametre inkluderes 12 deltagere i undersøgelsen.

## PRAKTISKE FORANSTALTNINGER

Screening og kliniske eksperimentelle procedurer (fraset gastroduodenoskopi) vil foregå i Diabetologisk Forskningsenhed, Medicinsk afdeling F, Gentofte Hospital, hvor fornødent udstyr og ekspertise forefindes. Gastroduodenoskopierne foretages i Endoskopienheden på Gentofte Hospital, hvor der årligt fortages tusindvis af disse undersøgelser. Plasmakoncentrationsmålinger af inkretinhormonerne GIP og GLP-1, CCK, gastrin, ghrelin, PYY, GLP-2, oxyntomodulin og glukagon vil blive varetaget af professor Jens Juul Holsts laboratorium på Panum (Biomedicinsk Institut, Det Sundhedsvidenskabelige Fakultet, Københavns Universitet), som har årtiers erfaring med disse analyser. Laboratoriet vil også bistå med eksplorative og hypotesegenererende genekspressionsanalyser af væv udtaget i forbindelse med skopier (analyse af kandidatgener samt *microarray*-analyser). Seruminsulin og -C-peptid måles på Klinisk Biokemisk afdeling, Gentofte Hospital. Serumgaldesyrer bestemmes med hjælp fra Klinisk Biokemisk afdeling, Roskilde Hospital. Dyrkning af bakterieprøver foretages på Klinisk mikrobiologisk afdeling, Rigshospitalet. Section of Metabolic Genetics, Novo Nordisk Foundation Center for Basic Metabolic Research, Københavns Universitet, er ansvarlige for *metabolomics*-analyser, DNA-purifikation fra spyt, blod og tarm/fæces og *deep metagenomic next-generation sequencing* af bakterie-DNA samt måling af serum-/plasmamarkører for inflammation.

## PROJEKTETS MULIGE BETYDNING

Studiet vil bidrage med vigtige informationer omkring interaktionen mellem tarmbakterieflora og glukose- og knogleomsætning. Hvis vi finder, at tarmfloraen har væsentlig betydning for knogle- og/eller glukoseomsætningen, vil det være nærliggende at forestille sig nye behandlingsprincipper indenfor hyppige folkesygdomme såsom T2DM og osteoporose, der bygger på modellering af tarmfloraen.

## BIVIRKNINGER, RISICI OG ULEMPER FOR FORSØGSDELTAGERE

Den væsentligste ulempe for den enkelte forsøgsdeltager er risikoen for at udvikle dehydrering i forbindelse med diarré i tilslutning til antibiotikakuren. Symptomer på dehydrering vil blive gennemgået grundigt med deltagerne, og alle deltagere vil blive instrueret i at indtage rigelige mængder væske såfremt antibiotikainduceret diarré skulle opstå. Såfremt tegn på dehydrering alligevel skulle indtræde, skal forsøgspersonen kontakte forsøgsansvarlig læge, der vil træffe beslutning om, hvorvidt forsøgspersonen bør ophøre med antibiotikakuren eller kan fortsætte i undersøgelsen. I tilfælde af længerevarende diarré (efter afslutning af antibiotikakuren) er der mulighed for, at forsøgspersonen kan modtage autolog fæcestransplantation ved hjælp af fæcesprøve opsamlet forud for antibiotikakuren. Denne behandling er velkendt og benyttes typisk ved langvarige behandlingsresistente tilfælde af diarré ved fx gastroenterit og kan bidrage til en hurtigere genetablering af den naturlige bakterieflora^34^. De tre typer antibiotika, som benyttes i denne undersøgelse, optages kun i meget begrænset omfang over tarmvæggen^35^, hvorfor risiciene for systemiske virkninger, bivirkninger og udvikling af allergi formodes at være små. Exanthem og urticaria er beskrevet hos mellem 0,1 og 1 % af patienter som modtog intravenøs Meropenem eller Gentamicin i klinisk praksis (tilsvarende mellem 1 og 10% ved intravenøs vancomycin behandling)^35^. Ved en kortvarig per oral antibiotikakur formodes disse risici at være langt lavere.

I ethvert tilfælde af nyopståede symptomer i forsøgsperioden eller umiddelbart herefter skal forsøgspersonen kontakte forsøgsansvarlig læge, der vil træffe beslutning om videre plan. Tegn på allergisk medikamentel reaktion vil medføre akut lægelig vurdering og behandling. Den videre udredning for eventuel allergi og vejledning herom vil foregå i samråd med allergiklinikken på Gentofte Hospital. Som konsekvensen af påvist allergi kan forsøgspersonen i fremtidige tilfælde af bakteriel infektion være forholdt muligheden for at modtage det pågældende antibiotika. Dette anses som en sjælden bivirkning (anslået under 0,1% risiko) til medvirken i forsøget.

Gastroduodenoskopierne for forsøgspersonen forbundet med minimalt ubehag, idet undersøgelsen foretages i rus med propofol. Teoretisk set vil der ved enhver enteroskopi, hvorunder sedering med propofol benyttes, være risiko for medicinbivirkning, pulmonale problemer (aspirationspneumoni og hypoxi), kardiel arrytmi samt perforation af mavetarmkanalens væg, instrumentlæsion og blødning^36^. Traditionelt angives en komplikationsrisiko på 1 promille og en mortalitetsrate på 1 ud af 10.000 for diagnostisk gastroduodenoskopi^36^. Disse komplikations- og mortalitetsrater kan dog ikke umiddelbart overføres til vores studie, da vi foretager undersøgelsen på raske personer. Reelt må risikoen for komplikationer ved undersøgelserne således betragtes som minimale. Undersøgelsen aflyses som anført i tilfælde af moderate/svære antibiotikaassocierede bivirkninger samt i ethvert tilfælde af påvirket almentilstand hos forsøgsdeltageren.

Som en teoretisk komplikation til anlæggelse af venekatetre (og enhver anden penetration af hud og blodkar med skarpe/spidse genstande) bør nævnes overfladisk flebitis (venebetændelse). Tilstanden er ufarlig. Risikoen for overfladisk flebitis er lille og minimeres ved at følge kliniske standarder for anlæggelse af venekatetre indbefattet dobbelt aftørring af det involverede hudområde med desinficerende sprit og øvrige sterilprocedurer.

Blodtabet ved deltagelse udgør maksimalt 300 ml pr. testdag og således samlet under 900 ml blod fordelt på tre opsamlinger over 6 uger. Hos raske unge mænd er dette ikke forbundet med risici i sig selv. Udtagelsen kan dog føre til træthed umiddelbart efter undersøgelsen. Efter tredje måltidstest tilbydes deltagerne jernbehandling med almindelige jerntabletter.

## FORSØGSDELTAGERENS FYSISKE OG MENTALE INTEGRITET SAMT PRIVATLIVETS FRED

Oplysninger vedrørende den enkelte forsøgsdeltager beskyttes efter lov om behandling af personoplysninger og Sundhedsloven.

## BIOBANK

Det biologiske materiale tildeles af forsøgsansvarlige en kode svarende til den enkelte forsøgsperson samt tidspunktet for udtagningen. Materialet opbevares under sikre og aflåste forhold frem til analysen i en forskningsbiobank. Plasma-/serumprøverne forventes at være færdigbehandlede i løbet af nogle måneder efter de eksperimentelle procedurer, mens andre blodprøver (herunder human leukocyt-DNA) og prøver til *metabolomic*-analyser samt urin-, spyt- og afføringsprøver forventes at være analyseret indenfor 1-5 år. Ekstramateriale vil blive opbevaret i op til 15 år efter forsøgets afslutning med henblik på gentagelse af fejlanalyser og eventuel behov for yderligere analyser. Anvendelse af disse prøver til andet projekt vil kræve en fornyet godkendelse af De Videnskabsetiske Komitéer for Region Hovedstaden. Efter 15 år vil eventuelt tilbageværende biologisk materiale blive destrueret sammen med alle personhenførbare data samt koden til identifikation. Dele af tarmindhold/fæces og spytprøver vil formentlig af forskergruppen blive sendt til udenlandsk laboratorium med henblik på sekventering af bakterie-DNA. Øvrige prøver vil blive analyseret i Danmark. Projektet og biobanken anmeldes til datatilsynet.

## FORSKNINGSGRUPPE

Fra Diabetologisk Forskningsenhed, Medicinsk afdeling F, Gentofte Hospital deltager læge Kristian Hallundbæk Mikkelsen (kontaktperson og klinisk ansvarlig), overlæge, dr. med. Tina Vilsbøll, læge, ph.d. Morten Frost Nielsen (idéophavsmand), 1. reservelæge, ph.d. Filip Krag Knop (idéophavsmand og projektinitiator). Fra Klinisk mikrobiologisk afdeling, Rigshospitalet deltager ledende overlæge Michael Tvede. Fra Section of Metabolic Genetics, Novo Nordisk Foundation Center for Basic Metabolic Research, Københavns Universitet, deltager professor Torben Hansen og professor Oluf Borbye Pedersen. Fra Kirurgisk sektion, Herlev Hospital deltager professor, overlæge dr. med. Jacob Rosenberg og fra Biomedicinsk Institut, Panum, Københavns Universitet, deltager professor, dr. med. Jens Juul Holst.

## DRIFTSUDGIFTER OG ØKONOMISKE FORHOLD

Projektet er initieret af 1. reservelæge, ph.d. Filip Krag Knop, Diabetologisk Forskningsenhed, Medicinsk afdeling F, Gentofte Hospital. Hverken Filip Krag Knop eller den øvrige forskningsgruppe bag projektet har økonomiske interesser i udførelsen eller resultaterne af projektet. Driftsudgifter til utensilier, screeningsblodprøver og bioanalytikerhjælp dækkes af Medicinsk afdeling F, Gentofte Hospital. Projektets øvrige driftsudgifter påtænkes dækket via private og offentlige fonde, der løbende vil blive ansøgt. Fondsmidlerne vil blive indsat på en fondskonto tilknyttet projektet under Medicinsk afdeling F, Gentofte Hospital, som er under hospitalets revision. På nuværende tidspunkt har projektet modtaget støtte fra Medicinsk afdeling F, Gentofte Hospital i form af to måneders løn til den forsøgsansvarlige læge, Kristian Hallundbæk Mikkelsen, til initiering af projektet. Herudover har projektet ikke modtaget finansiel støtte. Ingen i projektgruppen har økonomisk tilknytning til private virksomheder, fonde m.v., som kunne have interesse i forskningsprojektet. Oplysninger om modtaget støtte (navn på støttegiver, støttebeløb herunder udbetalingsmåden) vil blive eftersendt til Videnskabsetisk Komité for Region Hovedstaden med henblik på etisk stillingtagen og godkendelse.

## VEDERLAG OG UDGIFTSGODTGØRELSE TIL FORSØGSDELTAGERE

Der ydes dækning af dokumenterede transportudgifter, som deltagere måtte have i forbindelse med deltagelse. Grundet projektets tidsmæssige omfang for den enkelte deltager ydes der ulempegodtgørelse på kr. 4.000 (beskattes som B-indkomst) til den enkelte deltager samt en ekstragodtgørelse på kr. 1.000 (beskattes som B-indkomst)til de af forsøgsdeltagerne som foruden hovedprotokollen også gennemgår gastroduodenoskopi før og efter eradikationskur. Ulempegodtgørelsen udbetales fra projektets fondskonto til deltagerens NEM-konto, når undersøgelsesforløbet er afsluttet for den enkelte deltager. Såfremt en forsøgsdeltager vælger at træde ud af forsøget før dette er fuldført, vil ulempegodtgørelsens størrelse svare til aktuelle fremmødetid.

## HVERVNING AF DELTAGERE

Deltagere rekrutteres ved hjælp af opslag på Gentofte Hospital samt annoncering (bl.a. på www.forsoegsperson.dk). Se i øvrigt afsnittet ”Retningslinjer for afgivelse af den mundtlige information og indhentelse af samtykke”. Til ovenstående formål vil den vedlagte annoncetekst blive benyttet.

## TILGÆNGELIGHEDEN AF OPLYSNINGER FOR FORSØGSPERSONER

Forsøgsdeltagerne er sikret adgang til at få yderligere oplysninger om projektet. Kontaktperson: læge Kristian Hallundbæk Mikkelsen, Diabetologisk Forskningsenhed, Medicinsk afdeling F, Gentofte Hospital; tlf.: 61 69 97 59; e-mail: kristianmikkel@gmail.com.

## OFFENTLIGGØRELSE AF FORSØGSRESULTATER

Data forventes publiceret i internationale videnskabelige tidsskrifter. Såvel negative som positive forsøgsresultater vil blive offentliggjort. Lov om behandling af personoplysninger vil blive overholdt.

## AFBRYDELSE AF FORSØGET

Forsøget afbrydes for den enkelte deltager i fald denne ønsker at udgå af igangværende protokol eller i fald ekstraordinære omstændigheder umuliggør fuldførelse af forsøget. Ligeledes vil ekstraordinære hændelser, der medfører, at projektet ikke lader sig fuldføre i sin helhed, medføre afbrydelse af forsøget for alle igangværende forsøgsdeltagere.

## SIKKERHEDSFORANSTALTNINGER

Udførelse af gastroduodenoskopi udføres efter standardinstrukser og af læger med stor erfaring for procedurerne. Forsøgspersonerne vil under proceduren være let sederede af trænede narkosesygeplejersker med mulighed for hurtigt tilkald af narkoselæger i tilfælde af uventede virkninger eller komplikationer. Anlæggelse af perifere venekatetre udføres af læger med stor erfaring for procedurerne og steril teknik. Selve forsøget foregår i Diabetologisk Forskningsenhed, Medicinsk afdeling F, Gentofte Hospital, samt i Endoskopienheden, Gentofte Hospital. Før inklusion er forsøgsdeltagerne fundet egnet til deltagelse efter lægeundersøgelse. Forsøgspersoner er dækket af den almindelige patientforsikring. Mulige bivirkninger i forbindelse med antibiotikakuren vil blive meddelt forsøgsansvarlige læge, der så vil tage stilling til videre plan.

## VIDENSKABSETISK REDEGØRELSE

Alle deltagere vil modtage mundtlig og skriftlig information, og der vil foreligge mundtligt og skriftligt samtykke om deltagelse i undersøgelserne. Deltagerne informeres af en læge, der ikke varetager eventuel behandling af deltagerne i det daglige. Protokollen overholder Helsinkideklaration II.

Der er som anført risiko for diarré/løs afføring i tilslutning til antibiotikakuren. Denne ulempe er søgt minimeret ved at gøre antibiotikakuren kort og desuden udføre forsøget på raske unge mænd. Diarré kan resultere i dehydrering. Symptomer på dehydrering vil blive gennemgået grundigt med deltagerne, og alle deltagere vil blive instrueret i at indtage rigelige mængder væske såfremt antibiotikainduceret diarré skulle opstå. Ved mistanke om alvorlige bivirkninger ved antibiotikakuren vil forsøgspersonen straks modtage relevant lægelig behandling og vurdering. Den beskedne risiko for udvikling af allergi overfor de anvendte 3 antibiotika er tidligere beskrevet, mulige allergiske reaktioner vil blive håndteret i samarbejde med allergiklinikken.

Gastroduodenoskopi med biopsier er forbundet med minimalt ubehag idet undersøgelsen udføres i rus med propofol. Der er som ovenfor anført (se ’Bivirkninger, risici og ulemper for forsøgsdeltagerne’) en meget begrænset komplikationsrisiko forbundet med undersøgelsen.

De enkelte forsøgsdage (3 måltidstest) er forbundet med minimalt ubehag for forsøgspersonerne. Eneste ulempe ved deltagelse er stikkene ved anlæggelse af perifere venekanyle til blodprøvetagning. Som en teoretisk komplikation til anlæggelse af perifer venekanyle (og enhver anden penetration af hud og blodkar med skarpe/spidse genstande) bør nævnes overfladisk flebitis (venebetændelse). Tilstanden er ufarlig og selvlimiterende. Bakterielt betinget overfladisk flebitis kan behandles med antibiotika. Risikoen for overfladisk flebitis er lille og minimeres ved at følge kliniske standarder for anlæggelse af perifer venekanyle og blodprøvetagning indbefattet dobbelt aftørring af det involverede hudområde med desinficerende sprit og øvrige sterilprocedurer.

Det samlede blodtab på 6 uger bliver under 900 ml for den enkelte deltager. Kun deltagere med normal hæmoglobin kan deltage. Deltager tilbydes jernbehandling efter forsøget.

Alle deltagere tildeles et forsøgsnummer og vil på dataark og blodprøveglas kun figurere med initialer og forsøgsnummer. Det fulde navn, CPR-nummer og forsøgsnummer opbevares separat. Projektet vil efter godkendelse af De Videnskabsetiske Komitéer for Region Hovedstaden blive tilmeldt Datatilsynet.

I nødvendige tilfælde er deltagerne dækket af den almindelige patientforsikring.

Biologisk materiale vil blive opbevaret i en forskningsbiobank og behandlet som anført i afsnittet ”Biobank”.

Projektet vil ikke komme den enkelte deltager til gode (fraset den almindelige helbredsundersøgelse, som er en del af screeningsbesøget), men det vil belyse interaktionen mellem tarmbakterieflora og udviklingen af T2DM og osteoporose. Viden på dette område kan på langt sigt føre til en forbedret forståelse af patogenesen ved disse sygdomme og dermed skabe mulighed for forbedrede behandlinger. Disse forventelige fordele opvejer efter forskningsgruppens bedste overbevisning det faktum, at projektet formentlig ikke vil komme den enkelte deltager til gode (fraset den almindelige helbredsundersøgelse) samt de minimale risici og bivirkninger og det beskedne ubehag for deltagerne, som projektet er forbundet med (nævnt ovenfor).

## RETNINGSLINJER FOR AFGIVELSE AF DEN MUNDTLIGE INFORMATION OG INDHENTELSE AF SAMTYKKE

Deltagere vil blive rekrutteret ved hjælp af opslag eller ved hjælp af annoncering (bl.a. på www.forsoegsperson.dk). Såfremt forsøgspersonerne responderer på opslag, tager forsøgsansvarlige læge kontakt og afklarer om den eventuelle forsøgsperson ønsker at deltage i en uforpligtende informationssamtale om projektet. Tid og sted aftales. Endvidere oplyses om retten til at medbringe bisidder til samtalen. Inden samtalen vil den skriftlige information om projektet blive fremsendt, dvs. deltagerinformation (bilag 2), samt materialet "Før du beslutter dig" (bilag 8) og "forsøgspersoners rettigheder i et biomedicinsk forskningsprojekt" (bilag 7)

Ved informationssamtalen, der foregår i uforstyrrede fysiske rammer med en person, som har de faglige forudsætninger for at kunne formidle indholdet af forskningsprojektet, og som har direkte tilknytning til forskningsprojektet, angives det først, at der er tale om en forespørgsel om deltagelse i et biomedicinsk forskningsprojekt. Herefter gives den mundtlige information om projektet med udgangspunkt i den skriftlige information og eventuelle spørgsmål besvares. Der oplyses om retten til betænkningstid efter informationssamtalen. Såfremt personerne fortsat er interesserede i at deltage i projektet, aftales tid og sted for indhentning af skriftligt samtykke (forsøgspersonen og den projektansvarlige læge underskriver samtykkeerklæringen). Først derefter foretages studiespecifikke procedurer.

## REFERENCER

1. Rifkin H, Porte D Jr (Eds). *Ellenberg and Rifkin’s Diabetes Mellitus*. New York: Elsevier; 1990.

2. Nolan, C.J., Damm, P. & Prentki, M. Type 2 diabetes across generations: from pathophysiology to prevention and management. *Lancet* **378**, 169-181 (2011).

3. Pocock NA, Eisman JA, Hopper JL, et al. Genetic determinants of bone mass in adults. A twin study. *J Clin Invest.* 1987;80(3):706-710.

4. Hawker, G.A., Jamal, S.A., Ridout, R. & Chase, C. A clinical prediction rule to identify premenopausal women with low bone mass. *Osteoporos Int* **13**, 400-406 (2002).

5. Clemens TL, Karsenty G. The osteoblast: an insulin target cell controlling glucose homeostasis. *J Bone Miner Res.* 2011;26(4):677-680.

6. Turnbaugh, P.J. *et al.* The human microbiome project. *Nature* **449**, 804-810 (2007).

7. Turnbaugh, P.J. *et al.* A core gut microbiome in obese and lean twins. *Nature* **457**, 480-484 (2009).

8. Arumugam, M. *et al.* Enterotypes of the human gut microbiome. *Nature* **473**, 174-180 (2011).

9. Wu, G.D. *et al.* Linking long-term dietary patterns with gut microbial enterotypes. *Science* **334**, 105-108 (2011).

10. Larsen, N. *et al.* Gut microbiota in human adults with type 2 diabetes differs from non-diabetic adults. *PLoS ONE* **5**, e9085 (2010).

11. Vrieze A, Holleman F, Serlie MJ, et al. Metabolic effects of transplanting gut microbiota from lean donors to subjects with metabolic syndrome. *Diabetologia*. 2010;53:(Suppl1):1–556.

12. Turnbaugh, P.J. *et al.* An obesity-associated gut microbiome with increased capacity for energy harvest. *Nature* **444**, 1027-1031 (2006).

13. Membrez, M. *et al.* Gut microbiota modulation with norfloxacin and ampicillin enhances glucose tolerance in mice. *FASEB J.* **22**, 2416-2426 (2008).

14. Hildebrandt MA, Hoffmann C, Sherrill-Mix SA, et al. High-fat diet determines the composition of the murine gut microbiome independently of obesity. *Gastroenterology*. 2009;137(5):1716-1724.

15. Kootte RS, Vrieze A, Holleman F, et al. The therapeutic potential of manipulating gut microbiota in obesity and type 2 diabetes mellitus. *Diabetes, Obes Metab*. Epub aug. 2011.

16. Nauck MA, Bartels E, Orskov C, et al. Additive insulinotropic effects of exogenous synthetic human gastric inhibitory polypeptide and glucagon-like peptide-1-(7-36) amide infused at near-physiological insulinotropic hormone and glucose concentrations. *J Clin Endocrinol Metab.* 1993;76(4):912-917.

17. Roberfroid M, Gibson GR, Hoyles L, et al. Prebiotic effects: metabolic and health benefits. *Br J Nutr.* 2010;104(Suppl 2):1-63.

18. Cani, P.D. *et al.* Improvement of glucose tolerance and hepatic insulin sensitivity by oligofructose requires a functional glucagon-like peptide 1 receptor. *Diabetes* **55**, 1484-1490 (2006).

19. Wostmann BS. Intestinal bile acids and cholesterol absorption in the germfree rat. *J Nutr.* 1973;103(7):982-990.

20. Miyata M, Yamakawa H, Hamatsu M, et al. Enterobacteria modulate intestinal bile acid transport and homeostasis through apical sodium-dependent bile acid transporter (SLC10A2) expression. *J Pharmacol Exp Ther.* 2011;336(1):188-196.

21. Thomas, C. *et al.* TGR5-mediated bile acid sensing controls glucose homeostasis. *Cell Metab.* **10**, 167-177 (2009).

22. Sjögren K, Engdahl C, Lagerquist M, et al. *Amer Soc Bone Miner Res*. Annual Meeting 2010, oral presentation number 1170.

23. Abrams SA, Griffin IJ, Hawthorne KM, et al. A combination of prebiotic short- and long-chain inulin-type fructans enhances calcium absorption and bone mineralization in young adolescents. *Am J Clin Nutr.* 2005;82(2):471-476.

24. Tsukiyama K, Yamada Y, Yamada C, et al. Gastric inhibitory polypeptide as an endogenous factor promoting new bone formation after food ingestion. *Mol Endocrinol.* 2006;20(7):1644-1651.

25. Yamada, C. *et al.* The murine glucagon-like peptide-1 receptor is essential for control of bone resorption. *Endocrinology* **149**, 574-579 (2008).

26. Nelson, R.L., Glenny, A.M. & Song, F. Antimicrobial prophylaxis for colorectal surgery. *Cochrane Database Syst Rev* CD001181 (2009).doi:10.1002/14651858.CD001181.pub3

27. D’Amico, R. *et al.* Effectiveness of antibiotic prophylaxis in critically ill adult patients: systematic review of randomised controlled trials. *BMJ* **316**, 1275-1285 (1998).

28. Bass NM, Mullen KD, Sanyal A, et al. Rifaximin treatment in hepatic encephalopathy. *N Engl J Med.* 2010;362(12):1071-1081.

29. Buzyn A, Tancrède C, Nitenberg G, Cordonnier C. Reflections on gut decontamination in hematology. *Clin Microbiol Infect.* 1999;5(8):449-456.

30. Francois, F. *et al.* The effect of H. pylori eradication on meal-associated changes in plasma ghrelin and leptin. *BMC Gastroenterol* **11**, 37 (2011).

31. Korsgaard H, Agersø Y. DANMAP 2010 - Use of antimicrobial agents and occurrence of antimicrobial resistance in bacteria from food animals, food and humans in Denmark. Statens Serum Institut 2010.

32. www.sst.dk. Tabel 3 incidens af nye diabetikere i Danmark 1997-2009.

33. Christensen K, Bjørk C, Vinter-Larsen M. Otte folkesygdomme - forekomst og udvikling. Statens institut for folkesundhed 2005.

34. Bartlett JG. Narrative review: the new epidemic of Clostridium difficile-associated enteric disease. *Ann Intern Med* 2006;145(10):758-764.

35. www.pro.medicin.dk lægemiddeloplysninger for de enkelte præparater.

36. Cotton P, Williams C. *Practial gastrointestinal endoscopy*. **1996**, (Blackwell Science: ).

# BILAG 1, LÆGMANDSRESUMÉ

**Eradikation af den humane tarmflora**

*Effekt på postprandial tarmhormonsekretion, glukosemetabolisme, knogleomsætning og tarmmikrobiom*

## BAGGRUND OG FORMÅL

Type 2-Diabetes (T2-DM, tidligere kaldet ”gammelmandssukkersyge”) og knogleskørhed, som også kaldes osteoporose, er hyppigt forekommende sygdomme forårsaget af blandt andet arv og livsstilsfaktorer. Indenfor de senere år er man blevet opmærksom på, at tarmens bakterier kan have betydning for udvikling af disse sygdomme. Det er blevet hævdet, at tarmbakterierne kan indvirke mere eller mindre gavnligt på kroppen afhængigt af sammensætningen af bakterierne.

Undersøgelser har vist, at den menneskelige tarmbakterieflora kan hænge sammen med forekomsten af T2-DM; og ved at ændre sammensætningen af tarmbakterierne hos dyr har man kunnet påvirke dyrenes sukkeromsætning. Også ved knogleskørhed har man sandsynliggjort, at en ændret tarmbakterieflora kan påvirke knogleomsætningen hos både dyr og mennesker. Det er kendt, at udskillelsen af en række hormoner, galdesyrer samt visse fedtsyrer påvirkes, når bakteriefloraen i tarmen ændres, men forklaringen bag disse effekter er aktuelt ukendt.

I dette projekt vil vi derfor undersøge, om en midlertidig fjernelse af tarmbakterierne hos mennesker medfører forandringer i sukker- og knogleomsætningen, ligesom vi vil forsøge at afklare, ad hvilke biokemiske veje disse forandringer i givet fald sker. Som led i dette vil vi undersøge, hvordan bakteriefjernelsen indvirker på:

1. udskillelsen af en række tarm-, og sukkerhormoner, foruden markører for opbygningen og nedbrydningen af knoglerne samt betændelse.

2. udskillelsen af galdesyrer fra galdeblæren.

3. appetitregulering og hvilestofskifte.

4. forekomsten af bakterier og betændelsesmarkører i urin og spyt.

5. aktiviteten i forskellige gener i tolvfingertarmen

Vi vil desuden undersøge sammensætningen af bakterierne, når disse er kommet tilbage til tarmsystemet.

## DESIGN

I undersøgelsen skal indgå 12 raske mænd, af dansk etnicitet, i alderen 18-40 år. For at deltage i undersøgelsen må man ikke have sukkersyge eller nogen kendt knoglesygdom, man skal i det daglige indtage en nogenlunde gennemsnitlig, varieret dansk kost (ikke være vegetar, veganer eller spise efter særlig diæt). Desuden udelukkes man fra undersøgelsen, hvis man fejler noget i nyrer, lever, skjoldbruskkirtel og tarmsystemet eller nære slægtninge med arvelige mavetarmsygdomme, hvis man er ryger eller er i behandling med medicin, der kan påvirke det, vi vil undersøge (f.eks. binyrebarkhormon) eller hvis man er allergisk overfor den anvendte medicin.

Undersøgelsen strækker sig over 6 måneder og indebærer for den enkelte deltager i alt 6 besøg på afdelingen samt en 4 dages antibiotikakur i hjemmet.

Personer, som ønsker at deltage i undersøgelsen og som umiddelbart kan indgå i projektet, skal efter afgivelse af informeret samtykke have foretaget en række blodprøver, som skal afklare, om de opfylder kriterierne for deltagelse.

Hvis disse prøver er normale, inkluderes vedkommende i undersøgelsen, og der vil derefter blive foretaget en række undersøgelser:

Ved forsøgets start indsamles afførings-, urin og spytprøve og der foretages en såkaldt måltidstest (beskrevet nedenfor).

Forsøgsdeltageren indtager derefter over 4 dage antibiotika, der fjerner bakterierne i tarmsystemet, og derefter gentages såvel måltidstesten som indsamlingen af afføring, spyt og urin. En uge efter den første måltidstest indsamles igen afføring, spyt og urin og efter 6 uger gentages måltidstesten sammen med indsamling af afføring, urin og spyt. Ét halvt år efter at projektet er startet, indsamles for sidste gang afføring, urin og spyt.

Hos deltagere, der - udover at samtykke til deltagelse i hovedforsøget - samtykker til at få foretaget kikkertundersøgelser af tolvfingertarmen, vil disse blive udført dels på en selvstændig dag før antibiotikakuren (fra efter inklusion frem til dag -1) og på dag 3 (umiddelbart før sidste antibiotikaindtag). For disse deltagere er der således tale om 8 (og ikke 6) besøg på afdelingen.

En oversigt over forsøget er vist herunder:


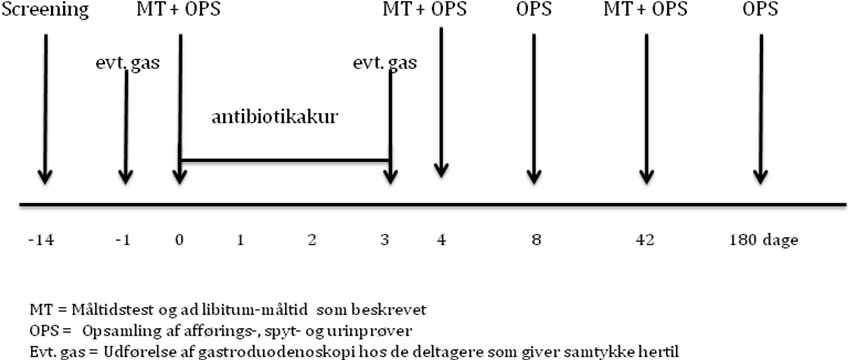


### Undersøgelser og procedurer

På dagen for måltidstesten møder forsøgsdeltageren i laboratoriet klokken 8 efter 10 timers faste. Inden forsøgsstart opsamles en urinprøve og der anlægges en venekanyle (dropnål) i en vene (et tyndt blodkar) i hånden eller underarmen. Herfra tages ca. 40 ml blod til bestemmelse af en række genparametre, betændelsesmarkører samt knoglemarkører i blodet. Der foretages derefter en måling af hvilestofskifte ved at opsamle forsøgsdeltagerens udåndingsluft i en særlig maske. Herefter bestemmes galdeblærens størrelse ved hjælp af en ultralydsscanning og det egentlige forsøg startes: Forsøgspersonen indtager 200 ml Nutridrink (ernæringsdrik med kakaosmag) samt 1,5 gram paracetamol (3 styk almindelige håndkøbssmertestillende tabletter) over ca. 10 minutter. Over de næste 4 timer opsamles løbende en række blodprøver via den anlagte venekanyle ligesom gasmåling på udåndingsluft og ultralydsscanning af galdeblæren gentages nogle gange. Desuden vil forsøgsdeltageren i løbet af de 4 timer blive stillet en række spørgsmål vedrørende appetit og mæthedsfornemmelse.

Blodprøverne skal vise, om sammensætningen af markører for sukker- og knogleomsætningen ændres, ligesom der også måles på udskillelsen af en række tarmhormoner. I alt tages i forbindelse med måltidstest ikke over 300 ml blod. Den løbende ultralydsscanning af galdeblæren skal bestemme den hastighed, hvormed galdeblæren trækker sig sammen.

Når måltidstesten er overstået, vil forsøgspersonerne blive tilbudt et standardmåltid, som de instrueres i at indtage, indtil de føler sig behageligt mætte. I forbindelse med dette stilles igen en række spørgsmål, og størrelsen af det indtagne måltid noteres.

Tarmbakterierne fjernes med 3 lægemidler (antibiotika) (Vancomycin (500mg), Meropenem (500mg) og Gentamicin (40mg)), som opløses i 1 glas (200ml) frugtjuice og drikkes 1 gang dagligt i 4 på hinanden følgende dage. Første dosis indtages umiddelbart efter måltidstestens afslutning, mens anden, tredje og fjerde dosis udleveres (i alt 9 glas) til forsøgspersonerne på 1. forsøgsdag og skal indtages (udenfor laboratoriet) på hver af de følgende 3 dage ved aftenstid, ligeledes opløst i 200 ml juice.

Deltagerne opfordres til ikke at ændre deres fødeindtag i løbet af undersøgelsen. Under de 4 dages antibiotikakur vil forsøgsdeltagere være i daglig telefonisk kontakt med en læge.

Der opsamles afføring, blod, urin og spyt til bakterieanalyser lige før bakteriefjernelse, lige efter-, 8 dage efter-, 42 dage efter- og 180 dage efter bakteriefjernelse. Desuden anvendes afføringsprøverne til bestemmelse af lægemiddelkoncentration og en enkelt prøve fryses til brug ved eventuel udvikling af svær diarré (se herom senere). Spytprøve opsamles ved at stimulere forsøgspersonens spytdannelse med et stykke paraffin som tygges, og herefter lade forsøgspersonen spytte ud i et bæger over de næste 3 minutter. Urin, blod og spytprøve anvendes til bestemmelse af bakterieforekomst samt indhold af en række betændelsesmarkører og cellesignalstoffer.

Hos deltagere der giver særskilt samtykke vil der foruden ovenstående undersøgelser blive foretaget kikkertundersøgelse af tolvfingertarmen (gastroduodenoskopi) før og efter antibiotikakuren. I forbindelse med forsøgspersonens inklusion i studiet vil en læge informere om kikkertundersøgelsen, herunder bivirkninger og risici. Forsøgspersonen har derefter mulighed for at tilvælge kikkertundersøgelsen (i tillæg til det øvrige program). Hvis forsøgspersonen samtykker til kikkertundersøgelsen foretages den som anført på en selvstændig dag før antibiotikakuren (fra efter inklusion frem til dag -1) og på dag 3 (umiddelbart før sidste antibiotikaindtag).

Undersøgelsen udføres i Endoskopienheden på Gentofte Hospital efter 8 timers faste. Den udføres af trænet personale efter afdelingens standardinstruks, hvilket indebærer en kortvarig let bedøvelse med stoffet Propofol. Bedøvelsen kan sammenlignes med en kort slumretilstand, hvor åndedrættet er upåvirket. Undersøgelsen varer ca. cirka 15 minutter, men der påregnes yderligere 5 minutter til forudgående bedøvelse.

I forbindelse med undersøgelsen udtages 11 vævsprøver fra tolvfingertarmens slimhinde til genanalyser og der opsamles sekret til bestemmelse af bakterieforekomst i tolvfingertarmen. Efter undersøgelsen instrueres deltageren i, at han ikke må være fører af et motorkøretøj resten af dagen.

Skulle der indtræde moderate/svære bivirkninger i forbindelse med antibiotikakuren (specielt diarre eller anden almenpåvirkning), vil kikkertundersøgelsen dag 3 blive aflyst. Ligeledes vil kikkertundersøgelsen blive aflyst, hvis forsøgsdeltageren er utilpas eller alment påvirket.

## ETISKE OVERVEJELSER

Den væsentligste ulempe for den enkelte forsøgsdeltager er risikoen for at udvikle diarré i tilslutning til antibiotikakuren. I de 4 dage antibiotikakuren gives, vil forsøgspersonen være i daglig kontakt med den forsøgsansvarlige læge, og skulle der opstå diarré, vil lægen træffe beslutning om, hvorvidt forsøgspersonen skal ophøre med antibiotika kuren eller kan fortsætte uhindret i undersøgelsen. I tilfælde af svær, længerevarende diarré er der mulighed for, at forsøgspersonen kan behandles med en såkaldt "fæcestransplantation", dvs. kan få tilført sin egen bakteriekultur med henblik på hurtig normalisering tarmfunktionen og dermed ophør af diarreen. Dette gøres i almindelige kliniske sammenhænge ved langvarig diarré, der ikke umiddelbart lader sig behandle, og det udføres ved at man indfører den afføringsprøve, deltageren har afgivet ved undersøgelsens start, i endetarmen.

De 3 typer anvendte antibiotika optages kun i meget begrænset omfang over tarmens slimhinde, og kroppens optag af stofferne er således minimalt. Derfor er risikoen for bivirkninger (fraset diarré) også lille. For at opspore og undgå eventuelle medicinbivirkninger vil ethvert tilfælde af nyopståede symptomer under- eller umiddelbart efter antibiotikakuren blive meddelt den forsøgsansvarlige læge i forbindelse med den daglige kontakt. Der er i forbindelse med antibiotikakuren en lille risiko for, at forsøgspersonen udvikler allergi overfor et eller flere af de 3 anvendte antibiotika. Konsekvensen heraf kan være, at forsøgspersonen i fremtidige tilfælde af svære infektioner ikke kan modtage det pågældende antibiotika og da kan være henvist til et antibiotika med ringere effekt (under 0,1% risiko herfor). I tilfælde af allergisk reaktion vil forsøgspersonen blive henvist til videre allergiudredning og rådgivning hos specialafdeling for allergiske sygdomme.

Overfladisk venebetændelse er en teoretisk om end sjælden komplikation til anlæggelse af venekatetre. Tilstanden er ufarlig.

Kikkertundersøgelsen er for forsøgspersonen forbundet med minimalt ubehag, da undersøgelsen foretages i let bedøvelse med propofol. Teoretisk set vil der ved enhver kikkertundersøgelse med bedøvelse være risiko for medicinbivirkning, lungeproblemer (lungebetændelse og iltmangel), hjerterytmeforstyrrelse, instrument læsion og perforation af slimhinden, blødning. Når proceduren udføres ved et planlagt program og på raske unge mennesker, er risikoen for komplikationer ved undersøgelsen minimal. Undersøgelsen gennemføres kun såfremt forsøgsdeltageren føler sig veltilpas, og vil blive aflyst i tilfælde af moderate/svære antibiotikabivirkninger på dag 3.

Blodtabet ved deltagelse udgør maksimalt 300 ml pr testdag og er således samlet under 900 ml blod fordelt på 3 opsamlinger over 6 uger. Hos raske er dette ikke forbundet med risici i sig selv: udtagelsen kan dog føre til træthed umiddelbart efter undersøgelsen. Deltagernes blodprocent vil løbende blive kontrolleret og deltagerne vil evt., efter lægelig vurdering, blive tilbudt tilskud af jerntabletter for at undgå jernmangel.

Anvendelsen af ultralydsscanning samt måling af udåndingsluft er ikke forbundet med nogen form for ubehag eller risiko.

## FORMIDLING

Alle resultater af undersøgelsen formidles i form af artikler, som søges publiceret i internationale tidsskrifter.

## ØKONOMI

Projektet er initieret af 1. reservelæge, ph.d. Filip Krag Knop, Diabetologisk Forskningsenhed, Medicinsk afd. F, Gentofte Hospital. Hverken Filip Krag Knop eller den øvrige forskningsgruppe bag projektet har økonomiske interesser i udførelsen eller resultaterne af projektet.

Driftsudgifter til utensilier, screeningsblodprøver og bioanalytikerhjælp dækkes af Medicinsk afdeling F, Gentofte Hospital. Projektets øvrige driftsudgifter påtænkes dækket via private og offentlige fonde, der løbende vil blive ansøgt. Fondsmidlerne vil blive indsat på en fondskonto tilknyttet projektet under Medicinsk afdeling F, Gentofte Hospital, som er under hospitalets revision.

På nuværende tidspunkt har projektet modtaget støtte fra Medicinsk afdeling F, Gentofte Hospital i form af 2 måneders lægeløn til initiering af projektet. Herudover har projektet ikke modtaget finansiel støtte. Den forsøgsansvarlige har ikke økonomisk tilknytning til private virksomheder, fonde m.v., som har interesser i forskningsprojektet. Oplysninger om modtaget støtte (navn på støttegiver, støttebeløb herunder udbetalingsmåden) vil blive eftersendt til Videnskabsetisk Komité for Region Hovedstaden med henblik på etisk stillingtagen og godkendelse.

## VEDERLAG OG UDGIFTSGODTGØRELSE TIL FORSØGSDELTAGERE

Der ydes dækning af dokumenterede transportudgifter, som deltagere måtte have i forbindelse med deltagelse. Grundet projektets tidsmæssige omfang for den enkelte deltager ydes der ulempegodtgørelse på kr. 4000 beskattes som B-indkomst) til den enkelte deltager (kr. 5.000 til deltagere som samtykker til gastroduodenoskopi før og efter antibiotikakur). Ulempegodtgørelsen udbetales fra projektets fondskonto til deltagerens NEM-konto, når undersøgelsesforløbet er afsluttet for den enkelte deltager. Såfremt en forsøgsdeltager vælger at træde ud af forsøget, før dette er fuldført, vil ulempegodtgørelsens størrelse svare til aktuelle fremmødetid.

#

# BILAG 2, DELTAGERINFORMATION

**Eradikation af den humane tarmflora**

*Effekt på postprandial tarmhormonsekretion, glukosemetabolisme, knogleomsætning og tarmmikrobiom*

#### Forespørgsel om deltagelse i det biomedicinske forskningsprojekt

Vi er en forskningsgruppe bestående af forskere fra

- Diabetologisk Forskningsenhed, Medicinsk afdeling F, Gentofte Hospital,
- Novo Nordisk Foundation Center for Basic Metabolic Research, Københavns Universitet
- Mikrobiologisk afdeling, Rigshospitalet,
- Mavetarmkirurgisk afdeling, Herlev Hospital.

Vi arbejder med at undersøge sammenhængen mellem menneskets tarmbakterieflora og udviklingen af sukkersyge og knogleskørhed.

Vi vil spørge, om du vil være med i et videnskabeligt forsøg, der beskæftiger sig med denne sammenhæng.

På de næste sider beskrives nærmere, hvad forsøget går ud på, og hvordan det udføres. Det er frivilligt at deltage i forsøget, og du kan når som helst trække dig ud af forsøget - også selv om du har skrevet under på at ville deltage.

Tag dig god tid til at læse papirerne, før du beslutter dig. Du vil få mindst to dages betænkningstid, og du har ret til at medbringe et familiemedlem eller en anden bekendt, når du modtager nærmere information om forsøget mundtligt

MVH

Læge Kristian Hallundbæk Mikkelsen

(kristianmikkel@gmail, telefon61699759) (kontaktperson og forsøgsansvarlig)

1. reservelæge, ph.d. Filip Krag Knop (projektinitiator)

Post.doc, læge, ph.d. Morten Frost Nielsen

Diabetologisk Forskningsenhed, Gentofte Hospital

## BAGGRUND OG FORMÅL

Type 2-Diabetes (T2-DM, tidligere kaldet ”gammelmandssukkersyge”) og knogleskørhed, som også kaldes osteoporose, er hyppigt forekommende sygdomme forårsaget af blandt andet arv og livsstilsfaktorer. Indenfor de senere år er man blevet opmærksom på, at tarmens bakterier kan have betydning for udvikling af disse sygdomme. Det er blevet hævdet, at tarmbakterierne kan indvirke mere eller mindre gavnligt på kroppen afhængigt af sammensætningen af bakterierne.

Studier har vist, at den menneskelige tarmbakterieflora synes at hænge sammen med forekomsten af T2-DM; og ved at ændre sammensætningen af tarmbakterierne hos dyr har man kunnet påvirke dyrenes sukkeromsætning. Også ved knogleskørhed har man påvist, at en ændret tarmbakterieflora kan påvirke knogleomsætningen hos både dyr og mennesker. Det er kendt, at udskillelsen af en række hormoner, galdesyrer samt visse fedtsyrer påvirkes, når bakteriefloraen i tarmen ændres, men forklaringen bag disse effekter er aktuelt ukendt.

I dette projekt vil vi derfor undersøge, om en midlertidig fjernelse af tarmbakterierne hos mennesker medfører forandringer i sukker- og knogleomsætningen, ligesom vi vil forsøge at afklare, ad hvilke biokemiske veje disse forandringer i givet fald sker. Som led i dette vil vi undersøge, hvordan bakteriefjernelsen indvirker på:

1. udskillelsen af en række tarm-, og sukkerhormoner, foruden markører for opbygningen og nedbrydningen af knoglerne samt betændelse.

2. udskillelsen af galdesyrer fra galdeblæren.

3. appetitregulering og hvilestofskifte.

4. forekomsten af bakterier og betændelsesmarkører i urin og spyt.

Vi vil desuden undersøge sammensætningen af bakterierne, når disse er kommet tilbage til tarmsystemet.

Til dette formål ønsker vi at undersøge 12 raske mænd, af etnisk dansk oprindelse i alderen 18-40 år.

For at deltage som forsøgsperson er det et krav, at du:

- ikke har sukkersyge eller knoglesygdom,
- ikke har lever- eller nyresygdom
- ikke har lav blodprocent
- ikke har fået antibiotika (heller ikke malariaforebyggelse) indenfor de seneste 6 måneder
- ikke har mavetarmsygdomme eller har nære slægtninge med arvelige mavetarmsygdomme
- ikke er undervægtig (BMI <18.5 kg/m2) eller overvægtig (BMI >25 kg/m2)
- ikke er allergisk overfor de antibiotika (Vancomycin, Gentamycin eller Meropenem) vi skal anvende i forsøget eller er allergisk overfor såkaldte beta-lactamantibiotika (herunder almindelig penicillin)
- ikke er ryger og ikke spiser efter særlig diæt

## FORSØGETS OPBYGNING

Undersøgelsen strækker sig over 6 måneder og indebærer for dig som deltager i alt 6 besøg på afdelingen samt en 4 dages antibiotikakur i hjemmet. Hvis du - udover at samtykke til deltagelse i hovedforsøget - samtykker til at få foretaget kikkertundersøgelser af din tolvfingertarm, vil disse undersøgelser blive udført dels på en selvstændig dag før antibiotikakuren (fra efter inklusion frem til dag -1) og på dag 3 (umiddelbart før sidste antibiotikaindtag). For dig som deltager i supplerende kikkertundersøgelser er der således tale om 8 (og ikke 6) besøg på afdelingen.

Herunder er vist en oversigt over forsøget og beskrevet hvad der foregår på de enkelte dage. Forud for programmet vil du blive skriftligt og mundtligt informeret omkring forsøget og du skal give skriftligt samtykke til medvirken i forsøget.


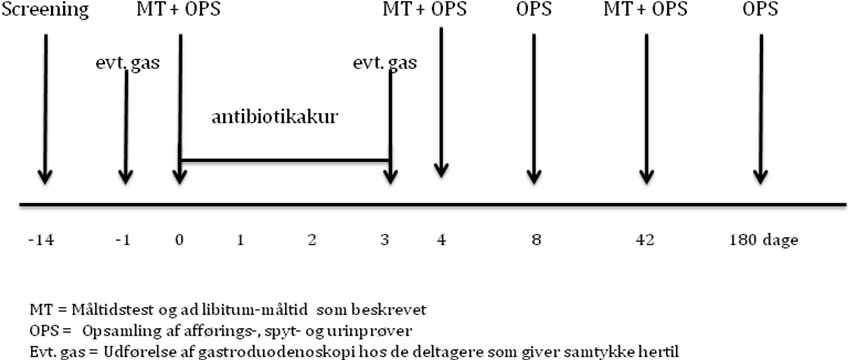


### Screening

Som potentiel forsøgsdeltager møder du i laboratoriet efter 10 timers faste. Her ses du af en læge; vægt, højde og blodtryk noteres og vi tager en række screeningsblodprøver (ca. 10 ml blod) og stiller en række spørgsmål med henblik på at sikre at de ovenforstående krav til forsøgsdeltageren er overholdt.

Såfremt du på basis af resultaterne fra screeningsmødet kan fortsætte i projektet, aftales datoer for undersøgelsesdagene. Hvis blodprøveresultaterne er unormale, rådgives du med hensyn til videre udredning.

### Måltidstest

På dagen for måltidstesten møder du i laboratoriet klokken 8 efter 10 timers faste. Inden forsøgsstart opsamles en urinprøve, og der anlægges en venekanyle (dropnål) i en vene (et tyndt blodkar) i hånden eller underarmen. Herfra tages ca. 40 ml blod til bestemmelse af en række genparametre, bakteriegener, betændelsesmarkører samt knogleskørhedsmarkører i blodet. Der foretages nu en måling af dit hvilestofskifte ved at opsamle din udåndingsluft i en særlig maske. Herefter bestemmes galdeblærens størrelse ved hjælp af en ultralydsscanning, og det egentlige forsøg startes: Du indtager 200 ml Nutridrink (ernæringsdrik med kakaosmag) samt 1,5 gram paracetamol (3 styk almindelige håndkøbssmertestillende tabletter) over ca. 10 minutter. Over de næste 4 timer opsamles løbende en række blodprøver via den anlagte venekanyle ligesom gasmåling på udåndingsluft og ultralydsscanning af galdeblæren gentages nogle gange. Desuden vil vi i løbet af de 4 timer stille dig en række spørgsmål vedrørende appetit og mæthedsfornemmelse.

Blodprøverne skal vise, om sammensætningen af markører for sukker- og knogleomsætningen ændres, ligesom der også måles på udskillelsen af en række tarmhormoner. I alt tages i forbindelse med måltidstest ikke over 300 ml blod. Den løbende ultralydsscanning af galdeblæren skal bestemme den hastighed hvormed galdeblæren trækker sig sammen.

Når måltidstesten er overstået, vil du blive tilbudt et standardmåltid, som du instrueres i at indtage, indtil du føler dig behageligt mæt. I forbindelse med dette stilles du igen en række spørgsmål, og størrelsen af det indtagne måltidet noteres.

### Antibiotikakur

Tarmbakterierne fjernes med 3 lægemidler (antibiotika) (Vancomycin (500mg), Meropenem (500mg) og Gentamicin (40mg)), som opløses i 1 glas (200ml) frugtjuice og drikkes 1 gang dagligt i 4 på hinanden følgende dage. Første dosis indtages umiddelbart efter måltidstestens afslutning, mens anden, tredje og fjerde dosis udleveres (i alt 9 glas) til dig på 1. forsøgsdag og skal indtages (udenfor laboratoriet) på hver af de følgende 3 dage ved aftenstid, ligeledes opløst i 200 ml juice.

Du opfordres til ikke at ændre dit fødeindtag i løbet af undersøgelsen.

Under de 4 dages antibiotikakur vil du dagligt blive telefonisk kontaktet af en læge.

### Opsamling af afførings-, blod-, urin- og spytprøver

Der opsamles afføring, blod, urin og spyt til bakterieanalyser lige før bakteriefjernelsen, lige efter-, 8 dage efter-, 42 dage efter- og 180 dage efter bakteriefjernelsen. Desuden anvendes afføringsprøverne til bestemmelse af lægemiddelkoncentration, og en enkelt prøve fryses til brug ved eventuel udvikling af svær diarré (se herom senere). Spytprøve opsamles ved at stimulere din spytdannelse med et stykke paraffin som tygges, og herefter lade dig spytte ud i et bæger over de næste 3 minutter. Urin og spytprøve anvendes til bestemmelse af bakterieforekomst samt indhold af en række betændelsesmarkører og cellesignalstoffer. Om morgenen før opsamlingen af spytprøven skal du undlade at børste tænder idet dette kan påvirke resultatet af bakteriebestemmelsen.

### Kikkertundersøgelse af tolvfingertarmen

Hos udvalgte forsøgsdeltagere vil der foruden ovenstående undersøgelser blive foretaget kikkertundersøgelse af tolvfingertarmen (gastroduodenoskopi) før og efter antibiotikakuren. I forbindelse med din inklusion i studiet vil en læge informere om kikkertundersøgelsen, herunder bivirkninger og risici. Herefter har du mulighed for at tilvælge kikkertundersøgelsen (i tillæg til det øvrige program). Hvis du samtykker til kikkertundersøgelsen foretages den som anført på en selvstændig dag før antibiotikakuren (fra efter inklusion frem til dag -1) og på dag 3 (umiddelbart før sidste antibiotikaindtag).

Undersøgelsen udføres i Endoskopienheden på Gentofte Hospital efter 8 timers faste. Den udføres af trænet personale efter afdelingens standardinstruks, hvilket indebærer en kortvarig let bedøvelse med stoffet Propofol. Bedøvelsen kan sammenlignes med en kort slumretilstand, hvor åndedrættet er upåvirket. Undersøgelsen varer ca. cirka 15 minutter, men der påregnes yderligere 5 minutter til forudgående bedøvelse.

I forbindelse med undersøgelsen udtages 11 vævsprøver fra tolvfingertarmens slimhinde til genanalyser, og der opsamles sekret til bestemmelse af bakterieforekomst i tolvfingertarmen. Efter undersøgelsen instrueres du i, at du ikke må være fører af et motorkøretøj resten af dagen.

Skulle der indtræde moderate/svære bivirkninger i forbindelse med antibiotikakuren (specielt diarre eller anden almenpåvirkning), vil kikkertundersøgelsen dag 3 blive aflyst. Ligeledes vil kikkertundersøgelsen blive aflyst, hvis du er utilpas eller alment påvirket.

## BIVIRKNINGER, RISICI OG ULEMPER FOR FORSØGSDELTAGERE

Den væsentligste ulempe for dig er risikoen for at udvikle diarré i tilslutning til antibiotikakuren. I de 4 dage antibiotikakuren gives, vil du være i daglig kontakt med den forsøgsansvarlige læge, og skulle der opstå diarré, vil lægen træffe beslutning om, hvorvidt du skal ophøre med antibiotika kuren eller kan fortsætte uhindret i undersøgelsen. I tilfælde af svær, længerevarende diarré er der mulighed for, at du kan blive behandlet med en såkaldt "fæcestransplantation", dvs. kan få tilført din egen bakteriekultur med henblik på hurtig normalisering tarmfunktionen og dermed ophør af diarreen. Dette gøres i almindelige kliniske sammenhænge ved langvarig diarré, der ikke umiddelbart lader sig behandle, og det udføres ved at man indfører den afføringsprøve, du har afgivet ved undersøgelsens start, i endetarmen.

De 3 typer anvendte antibiotika optages kun i meget begrænset omfang over tarmens slimhinde, og kroppens optag af stofferne er således minimalt. Derfor er risikoen for bivirkninger (fraset diarré) også lille. For at opspore og undgå eventuelle medicinbivirkninger vil ethvert tilfælde af nyopståede symptomer under- eller umiddelbart efter antibiotikakuren blive meddelt den forsøgsansvarlige læge i forbindelse med den daglige kontakt. Der er i forbindelse med antibiotikakuren en lille risiko for, at du udvikler allergi overfor et eller flere af de 3 anvendte antibiotika. Konsekvensen heraf kan være, at du i fremtidige tilfælde af svære infektioner ikke kan modtage det pågældende antibiotika og da kan være henvist til et antibiotika med ringere effekt (under 0,1% risiko herfor). I tilfælde af at du udvikler en allergisk reaktion, vil du blive henvist til videre allergiudredning og rådgivning hos specialafdeling for allergiske sygdomme.

Overfladisk venebetændelse er en teoretisk om end sjælden komplikation til anlæggelse af venekatetre. Tilstanden er ufarlig.

Kikkertundersøgelsen er for dig forbundet med minimalt ubehag, da undersøgelsen foretages i let bedøvelse med propofol. Teoretisk set vil der ved enhver kikkertundersøgelse med bedøvelse være risiko for medicinbivirkning, lungeproblemer (lungebetændelse og iltmangel), hjerterytmeforstyrrelse, instrument læsion og perforation af slimhinden, blødning. Når proceduren udføres ved et planlagt program og på raske unge mennesker, er risikoen for komplikationer ved undersøgelsen minimal.

Undersøgelsen gennemføres kun såfremt du føler dig veltilpas og vil blive aflyst i tilfælde af moderate/svære antibiotikabivirkninger på dag 3.

Blodtabet ved deltagelse udgør maksimalt 300 ml pr testdag og er således samlet under 900 ml blod fordelt på 3 opsamlinger over 6 uger. Hos raske er dette ikke forbundet med risici i sig selv: udtagelsen kan dog føre til træthed umiddelbart efter undersøgelsen. Din blodprocent vil løbende blive kontrolleret og du vil evt., efter lægelig vurdering, blive tilbudt tilskud af jerntabletter med henblik på hurtig genopretning af et blodtabet.

Anvendelsen af ultralydsscanning samt måling af udåndingsluft er ikke forbundet med nogen form for ubehag eller risiko.

## ANONYMISERING

Personidentificerbare data og prøver anonymiseres efter projektets afslutning. Ekstra biologisk materiale (blodprøver, spytprøver, afføringsprøver, vævsprøver og sekreter) vil blive opbevaret i op til 15 år efter forsøgets afslutning med henblik på gentagelse af eventuelle fejlanalyser og eventuelt behov for yderligere analyser. Efter 15 år destrueres disse prøver. Anvendelse af disse prøver til et nyt projekt vil kræve en fornyet godkendelse af De Videnskabsetiske Komitéer for Region Hovedstaden. Dele af det udtagne materiale (tarmsekret/afføring og spyt) vil blive sendt til udlandet til laboratorieanalyse, hvor materialet vil være omfattet af det pågældende lands lovgivning på området.

## FORDELE OG ULEMPER

Projektet vil ikke komme dig til gode (fraset den almindelige helbredsundersøgelse, som er en del af screeningsbesøget), men det vil belyse sammenhængen mellem tarmbakterieflora og sukkersyge og knogleskørhed, og derigennem muligvis bidrage til udviklingen af nye behandlingsmuligheder og forbedre de eksisterende for patienter med sukkersyge og knogleskørhed.

## DINE RETTIGHEDER SAMT UDTRÆDELSE OG AFBRYDELSE AF FORSØGET

Inden deltagelse i undersøgelsen bør du som potentiel deltager læse *Forsøgspersonens rettigheder i et biomedicinsk forskningsprojekt* og *Før du beslutter dig* (begge udgivet af Den Centrale Videnskabsetiske Komité og udleveret sammen med resten af informationsmaterialet). Heri er det beskrevet, at du som forsøgsperson uden yderligere begrundelser til en hver tid kan træde ud af undersøgelsen. Ekstraordinære omstændigheder, der umuliggør fuldførelse af forsøget for dig vil resultere i afbrydelse af det pågældende forsøgsforløb. Ligeledes vil ekstraordinære hændelser, der medfører, at projektet ikke lader sig fuldføre i sin helhed, føre til afbrydelse af forsøget for alle igangværende forsøgsdeltagere. Afbrydelse af forsøget vil afstedkomme en grundig information om årsagen hertil til alle involverede forsøgsdeltagere.

I tilfælde af utilsigtede hændelser dækker den almindelige patientforsikring. For rettigheder vedrørende aktindsigt, klageadgang og erstatning henvises til den vedlagte skrivelse *Forsøgspersoners rettigheder i et biomedicinsk forskningsprojekt* fra Den Centrale Videnskabsetiske Komité. Ønsker du information om resultatet af undersøgelserne, kan denne fås ved henvendelse til forsøgsansvarlige læge Kristian Hallundbæk Mikkelsen (se kontaktoplysninger på forsiden).

## GODKENDENDE MYNDIGHED OG ØKONOMI

Projektet er anmeldt til De Videnskabsetiske Komitéer for Region Hovedstaden og meldt til datatilsynet.

Projektet er initieret af 1. reservelæge, ph.d. Filip Krag Knop, Diabetologisk Forskningsenhed, Medicinsk afd. F, Gentofte Hospital. Hverken Filip Krag Knop eller den øvrige forskningsgruppe bag projektet har økonomiske interesser i udførelsen eller resultaterne af projektet. Projektet påtænkes finansieret via fondsmidler fra private og offentlige fonde uden økonomisk tilknytning til forskergruppen bag forskningsprojektet. Bevillingsgivere vil ikke få indflydelse på offentliggørelse af de opnåede resultater. Modtagne midler indsættes på en fondskonto tilknyttet til projektet. Kontoen administreres af Gentofte Hospital. Hverken medicinalindustrien eller øvrige private virksomheder er involveret i projektet.

Grundet projektets tidsmæssige omfang for dig yder vi ulempegodtgørelse på kr. 4000 (beskattes som B-indkomst) for din deltagelse (kr. 5.000 hvis du samtykker til gastroduodenoskopi før og efter antibiotikakur). Ulempegodtgørelsen udbetales fra projektets fondskonto til deltagerens NEM-konto, når undersøgelsesforløbet er afsluttet for den enkelte deltager. Såfremt du vælger at træde ud af forsøget før dette er fuldført, vil ulempegodtgørelsens størrelse svare til aktuelle fremmødetid.

## KONTAKTPERSON PÅ PROJEKTET

Projektets kontaktperson er læge Kristian Hallundbæk Mikkelsen, som også er klinisk ansvarlig læge. Ethvert spørgsmål vedrørende projektet eller deltagelse besvares med glæde på e-mail: kristianmikkel@gmail.com eller telefon: 61699759.

#

# BILAG 3, SAMTYKKEERKLÆRING, DELTAGELSE I FORSKNINGSPROJEKT

**Eradikation af den humane tarmflora**

*Effekt på postprandial tarmhormonsekretion, glukosemetabolisme, knogleomsætning og tarmmikrobiom*

Erklæring fra forsøgspersonen:

Jeg har læst den skriftlige information om forskningsprojektet og fået mundtlig information i et sprog, som jeg forstår. Jeg ved nok om formålet, metoderne, fordele og ulemper til at sige ja til at deltage. Jeg er informeret om, at det er frivilligt at deltage, og at jeg når som helst og uden begrundelse kan trække mit samtykke tilbage og udtræde af forsøget, uden at dette påvirker min ret til behandling eller andre rettigheder.

Jeg indvilger i at deltage i forskningsprojektet og har modtaget en kopi af dette samtykkeark samt en kopi af den skriftlige information til eget brug.

- Jeg ønsker oplysninger om egen helbredstilstand, der måtte fremkomme under gennemførelsen af projektet (sæt kryds):

Ja: ⁭ Nej:⁭

- Jeg ønsker information om de i projektet opnåede resultater, herunder evt. konsekvenser for mig (sæt kryds):

Ja:⁭ Nej:⁭

- Jeg giver hermed tilladelse til at materiale udtaget under forsøgets gennemførelse opbevares med henblik på senere analyser (sæt kryds):

Ja:⁭ Nej:⁭

Forsøgspersonens navn

Dato Underskrift

Erklæring fra den forsøgsansvarlige:

Jeg erklærer, at nedenstående forsøgsperson har modtaget mundtlig og skriftlig information om forskningsprojektet. Efter min bedste overbevisning er der givet tilstrækkelig information, herunder om fordele og ulemper, til at træffe et informeret valg.

Den forsøgsansvarliges navn

Dato Underskrift

# BILAG 4, SAMTYKKEERKLÆRING, GENNEMFØRELSE AF KIKKERTUNDERSØGELSER

**Eradikation af den humane tarmflora**

*Effekt på postprandial tarmhormonsekretion, glukosemetabolisme, knogleomsætning og tarmmikrobiom*

Erklæring fra forsøgspersonen:

Jeg har læst den skriftlige information om gastroduodenoskopi (kikkertundersøgelse af tolvfingertarm) og fået mundtlig information i et sprog, som jeg forstår. Jeg ved nok om formålet, metoderne, fordele og ulemper til at sige ja til at deltage. Jeg er informeret om, at det er frivilligt at deltage, og at jeg når som helst og uden begrundelse kan trække mit samtykke tilbage og udtræde af forsøget, uden at dette påvirker min ret til behandling eller andre rettigheder. Jeg er desuden bekendt med at mit samtykke til gennemførelse af gastroduodenoskopi ikke er en forudsætning for at kunne deltage i forskningsprojektet "Eradikation af den humane tarmflora. Effekt på postprandial tarmhormonsekretion, glukosemetabolisme, knogleomsætning og tarmmikrobiom".

Jeg indvilger i at deltage i gastroduodenoskopi før og efter antibiotikakur og har modtaget en kopi af dette samtykkeark samt en kopi af den skriftlige information til eget brug.

- Jeg ønsker oplysninger om egen helbredstilstand, der måtte fremkomme under gennemførelsen af kikkertundersøgelsen (sæt kryds):

Ja: ⁭ Nej:⁭

- Jeg giver hermed tilladelse til at materiale udtaget under kikkertundersøgelserne opbevares med henblik på senere analyser (sæt kryds):

Ja:⁭ Nej:⁭

Forsøgspersonens navn

Dato Underskrift

Erklæring fra den forsøgsansvarlige:

Jeg erklærer, at nedenstående forsøgsperson har modtaget mundtlig og skriftlig information om forskningsprojektet. Efter min bedste overbevisning er der givet tilstrækkelig information, herunder om fordele og ulemper, til at træffe et informeret valg.

Den forsøgsansvarliges navn

Dato Underskrift

# BILAG 5, ANNONCETEKST PÅ WWW.FORSOEGSPERSON.DK SAMT TEKST I OPSLAG PÅ GENTOFTE HOSPITAL

**Eradikation af den humane tarmflora**

*Effekt på postprandial tarmhormonsekretion, glukosemetabolisme, knogleomsætning og tarmmikrobiom*

**Deltagere til videnskabelig undersøgelse søges:**

Vi er en forskningsgruppe som undersøger sammenhængen mellem tarmens bakterieflora og udviklingen af sukkersyge og knogleskørhed.

Vi ønsker i et nyt forsøg at fjerne den normalt forekommende tarmflora for at se, hvordan dette påvirker omsætningen af sukker, knogle og en række hormoner og signalstoffer i kroppen.

Bakterierne vil blive fjernet ved hjælp af en 4 dages kur med antibiotika (antibakteriemedicin) som drikkes. Før og efter fjernelsen vil vi foretage en række undersøgelser; vi vil opsamle afførings, spyt og urinprøver og foretage en række blodprøver i forbindelse med et testmåltid. Dem der måtte give et særskilt samtykke til det, vil også få foretaget en kikkertundersøgelse af deres tolvfingertarm.

Til formålet ønsker vi at undersøge 12 raske mænd, af kaukasisk oprindelse i alderen 18-40 år.

For at kunne deltage som forsøgsperson er det et krav at du:

- ikke har sukkersyge eller knoglesygdom,
- ikke har lever- eller nyresygdom
- ikke har lav blodprocent
- ikke har fået antibiotika (heller ikke malariaforebyggelse) indenfor de seneste 6 måneder
- ikke har mavetarmsygdomme eller har nære slægtninge med arvelige mavetarmsygdomme
- ikke er undervægtig (BMI <18.5 kg/m2) eller overvægtig (BMI >25 kg/m2)
- ikke er allergisk overfor de antibiotika (Vancomycin, Gentamycin eller Meropenem) vi skal anvende i forsøget eller er allergisk overfor såkaldte beta-lactamantibiotika (herunder almindelig penicillin)
- ikke er ryger og ikke spiser efter særlig diæt

Forsøget forløber over et halvt år og indebærer i alt 6 besøg i vores laboratorium. 5 gange skal forsøgsdeltageren opsamle og aflevere afførings, urin og spytprøver og 3 gange vil vi udføre en måltidsundersøgelse. De 3 forsøgsdage med måltidsundersøgelsen varer ca. 6 timer mens de øvrige 3 besøg er af ganske kort varighed.

Vi tilbyder et vederlag for din tid i forbindelse med forsøgsdeltagelse.

Hvis du eventuelt kunne være interesseret i at deltage og opfylder ovenstående kriterier, er du velkommen til at kontakte den forsøgsansvarlige læge for mere uforpligtende information omkring projektet.

MVH

Kristian Hallundbæk Mikkelsen

Forsøgsansvarlig læge

Diabetologisk Forskningsenhed, Gentofte Hospital

mail: kristianmikkel@gmail.com

telefon: 61699759

1. reservelæge, ph.d. Filip Krag Knop

Post.doc, læge, ph.d. Morten Frost Nielsen

Diabetologisk Forskningsenhed, Gentofte Hospital

# BILAG 6, KOSTREGISTRERINGSSKEMA

**Eradikation af den humane tarmflora**

*Effekt på postprandial tarmhormonsekretion, glukosemetabolisme, knogleomsætning og tarmmikrobiom*

**Du bedes udfyld skemaet vedrørende dit fødeindtag vedrørende dit fødeindtag over de sidste 4 dage. Hvor mange gange har du spist følgende:**

En ring i hver linje

|  |  | 0 | 1-2  gange i løbet af de sidste 4 dage | 3-4  gange i løbet af de sidste 4 dage | 1  gang pr. dag | 2  eller flere gange pr. dag |
| --- | --- | --- | --- | --- | --- | --- |
| **a.** | **Plantemargarine** | 0 | 1 | 2 | 3 | 4 |
| **b.** | **Smør** | 0 | 1 | 2 | 3 | 4 |
| **c.** | **Ost** | 0 | 1 | 2 | 3 | 4 |
| **d.** | **Mælk** | 0 | 1 | 2 | 3 | 4 |
| **e.** | **Yoghurt, inkl. Actimel, cultura og A38** | 0 | 1 | 2 | 3 | 4 |
| **f.** | **Rugbrød, fuldkorn** | 0 | 1 | 2 | 3 | 4 |
| **g.** | **Rugbrød, uden kerner** | 0 | 1 | 2 | 3 | 4 |
| **h.** | **Havregrød** | 0 | 1 | 2 | 3 | 4 |
| **i.** | **Hvidt brød (normal)** | 0 | 1 | 2 | 3 | 4 |
| **j.** | **Hvidt brød (fuldkorn)** | 0 | 1 | 2 | 3 | 4 |
| **k.** | **Kartofler** | 0 | 1 | 2 | 3 | 4 |
| **l.** | **Grøntsager (kogte)** | 0 | 1 | 2 | 3 | 4 |
| **m.** | **Grøntsager (rå)** | 0 | 1 | 2 | 3 | 4 |
| **n.** | **Frisk frugt** | 0 | 1 | 2 | 3 | 4 |
| **o.** | **Juice** | 0 | 1 | 2 | 3 | 4 |
| **p.** | **Ris** | 0 | 1 | 2 | 3 | 4 |
| **q.** | **Spaghetti/pasta** | 0 | 1 | 2 | 3 | 4 |
| **r.** | **Leverpostej** | 0 | 1 | 2 | 3 | 4 |
| **s.** | **Kød** | 0 | 1 | 2 | 3 | 4 |
| **t.** | **Æg** | 0 | 1 | 2 | 3 | 4 |
| **u.** | **Fisk** | 0 | 1 | 2 | 3 | 4 |
| **v.** | **Pølse** | 0 | 1 | 2 | 3 | 4 |
| **x.** | **Kage** | 0 | 1 | 2 | 3 | 4 |
| **y.** | **Marmelade** | 0 | 1 | 2 | 3 | 4 |
| **z.** | **Sodavand, is** | 0 | 1 | 2 | 3 | 4 |
| **æ.** | **Chokolade, slik** | 0 | 1 | 2 | 3 | 4 |

**Har du ændret dine spisevaner i løbet af de sidste 4 dage**

Ja 1

Nej 2

Ved ikke 8

# BILAG 7, FORSØGSPERSONERS RETTIGHEDER I ET BIOMEDICINSK FORSKNINGSPROJEKT

**Eradikation af den humane tarmflora**

*Effekt på postprandial tarmhormonsekretion, glukosemetabolisme, knogleomsætning og tarmmikrobiom*

**DET VIDENSKABSETISKE KOMITÉSYSTEM**

**Forsøgspersonens rettigheder i et biomedicinsk forskningsprojekt.**

Som deltager i et biomedicinsk forskningsprojekt skal du vide at:

- din deltagelse i forskningsprojektet er helt frivillig og kan kun ske efter, at du har fået både skriftlig og mundtlig information om forskningsprojektet og underskrevet samtykkeerklæringen
- du til enhver tid mundtligt, skriftligt eller ved anden klar tilkendegivelse kan trække dit samtykke til deltagelse tilbage og udtræde af forskningsprojektet. Såfremt du trækker dit samtykke tilbage påvirker dette ikke din ret til nuværende eller fremtidig behandling eller andre rettigheder, som du måtte have
- du har ret til at tage et familiemedlem, en ven eller en bekendt med til informationssamtalen
- du har ret til betænkningstid, før du underskriver samtykkeerklæringen
- oplysninger om dine helbredsforhold, øvrige rent private forhold og andre fortrolige oplysninger om dig, som fremkommer i forbindelse med forskningsprojektet, er omfattet af tavshedspligt
- opbevaring af oplysninger om dig, herunder oplysninger i dine blodprøver og væv, sker efter reglerne i lov om behandling af personoplysninger og sundhedsloven
- der er mulighed for at få aktindsigt i forsøgsprotokoller efter offentlighedslovens bestemmelser. Det vil sige, at du kan få adgang til at se alle papirer vedrørende din deltagelse i forsøget, bortset fra de dele, som indeholder forretningshemmeligheder eller fortrolige oplysninger om andre
- der er mulighed for at klage og få erstatning efter reglerne i lov om klage og erstatningsadgang inden for sundhedsvæsenet
