# Supplementary material for: Effect of Antibiotics on Gut Microbiota, Gut Hormones and Glucose Metabolism
Source: PLoS One. 2015 Nov 12;10(11):e0142352. doi: 10.1371/journal.pone.0142352 (PMC4643023; doi:10.1371/journal.pone.0142352)
Supplement: S1 Protocol — (DOCX) [file pone.0142352.s004.docx]

**Eradication of the human intestinal flora

Effect on postprandial tarmhormonsekretion, glucose metabolism, bone turnover and gutmikrobiome


Material for the Research Ethics Committee of the Capital Region
Version 3**


A collaboration between


Diabetes Research Division, Gentofte Hospital, University of Copenhagen
Kristian Hallundbæk Mikkelsen
Morten Frost Nielsen
Tina Vilsbøll
Filip Krag Knop

Clinical Microbiology Department, Rigshospitalet, University of Copenhagen
Michael Tvede

Novo Nordisk Foundation Center for Basic Metabolic Research, University of Copenhagen
Torben Hansen
Oluf Borbye Pedersen

Gastro Unit, Surgical Section, Herlev Hospital, University of Copenhagen
Jacob Rosenberg

Biomedical Sciences, Panum Institute, University of Copenhagen
Jens Juul Holst

**INTRODUCTION**

Type 2 diabetes mellitus (T2DM) and osteoporosis are common diseases caused by, among other genetic predisposition and inappropriate lifestyle (1,2,3,4). Recent studies suggest that the intestinal bacterial flora may be important in these diseases. Thus, changes in the intestinal bacterial quantity and quality associated with the incidence of type 2 diabetes and osteoporosis. To date, no study investigated the effect of modulating the bacterial flora on the glucose and bone metabolism in humans.


**PURPOSE**

We want to identify whether eradication of the gut microbiota in healthy young men affects the secretion of gut hormones involved in a number of physiological mechanisms (including incretin hormons impact on appetite, satiety, the endocrine pancreatic function and glucose metabolism), bone turnover and inflammation markers. Specifically, we want to examine the effect of that eradicate the normally occurring intestinal bacterial flora using 4-day broad-spectrum antibiotic therapy in healthy young men. Hopefully the results of this study contribute to an understanding of the newly discovered link between intestinal bacterial flora and respectively metabolic diseases and osteoporosis.


**BACKGROUND**

Type 2 diabetes and osteoporosis are common diseases with multifactorial etiologies including genetic predisposition and lifestyle (1,2,3,4). In spite of the clinical diversity of diseases, it appears that the bone and acts on the sugar reacting hinanden5, and recently, it has been pointed out that the intestinal gut flora may play an important role in both of these diseases.

The human intestinal bacterial flora contributes to important metabolic functions, including the ability to absorb nutrients from otherwise indigestible nutrient (6). From stool samples from twins it is suggested that there is a core functionality of the intestinal bacterial flora, but that each subject has its own unique combination of microbes. Conversely, Arumugam et al. suggested that the human gut flora of the individual, across continents, is stratified to one of three "enterotypes" each with its own specialized metabolism and artssammensætning (8). Yet it is not known how these enterotypes correlate with disease occurrence or metabolism. Recently, it was shown that enterotypes correlates to dietary levels of, respectively, carbohydrates and fats / protein (9), and a Danish study has shown that the intestinal bacterial composition in the quality and quantity is correlated to the incidence of type 2 diabetes, as well as plasma glucose levels in a group of adult mennesker10.

Preliminary data from Vrieze (11) has shown that transplantation of feces from the subjects diet for overweight with the metabolic syndrome improved insulin sensitivity and the latter comparison with autologous fecaltransplantation in the same group. Further found Gordon (12) that rodents raised in germ-free conditions developed decreased body fat despite increased energy intake compared with rodents grew up under normal conditions. Transplantation of feces from the normally spawned the sterile spawned mice were also associated with weight gain and increased insulin resistance in the latter. Eradication of the intestinal bacterial flora in obese, insulin resistant rodents with oral antibiotics are similarly been shown to improve these animals fast blood glucose and the results of oral glucose tolerance test (OGTT) (13).

Composition of the diet appears to affect the intestinal bacterial composition in rodents independently of their weight / disease status: after a high-fat diet has been found a corresponding change in intestinal flora composition between the GM 'weight gain resistant' mice and wild type mice, despite the fact that there is only observed weight gain in the latter group (14).

How the intestinal bacterial flora mediate the above effects are not clear, but the following physiological variables have been found effects of a modulated tarmbakterieflora15:

• Concentration and secretion of locally and systemically acting gut peptides and hormones
• Concentration and secretion of bile acids
• The production of short chain fatty acids in the intestinal lumen
• Production of lipopolysaccharide
• The production of cytokines

Glucose-dependent insulinotropic polypeptide (GIP) and glucagon-like peptide-1 (GLP-1) is intestinal hormones which potentiate glucose-stimulated insulin secretion, and are responsible for approximately 70% of the insulin secretion after oral administration of glukose16. Thus, any factor that reduces the GIP and GLP-1 secretion, enhance the post-prandial plasma glucose levels. It has been previously shown that rats fed prebiotics (non-digestible but fermentable foods that promote the growth and / or activity of one or more gut microbes with beneficial effects on the human host (17) develop higher postprandial GIP and GLP-1 responses (18). One might therefore imagine that a modified intestinal flora would affect glucose metabolism.

Rodents raised in germ-free conditions are shown to have a 3-fold increase in the bile acid concentration in the bile, as well as a 25% increase in cholesterol absorption compared to naturally raised animals (19). At the same time a 3-day ampicillin treatment shown to increase bile acid secretion by a factor of 3, and reduce the fecal bile acid loss by 70% in mice (20). Since bile acids have recently been shown to influence the glucose metabolism (through the action of the G-protein coupled receptor TGR5 the GLP-1 secreting L cells in the intestinal mucosa (21) indicates the above finding a previously unrecognized interaction between bacterial flora bile acid secretion and glucose metabolism.

Compared with osteoporosis is that over the recent years also found interesting couplings for the intestinal flora and the secretion of intestinal hormones. In a recent study it was found that changes in the gut flora of mice affects essential bone qualities such as density and structure (22). The authors found a significantly increased bone density correlated to a lower serum serotonin levels in mice that grew up under sterile vs. ordinary circumstances. However, it was not possible from the attempt to clarify whether this finding was a result of increased bone formation and decreased bone resorption.

Abrams et al. found in a human study from 2005 that supplementation with prebiotics vs. control diet resulted in a significantly increased bone mass and density and an increase in calcium intake in a group of adolescents (23). Rodent studies have confirmed this fund (17).

In view of the previously described associations between hormone and intestinal prebiotics, it is also interesting to note that the same intestinal hormones are found associated with bone turnover. In mice a reduced bone formation and an increase in osteoclast concentration and GIP levels was found in germmice compared to wild-type mice (24). Similarly, it was found cortical osteopenia, increased bone resorption and increased osteoclast activity at the GLP-1 receptor knockout mice compared to wild-type mouse (25).

Oral antibiotic treatments previously used prophylactically to eradicate intestinal bacteria prior to colon surgery (26), at intensive care (27), cirrhotic patients with hepatic encefalopati (28) and neutropenic patients with hematological diseases (29). While the efficacy of these regimens has been documented with regard to the risk of infection and mortality, the effect on bone turnover and sugar so far to be elucidated. In a recent study of patients eradicated helicobacter pylori with amoxicillin and clarithromycin was found that the eradicated patients seven months after, eradication had a significantly higher body mass index (BMI) and significantly higher postprandial plasmaleptin- and -ghrelinresponser than before eradikation (30).

Thus, an increasing number of studies have found that the intestinal bacterial flora is important for glucose and bone metabolism. However, it is not clear how the involved effects are mediated. It also seems eradication of intestinal flora that could have opposing effects on glucose metabolism - possibly depending on the intestinal flora composition prior exists in the individual.

In a time of increasing consumption of antibiotika31 and increasing incidence of T2DM32 and osteoporose33 it seems appropriate to examine whether there is a correlation between the previously examined phenomena and changes in tarmbakteriefloraen, which is the aim of the present study.


**Endpoints**

In this study, we will examine whether eradication of intestinal bacteria in humans causes changes in glucose and bone metabolism. We will also examine the composition of bacteria in the feces, blood and saliva before and after intervention (eradication - see below), as assessed by both the cultivation of bacteria and deep metagenomic next-generation sequencing of bacterial DNA. Using bioinformatic analyzes to examine the functional implications of the changes in bacterial composition at the species and phylumniveau have locally in the gut / blood / mouth and on the whole body. Finally, we illustrate the effect of tarmbakterieeradikation of gene activity in the intestinal mucosa and saliva and urine compounding of inflammatory markers before and after the intervention. At participants who - in addition to consent to participate in the main protocol - consents to undergo gastroscopy the effect of the intervention on the composition of bacteria in duodenal sekret and gene expression in duodenal mucosa be assessed. Study end-points in relation to the specific purpose are set out below:

• The effect of microbiota eradication on the secretion of intestinal and pancreashormoner examined by postprandial plasma concentration measurements of the incretin hormones GIP and GLP-1, CCK, gastrin, ghrelin, PYY, GLP-2, oxyntomodulin, insulin, C-peptide, and glucagon before and after the 4 -dags eradication therapy (see below)

• The effect of microbiota eradication on metabolic variables in plasma and urine is assessed by analysis of metabolomics, respectively, from the solid blood plasma, and urine collected before and after 4-day eradication therapy (see below)

• The effect of microbiota eradication on RMR assessed by indirect calorimetry carried out before and after the 4-day eradication therapy (see below)

• The effect of microbiota eradication on body weight, appetite, satiety and food intake will be evaluated by weight registration, standardized questionnaires (on appetite and satiety before, during and after meal test (visual analogue scale (VAS)) and diet registration) and registration of Ingested amount of food related with an ad libitum meal before and after 4-day eradication therapy (see below)

• The effect of microbiota eradication on bone turnover is investigated by means of the plasma concentration measurement of markers of bone formation (osteocalcin, P1NP) and resorption (CTX, 1CTP, sklerostin) and serotonin before and after the 4-day eradication therapy (see below)

• The effect of microbiota eradication on bacterial composition of feces, saliva and blood assessed by both bacterial cultures as deep metagenomic next-generation sequencing of feces, saliva and blood (collected before and after the intervention described below); among participants who agree to gastroduodenoscopy, the effect of the intervention on bacterial composition in duodenal secretion be assessed in a similar manner

• The effect of microbiota eradication of systemic inflammation is assessed by measuring the following serum / plasma markers of inflammation (solid blood samples): high sensitive CRP, lipopolysaccharide binding protein (LPBP), fibrinogen, TNF, IL-6, PAI-1, leptin and adiponectin

• The effect of microbiota eradication on the secretion of bile before and after microbiota eradication assessed by ultrasound investigation of the postprandial gall bladder emptying and measuring the concentration of the various bile acids in the blood before and after 4-day eradication therapy (see below)

• The effect of microbiota eradication on gene expression in duodenal mucosa assessed by exploratory and hypothesis-generating expression analysis (analysis of candidate genes and microarray analysis) performed on duodenal mucosa biopsies taken during standard gastroscopy before and after the intervention described below


**METHODS AND DESIGN**
Attempts Participants
12 healthy young men recruited through advertisements placed in Gentofte Hospital and advertising (including on www.forsoegsperson.dk).

**Inclusion criteria**• Male, age 18-40 years
• Danish caucasian ethnicity
• The ability to give informed consent
• Normal HbA1c (<6%) and / or normal glucose tolerance assessed by 75-g oral glucose tolerance test
• Normal fasting plasma glucose (<6 mM)
• Fasting serum lipids within normal range
• Normal thyroid function
• Eating commonplace and varied Danish diet
• Are non-smoking
• Normal bowel habits, i.e., 1-3 times a day

**Exclusion criteria**• Diabetes, elevated fasting plasma glucose and / or impaired glucose tolerance
• Known bone disorder
• Liver disease (ALT or AST> 2 times the ULN)
• Renal insufficiency (serum creatinine> 130 uM)
• Anemia
• BMI <18.5 kg / m2 or BMI> 25 kg / m2
• Known gastrointestinal disease (including former bariatric surgery, lactose intolerance, celiac disease, inflammatory bowel disease) or family history of inflammatory bowel disease, lactose intolerance or celiac disease.
• Antibiotic therapy within 6 months prior to the study (including malaria)
• any medical treatment that can not be interrupted during the test
• Contraindications against / allergy to the antibiotics used (incl. Previous allergic reaction in connection with the administration of beta-lactam antibiotics, aminoglycosides or vancomycin)
• Contraindications against / allergy to the used sedative used in gastroscopy (propofol) and allergy to soy, eggs or peanuts (cross-sensitivity with propofol); This exclusion criterion applies only to participants who have expressed a wish to undergo gastroscopy

**Experimental design**The study extends over 6 months and involves the individual participates in all six appearances (8 appearances for participants who agree to gastroscopy) and a 4-day course of antibiotics. Persons who by written and oral information accepting participation in the investigation and confirms this in writing, through the following program (shown in Figure 1):

**<6 weeks prior to the test: screening visit**Day 0 Meetings fasting for standardized germ meal test preceded the handover of the bowel, blood, urine and saliva sample (stool sample collected at home under standardized conditions prior to the survey)
Day 0-3 Gut microbiota eradication: Immediately after the meal challenge starts 4-day course of antibiotics (see below); then the participants an ordinary life incl. usual diet in the period (trial participants during this period in telephone contact with the chief doctor at least one time a day)
Day 4: Meetings fasting for standardized germ meal test preceded the handover of the bowel, blood, urine and saliva sample (stool sample collected at home immediately before the survey)
Day 8: Meetings fasting blood samples and submission of stool, blood urine and saliva sample (stool sample collected at home immediately before the turnout)
Day 42: Meetings fasting for standardized germ meal test preceded the handover of the bowel, blood, urine and saliva sample (stool sample collected at home immediately before the survey)
Day 180: Meetings fasting blood samples and submission of stool, blood, urine and saliva sample (stool sample collected at home immediately before the turnout)

At participants who - in addition to consent to participate in the main protocol - consents to undergo gastroscopy (with the collection of biopsies and duodenal secretions), these will be carried out partly in an autonomous day before the intervention start (from after inclusion until day -1) and on day 3 (immediately before the last dosing antibiotics). In both the meetings, the participants must be fasting.

**Screening**Participants meet in the laboratory after 10 hours of fasting. Here, measured and weighed participants, medication and medical history recorded measured blood pressure and taken screening blood tests (creatinine, electrolytes (Na + and K +), TSH, ALT, AST, alkaline phosphatase, hemoglobin HbA1c and fasting plasma glucose and fasting lipid profile). Take for screening blood samples about 10 ml of blood. If the participants based on results from the screening meeting may participate in the project, agreed dates for study days. If the biochemical tests are abnormal, participants will be advised as to further investigation and studies if desired.

In all of the following 5 visits through the study participants a brief medical assessment when the heart rate, blood pressure and weight recorded.

**The test meal (Day 0, Day 4 and Day 42)**The subject meetings in the laboratory at 8 am after 10 hours of fasting / thirst (incl. Coffee, tea and any. Medication). Brought a peripheral venflon (for blood sampling) in the vein on the back of the hand, which is kept warm (approximately 50 ° C) - to the venous blood arterialize - using heat lamp throughout the experiment. Before baseline emptied the urinary bladder and 10 ml is taken from the (distributed into two tubes and frozen at -20 ° C until later analysis). The subject must then and throughout the trial will be seated. 30 minutes before the meal (time -30 min) initiated indirect calorimetry measurement of respiratory gas exchange to steady-state over 30 minutes using calorimeter (CCM Express, Indirect Calorimeter, MedGraphics). At time 0 min to start taking a liquid sterilized meal over 10 minutes (time 0-10 min) of 200 ml Nutridrink with cocoa flavor (Nutricia, Allerød, Denmark) (300 kcal, 55 g carbohydrate, 17 g fat and 18 g protein ) were added 1.5 g of paracetamol (effervescent tablets) dissolved in 50 ml of sterile water for a total volume of 250 ml (under sterile conditions Take 1 ml of sample from the control culture and sequencing to ensure the sterility of the meal). Blood samples are taken from the peripheral venous cannula at times -30, -15, 0, 15, 30, 45, 60, 75, 90, 105, 120, 150, 180, 210 and 240 minutes. Blood sample is taken at each 0.2 ml of blood in natriumflouridrør for determination of plasma glucose, 12 ml blood in EDTA tubes for the determination of plasma concentrations of GLP-1, GIP, CCK, gastrin, ghrelin, PYY, GLP-2, glucagon and oxyntomodulin, and 2 mL of dry vial for determination of serum concentrations of insulin and C-peptide as well as paracetamol (for estimating gastric emptying time). At time 0 min withdrawn addition, some 40 ml of blood for the determination of inflammation and bone markers as well as glass for RNA assay. Blood / plasma / serum are stored at -20 / -80 ° C until analysis. A total are not more than 300 ml of blood. Gall bladder volume is measured by ultrasonography at times 0, 30 and 60 min. At times 0, 30, 60, 90, 120, 150, 180, 210 and 240 minutes assessed subjects 'appetite (hunger), fullness (satiety), filling (fullness), and prospective food consumption (prospective food consumption) by means of standardized VAS' is. At time 60 minutes made again colorimetric measurement over 30 minutes. At time 240 min Total urine was collected for analysis for the presence of glucose and nitrogen is taken from and10 ml (divided into two tubes and frozen at -20 ° C until later analysis). Then ingested a standardized ad libitum meal consisting of ground beef, pasta, corn, carrots, peppers and cream and salt and pepper (50 energy (E)% carbohydrate, 37 E% fat, 13% protein E). The subjects were instructed to eat as much as they can, until they feel comfortable saturate. The meal is consumed in the course of a maximum of 30 minutes. At the end of the ad libitum meal recorded time and the weight and the amount of energy of the ingested ad libitum meal. The participants also evaluate the meal taste, smell, visual appeal, aftertaste and overall palatability (palatability) when they are finished with the meal, using standardized VAS'er. Immediately after the meal ad libitum on day 0 ingested the first dose of eradikationskuren (see below). After ad libitum meal on day 42 taken blood sample for measurement of hemoglobin and based on the results of this test instituted if necessary. general, oral iron therapy for 14 days and measured again after the hemoglobin further 14 days.

Registration of diet composition and volume (day 0, day 4, day 8 and day 42)
At one point during each meal test on days 0, 4 and 42 (as well as by attendance at day 8) completed validated food frequency questionnaire regarding participants' diet composition and quantity in the four previous days (the form is attached as Appendix 6).

**Antibiotic (day 0-day 3)**After the first meal test is started up the eradication of the intestinal bacterial flora with 4-day-lasting 3-substance antibiotic consisting of 500 mg of vancomycin (powder for solution for infusion), 500 mg of meropenem (powder for solution for infusion) and 40 mg of gentamicin (powder for solution for infusion) dissolved in 1 glass (200ml) fruit juice. The solution is given per orally one time a day for 4 consecutive days. The first dose should be taken immediately after the meal challenge on day 0, while the subject is still in the hospital. Second, third and fourth dose dispensed (a total of nine vials) to the subjects on day 0 and must be consumed dissolved in 200 ml of juice in each of the following 3 days in the evening. Participants are encouraged not to change their normal food intake during the study. Sponsor physician contacts trial participants per phone 1 once daily on days 1, 2 and 3 in order to ensure proper medication intake and eliminate signs of drug side effects.

Fecal samples (day 0, day 4, day 8, day 42 and day 180)
Stool samples used for determining the bacterial count and -subtyper by means of two methods: sequencing of bacterial DNA, and cultivation of intestinal bacteria. Participants will receive collection set consisting of cooler bag with ice packs, packaging and stools glass. Fecal samples collected in subjects' homes close to the time of delivery as possible. The exact treatment of stool samples is different for the two assay methods.

**Fecal collection for DNA sequencing**Prior to defecation placed a one collects in the toilet where feces transferred to two glasses who immediately transferred to a transport container and placed at -20 ° C in his own cabinet. Prior to transport to Gentofte Hospital transferred transporting container to the cooler and on arrival at the hospital samples are transferred immediately to -80 ° C freezer, where the samples are stored in a biobank for later analysis.

**Fecal collection for bacteria cultivation**Feces collected as above to transport container and then placed in a private refrigerator. Prior to transport to Gentofte Hospital transferred transporting container to the cooler and on arrival at the hospital sent the sample to study at the Clinical Microbiology Department, University Hospital.

Apart from stool samples for microbiological examination are collected prior to antibiotics cure a stool sample for the treatment of any antibiotic-associated severe and prolonged diarrhea. This sample is handled in the same way as the sample for the bacterial culture described above. The test may by the physicians and the consent of the subject to be re-same subjects on rectal probe if there might be an indication that purpose (see "Side effects, risks and benefits").

In connection with the collection of stool sample at the end of antibiotics cure (day 3) is collected also an additional stool sample (taken and treated similar to the samples for DNA sequencing (see above)) to determine fecal vancomycin concentration in order to assess the pharmacokinetic aspects of antibiotics cure . The provision takes place at the Clinical Microbiology Department, University Hospital.

**Blood tests (day 0, day 4, day 8, day 42 and day 180)**Blood test for purification of bacterial DNA taken to hospital in real mode prior to ad libitum meal. Blood collection and storage are as for the purpose of human DNA purifikation.

**Saliva samples (day 0, day 4, day 8, day 42 and day 180)**Sputum samples are collected in the hospital (on day 0, day 4 and day 42 of the minute immediately before the meal ad libitum). The following procedure is used: The subject takes a piece of paraffin in your mouth and chew at a pace that seems natural for him until the paraffin has become a coherent piece (about one minute). The subject sinking the saliva that may be produced in one minute. The clock is started and then the resultant saliva was collected over the next three minutes by the subject spits out in a beaker. Saliva samples were distributed into two tubes of 1.8 ml and 4.5 ml. The small pipes freeze directly on dry ice as soon as possible, and then stored at -80 ° C. The second tube RNAlater is added in the ratio 1: 3 (saliva: RNAlater) and placed in a refrigerator for about 24 hours, after which it is frozen to -80 ° C until analysis.
For the sake of bacterial identification shall attempt the participants not to brush your teeth in the morning before sputum sample collection.

Urine samples (day 0, day 4, day 8, day 42 and day 180)
Urine from the mid-stream of the second urination of the day is collected in a sterile container at the laboratory in the morning. The sample is frozen and stored at -80 ° C.

**Optional gastroscopy (day -1 (or before) and day 3)**Performed in Endoskopienheden at Gentofte Hospital after 8 hours of fasting. The study carried out by a trained doctor after the department's standard instructions. The examination lasts about 15 minutes, but expected another 5 minutes prior sedation see. Script. During the examination is amended as follows: There is collected (aspirated) - if possible - secretions to determine bacterial presence evaluated by sequencing of bacterial DNA and cultivation. Take further 11 standard mucosal (about 3-5 mg) - 6 to gene expression and 5 to immunohistochemistry - from duodenums second paragraph. After the investigation reinstructed participant in that he should not be driving a motor vehicle for the day. Gastroscopy performed as indicated only on participants who have given specific consent for this study. In cases of moderate / severe antibiotic-associated adverse events on day 3 (the second gastroscopy is scheduled), the study will be canceled. Similarly, the study will be canceled in any case of general effects or discomfort in the participant.


**RATIONALE BEHIND THE STUDY DESIGN**
There is a physiological intervention study in which participants are their own controls. The effect of eradication of the natural intestinal bacterial flora evaluated in efficacy variable listed in the "Endpoints" above. As the basic knowledge in the field is extremely scarce, we want in the first place to look at conditions in healthy young men without diseases that theoretically could influence the outcome (see exclusion criteria). As a measure of the actual changes in the bacterial flora, stool samples, duodenal biopsies and saliva samples will be analyzed. The used antibiotic 'cocktail 'is a tool to achieve microbiota eradication and is not expected to have therapeutic effects or significant effects on the body as a whole.


**CALCULATIONS AND STATISTICS**
The primary endpoint (postprandial plasma GLP-1 and -GIP responses) assessed as area under the curve (AUC) for plasma GLP-1 and -GIP values ​​after meal ingestion. The same applies to the other postprandial hormonresponser in plasma (secondary endpoints). Changes in other plasma / serum markers including bone and inflammation markers is evaluated by comparing the real values ​​before and after the eradication of the intestinal flora.

Ejection fraction, ejection fraction (EF), from the gall bladder can be determined using the following formula:


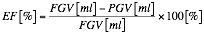


FGV = fixed gallbladder volume; PGV = postprandial gallbladder volume; EF = ejection fraction.

Data will be processed and presented using standard descriptive statistics. Comparison of normal data is performed using unpaired two-tailed t-tests. Data that are not normally distributed are compared by the Mann-Whitney U-test. Two-factor repeated measurement analysis of variance is used for statistical analysis of the repeated measures from the same trial participant.

By including 10 people in the study, we will be capable of detecting a minimum relevant difference of 10% for the primary endpoint (change in AUCGLP-1as tarmbakterieeradikation), based on the standard deviation of postprandial GLP-1 responses (assessed by AUC GLP-1), with: a strength of 85% and a significance level of 5%. To ensure the mentioned strength and increase the chances of achieving relevant and significant findings with regard to the secondary endpoints included 12 participants in the study.


**PRACTICAL MEASURES**
Screening and clinical experimental procedures (disregarding gastroduodenoscopy) will take place in Diabetes Research Unit, Medical Unit F, Gentofte Hospital, where appropriate equipment and expertise available. Gastroscopy made in Endoskopienheden at Gentofte Hospital, which annually performed thousands of these studies. Plasma Concentration Measurements of incretin hormones GIP and GLP-1, CCK, gastrin, ghrelin, PYY, GLP-2, oxyntomodulin and glucagon will be taken by Professor Jens Juul Holst's laboratory at the Panum (Biomedical Sciences, Health Sciences, University of Copenhagen), who have decades experience in these analyzes. The lab will also assist with exploratory and hypothesis-generating expression analysis of tissues taken during gastroscopy (analysis of candidate genes and microarray analysis). Serum insulin and-C-peptide measured at Department of Clinical Biochemistry, Gentofte Hospital. Serum Bile acids determined with the help of the Department of Clinical Biochemistry, Roskilde Hospital. Cultivation of bacterial samples made at the Clinical Microbiology Department, University Hospital. Section of Metabolic Genetics, Novo Nordisk Foundation Center for Basic Metabolic Research, University of Copenhagen, is responsible for metabolomics analysis, DNA purifikation from saliva, blood and bowel / stool and deep metagenomic next-generation sequencing of bacterial DNA and measurement of serum - / plasma markers of inflammation.


**PROJECT POSSIBLE ROLE**
The study will provide important information about the interaction between the intestinal bacterial flora and glucose and bone turnover. If we find that the intestinal flora is essential for bone and / or glucose metabolism, it will be natural to imagine new treatment principles in frequent diseases such as type 2 diabetes and osteoporosis, based on modeling of the intestinal flora.


**SIDE EFFECTS, RISKS AND DISADVANTAGES OF TRIAL PARTICIPANTS**
The main drawback for the individual trial participant, the risk of developing dehydration associated with diarrhea in addition to the antibiotic regimen. Symptoms of dehydration will be thoroughly examined with the participants, and all participants will be instructed to consume plenty of fluids if antibiotics induced diarrhea occurs. If signs of dehydration should this occur, the test subject contact the study investigator, who will decide whether the subject should discontinue antibiotics cure or continue in the study. In the event of prolonged diarrhea (after completion of antibiotics cure), it is possible that the subject may receive autologous fæcestransplantation using fecal sample collected prior to antibiotic regimen. This treatment is well known and is typically used for prolonged treatment resistant cases of diarrhea, for example gastroenteritis and can contribute to a faster recovery of the natural bakterieflora34. The three types of antibiotics used in this study, is recorded only in a very limited extent tarmvæggen35 why the risks of systemic effects, side effects and the development of allergies is believed to be small. Exanthema and urticaria have been described in between 0.1 and 1% of patients receiving intravenous or Meropenem Gentamicin in clinical practice (equivalent 1 to 10% by intravenous vancomycin treatment) 35th In a brief per oral antibiotic supposed these risks to be much lower.
In any case of emerging symptoms during the trial or immediately after the trial the subject will contact the study investigator, who will decide on further level. Signs of allergic pharmacological response will cause acute medical assessment and treatment. The further investigation for possible allergy and guidance on this will be done in consultation with the allergy clinic at Gentofte Hospital. As a consequence of proven allergy to the subject in future cases of bacterial infection being withheld possibility of receiving the antibiotics. This is considered as a rare side effect (estimated below 0.1% risk) for participation in the trial.

Gastroscopy for the subject associated with minimal discomfort, since the study is performed intoxication with propofol. Theoretically, at any endoscopy in which the sedation with propofol is used, a risk of drug side effects, pulmonary problems (aspiration pneumonia and hypoxia), cardiac arrhythmia, as well as perforation of the wall of the gastrointestinal tract, and lesions blødning36 instrument. Traditionally indicate a complication risk of 1 per thousand and a mortality rate of 1 in 10,000 for diagnostic gastroduodenoskopi36. These complication and death rates can not be transferred directly to our study, when we do the study on healthy individuals. In fact, the risk of complications of the studies thus be considered as minimal. The study is canceled as indicated in cases of moderate / severe antibiotic associated side effects and in any case of malaise in the test participant.

As a theoretical complication for the construction of venous catheters (and any penetration of the skin and blood vessels with sharp / pointed objects) should be mentioned superficial phlebitis (phlebitis). The condition is harmless. The risk of superficial phlebitis is small and minimized by following clinical standards for bringing venous catheters including double wiping it involved skin with disinfectant alcohol and other sterile procedures.

The blood loss by participation is a maximum of 300 ml per minute. test day and thus total less than 900 ml of blood in three compilations over 6 weeks. In healthy young men, this is not associated with risk in itself. The selection may lead to fatigue immediately after the examination. After the third meal test offered participants iron therapy with ordinary iron tablets.


**EXPERIMENTAL PART'S PHYSICAL AND MENTAL INTEGRITY AND PRIVACY**
Information concerning the individual trial participant protected under the Act on the processing of personal data and the Health Act.


**Biobank**
The biological material assigned by the investigator a code corresponding to the individual trial subject and time of sampling. The material is stored in secured and locked conditions until analysis in a research biobank. The plasma / serum samples are expected to be finalized within a few months after the experimental procedures, while other blood tests (including human leukocyte DNA) and samples to metabolomic analyzes and urine, saliva and stool samples expected to be analyzed within 1-5 years. Extras will be stored for up to 15 years after the end of the trial in order to repeat the error analysis and the possible need for further analysis. The use of these tests for another project will require a re-approval of a Research Ethics Committee of the Capital Region. After 15 years, any remaining biological material will be destroyed along with any personally identifiable data and code can be identified. Parts of intestinal contents / faeces and saliva samples will probably by the research group will be sent to a foreign laboratory for sequencing of bacterial DNA. Other samples will be analyzed in Denmark. The project and the biobank notified to the DPA.


**RESEARCH GROUP**
From Diabetes Research Unit, Medical Unit F, Gentofte Hospital Kristian Hallundbæk Mikkelsen, Tina Vilsbøll, Morten Frost Nielsen, Filip Krag Knob. From the Clinical Microbiology Department, University Hospital, Michael Tvede. From the Section of Metabolic Genetics, Novo Nordisk Foundation Center for Basic Metabolic Research, University of Copenhagen, Professor Torben Hansen and Oluf Borbye Pedersen. From Surgical Section, Herlev Hospital Jacob Rosenberg and from Biomedical Sciences, Panum Institute, University of Copenhagen Jens Juul Holst.


**OPERATING AND FINANCIAL MATTERS**
The project is initiated by the first, PhD Filip Krag Knop, Diabetes Research Unit, Medical Unit F, Gentofte Hospital. Neither Filip Krag Knop or other research group behind the project has economic interests in the performance or the results of the project. Operating expenses for utensils, screening blood tests and laboratory technician using covered by the Medical Unit F, Gentofte Hospital. The project's other operating expenses envisaged covered through private and public funds that will be regularly applied. The fund will be deposited into a fund account associated with the project under the Medical Unit F, Gentofte Hospital, which is under the hospital's audit. At present, the project has received support from the Medical Unit F, Gentofte Hospital in the form of two months' salary to the chief doctor, Kristian Hallundbæk Mikkelsen, for initiating the project. In addition, the project has received financial support. None of the project has financial ties to private companies, foundations, etc., which could have an interest in the research project. Information received support (name of support allows, aid including the method of payment) will be sent by the Research Ethics Committee of the Capital Region with the aim of ethical consideration and approval.


**REMUNERATION AND COST REIMBURSEMENT FOR TRIAL PARTICIPANTS**
There shall cover documented transport costs which participants may have in connection with the participation. Due to the project's time scale for each participant comes drawback allowance of kr. 4,000 (taxed as B-income) for each participant as well as an additional allowance of kr. 1,000 (taxed as B-income) to those of the subjects in addition to the main protocol also undergo gastroduodenoscopy before and after eradication therapy. Disadvantage allowance shall be paid from the project fund account to the participant's NEM account once the investigation is completed for each participant. If a trial participant chooses to step out of the trial until this is completed, the ulempegodtgørelsens size to the current session time.


**RECRUITMENT OF PARTICIPANTS**
Participants recruited through advertisements placed in Gentofte Hospital and advertising (including on www.forsoegsperson.dk). See also the section entitled "Guidelines for the submission of oral information and obtaining consent". For the above purpose, the enclosed ad text will be used.


**AVAILABILITY OF INFORMATION FOR VOLUNTEERS**
The participants are guaranteed access to more information about the project. Contact: doctor Kristian Hallundbæk Mikkelsen, diabetology Research Unit, Medical Unit F, Gentofte Hospital; tel .: 61 69 97 59; e-mail: kristianmikkel@gmail.com.


**PUBLICATION OF TEST RESULTS**
Data expected to be published in international scientific journals. Both negative and positive test results will be published. Act on processing of personal data will be respected.


**INTERRUPTION OF TRIAL**
The trial is interrupted for each participant in case he wishes to be deleted from the current Protocol, or in case of exceptional circumstances make it impossible to complete the trial. Likewise, extraordinary events causing the project can not be complete in its entirety, cause withdrawal for all ongoing trials participants.


**SAFETY**
Gastroscopies are performed by standard instructions of doctors with extensive experience of the procedures. The subjects will the procedure be easily sedated by trained anesthesia nurses to quickly call by anesthesiologists in case of unexpected effects or complications. Insertions of peripheral venous catheters will be performed by doctors with extensive experience on procedures and sterile technique. The trial takes place in Diabetes Research Unit, Medical Unit F, Gentofte Hospital, as well as in Endoskopienheden, Gentofte Hospital. Before inclusion is the study participants found suitable for participation by medical examination. Subjects covered by the general patient insurance. Possible side effects associated with antibiotics cure will be announced chief doctor, who will then decide on plan.


**SCIENCE ETHICAL REVIEW**
All participants will receive oral and written information, and there will be oral and written consent to participate in the studies. Participants are informed by a doctor who does not perform any treatment of participants in the daily. The protocol complies with Helsinki Declaration II.

There are as listed risk for diarrhea / loose stools in addition to the antibiotic regimen. This disadvantage is minimized by making antibiotics cure short and also carry it out on healthy young men. Diarrhea can result in dehydration. Symptoms of dehydration will be thoroughly examined with the participants, and all participants will be instructed to consume plenty of fluids if antibiotics induced diarrhea occurs. On suspicion of serious side effects from antibiotics cure will test subject immediately receive appropriate medical treatment and assessment. The modest risk of developing allergy to the applied three antibiotics previously described, possible allergic reactions will be handled in cooperation with the allergy clinic.

Gastroscopies with biopsies are associated with minimal discomfort since the survey conducted in intoxication with propofol. There is, as stated above (see 'Side effects, risks and benefits for the study participants') a very limited risk of complications associated with the investigation.

The individual trial days (3 meal test) is associated with minimal discomfort to the subjects. The only drawback of participation are the connectors at the construction of peripheral venous needle for blood collection. As a theoretical complication for the construction of peripheral venous needle (and any penetration of the skin and blood vessels with sharp / pointed objects) should be mentioned superficial phlebitis (phlebitis). The condition is harmless and self-limiting. Superficial phlebitis bacterial origin can be treated with antibiotics. The risk of superficial phlebitis is small and minimized by following clinical standards for the construction of peripheral venous needle and blood sampling included double wiping it involved skin with disinfectant alcohol and other sterile procedures.

The total blood loss in six weeks is less than 900 ml for each participant. Only participants with normal hemoglobin can participate. Participant offered iron treatment after the trial.

All participants assigned a trial number and will datasheet and blood collection tube only appear with initials and attempt number. The full name, social security number and attempt number stored separately. The project is subject to approval by a Research Ethics Committee of the Capital Region enroll Data Protection Agency.

If necessary, the participants are covered by the general patient insurance.

Biological material will be stored in a research and treatment mentioned in the "Biobank".

The project will not benefit the individual participant to good (apart from the regular checkup, which is part of the screening visit), but it will illustrate the interaction between the intestinal bacterial flora and the development of type 2 diabetes and osteoporosis. Knowledge in this area could in the long term lead to an improved understanding of the pathogenesis of these diseases, thereby creating the opportunity for improved treatments. These expected benefits outweigh by the research group's present knowledge of the fact that the project is not likely to get each participant to good (apart from the regular checkup) and the minimal risks and side effects and the modest discomfort for the participants, the project is connected with (mentioned above) .


**GUIDELINES FOR SUBMISSION OF THE ORAL INFORMATION AND OBTAINING CONSENT**
Participants will be recruited through notices or through advertising (in particular www.forsoegsperson.dk). If the subjects responds to lookup takes chief physician contact and clarify whether any attempt person wishing to participate in an informal information interview about the project. Time and place agreed. In addition, information about the right to bring an accompanying person will be given. Before the interview, the written information about the project will be sent, ie participant information (Appendix 2), as well as material "Before you decide" (Annex 8) and "trial rights of persons in a biomedical research" (Annex 7)
For information interview that takes place in serene settings with someone who has the professional qualifications to communicate the contents of the research project and which are directly related to the research project, indicate only that there is a request to participate in a biomedical research project. If people are still interested in participating in the project, agreed time and place for obtaining written consent (the subject and the project responsible physician signing the consent form). Only then made study specific procedures.

##

## REFERENCES

1. Rifkin H, Porte D Jr (Eds). *Ellenberg and Rifkin’s Diabetes Mellitus*. New York: Elsevier; 1990.

2. Nolan, C.J., Damm, P. & Prentki, M. Type 2 diabetes across generations: from pathophysiology to prevention and management. *Lancet* **378**, 169-181 (2011).

3. Pocock NA, Eisman JA, Hopper JL, et al. Genetic determinants of bone mass in adults. A twin study. *J Clin Invest.* 1987;80(3):706-710.

4. Hawker, G.A., Jamal, S.A., Ridout, R. & Chase, C. A clinical prediction rule to identify premenopausal women with low bone mass. *Osteoporos Int* **13**, 400-406 (2002).

5. Clemens TL, Karsenty G. The osteoblast: an insulin target cell controlling glucose homeostasis. *J Bone Miner Res.* 2011;26(4):677-680.

6. Turnbaugh, P.J. *et al.* The human microbiome project. *Nature* **449**, 804-810 (2007).

7. Turnbaugh, P.J. *et al.* A core gut microbiome in obese and lean twins. *Nature* **457**, 480-484 (2009).

8. Arumugam, M. *et al.* Enterotypes of the human gut microbiome. *Nature* **473**, 174-180 (2011).

9. Wu, G.D. *et al.* Linking long-term dietary patterns with gut microbial enterotypes. *Science* **334**, 105-108 (2011).

10. Larsen, N. *et al.* Gut microbiota in human adults with type 2 diabetes differs from non-diabetic adults. *PLoS ONE* **5**, e9085 (2010).

11. Vrieze A, Holleman F, Serlie MJ, et al. Metabolic effects of transplanting gut microbiota from lean donors to subjects with metabolic syndrome. *Diabetologia*. 2010;53:(Suppl1):1–556.

12. Turnbaugh, P.J. *et al.* An obesity-associated gut microbiome with increased capacity for energy harvest. *Nature* **444**, 1027-1031 (2006).

13. Membrez, M. *et al.* Gut microbiota modulation with norfloxacin and ampicillin enhances glucose tolerance in mice. *FASEB J.* **22**, 2416-2426 (2008).

14. Hildebrandt MA, Hoffmann C, Sherrill-Mix SA, et al. High-fat diet determines the composition of the murine gut microbiome independently of obesity. *Gastroenterology*. 2009;137(5):1716-1724.

15. Kootte RS, Vrieze A, Holleman F, et al. The therapeutic potential of manipulating gut microbiota in obesity and type 2 diabetes mellitus. *Diabetes, Obes Metab*. Epub aug. 2011.

16. Nauck MA, Bartels E, Orskov C, et al. Additive insulinotropic effects of exogenous synthetic human gastric inhibitory polypeptide and glucagon-like peptide-1-(7-36) amide infused at near-physiological insulinotropic hormone and glucose concentrations. *J Clin Endocrinol Metab.* 1993;76(4):912-917.

17. Roberfroid M, Gibson GR, Hoyles L, et al. Prebiotic effects: metabolic and health benefits. *Br J Nutr.* 2010;104(Suppl 2):1-63.

18. Cani, P.D. *et al.* Improvement of glucose tolerance and hepatic insulin sensitivity by oligofructose requires a functional glucagon-like peptide 1 receptor. *Diabetes* **55**, 1484-1490 (2006).

19. Wostmann BS. Intestinal bile acids and cholesterol absorption in the germfree rat. *J Nutr.* 1973;103(7):982-990.

20. Miyata M, Yamakawa H, Hamatsu M, et al. Enterobacteria modulate intestinal bile acid transport and homeostasis through apical sodium-dependent bile acid transporter (SLC10A2) expression. *J Pharmacol Exp Ther.* 2011;336(1):188-196.

21. Thomas, C. *et al.* TGR5-mediated bile acid sensing controls glucose homeostasis. *Cell Metab.* **10**, 167-177 (2009).

22. Sjögren K, Engdahl C, Lagerquist M, et al. *Amer Soc Bone Miner Res*. Annual Meeting 2010, oral presentation number 1170.

23. Abrams SA, Griffin IJ, Hawthorne KM, et al. A combination of prebiotic short- and long-chain inulin-type fructans enhances calcium absorption and bone mineralization in young adolescents. *Am J Clin Nutr.* 2005;82(2):471-476.

24. Tsukiyama K, Yamada Y, Yamada C, et al. Gastric inhibitory polypeptide as an endogenous factor promoting new bone formation after food ingestion. *Mol Endocrinol.* 2006;20(7):1644-1651.

25. Yamada, C. *et al.* The murine glucagon-like peptide-1 receptor is essential for control of bone resorption. *Endocrinology* **149**, 574-579 (2008).

26. Nelson, R.L., Glenny, A.M. & Song, F. Antimicrobial prophylaxis for colorectal surgery. *Cochrane Database Syst Rev* CD001181 (2009).doi:10.1002/14651858.CD001181.pub3

27. D’Amico, R. *et al.* Effectiveness of antibiotic prophylaxis in critically ill adult patients: systematic review of randomised controlled trials. *BMJ* **316**, 1275-1285 (1998).

28. Bass NM, Mullen KD, Sanyal A, et al. Rifaximin treatment in hepatic encephalopathy. *N Engl J Med.* 2010;362(12):1071-1081.

29. Buzyn A, Tancrède C, Nitenberg G, Cordonnier C. Reflections on gut decontamination in hematology. *Clin Microbiol Infect.* 1999;5(8):449-456.

30. Francois, F. *et al.* The effect of H. pylori eradication on meal-associated changes in plasma ghrelin and leptin. *BMC Gastroenterol* **11**, 37 (2011).

31. Korsgaard H, Agersø Y. DANMAP 2010 - Use of antimicrobial agents and occurrence of antimicrobial resistance in bacteria from food animals, food and humans in Denmark. Statens Serum Institut 2010.

32. www.sst.dk. Tabel 3 incidens af nye diabetikere i Danmark 1997-2009.

33. Christensen K, Bjørk C, Vinter-Larsen M. Otte folkesygdomme - forekomst og udvikling. Statens institut for folkesundhed 2005.

34. Bartlett JG. Narrative review: the new epidemic of Clostridium difficile-associated enteric disease. *Ann Intern Med* 2006;145(10):758-764.

35. www.pro.medicin.dk lægemiddeloplysninger for de enkelte præparater.

36. Cotton P, Williams C. *Practial gastrointestinal endoscopy*. **1996**, (Blackwell Science: ).

# BILAG 1, LÆGMANDSRESUMÉ

**Eradikation af den humane tarmflora**

*Effekt på postprandial tarmhormonsekretion, glukosemetabolisme, knogleomsætning og tarmmikrobiom*

## BAGGRUND OG FORMÅL

Type 2-Diabetes (T2-DM, tidligere kaldet ”gammelmandssukkersyge”) og knogleskørhed, som også kaldes osteoporose, er hyppigt forekommende sygdomme forårsaget af blandt andet arv og livsstilsfaktorer. Indenfor de senere år er man blevet opmærksom på, at tarmens bakterier kan have betydning for udvikling af disse sygdomme. Det er blevet hævdet, at tarmbakterierne kan indvirke mere eller mindre gavnligt på kroppen afhængigt af sammensætningen af bakterierne.

Undersøgelser har vist, at den menneskelige tarmbakterieflora kan hænge sammen med forekomsten af T2-DM; og ved at ændre sammensætningen af tarmbakterierne hos dyr har man kunnet påvirke dyrenes sukkeromsætning. Også ved knogleskørhed har man sandsynliggjort, at en ændret tarmbakterieflora kan påvirke knogleomsætningen hos både dyr og mennesker. Det er kendt, at udskillelsen af en række hormoner, galdesyrer samt visse fedtsyrer påvirkes, når bakteriefloraen i tarmen ændres, men forklaringen bag disse effekter er aktuelt ukendt.

I dette projekt vil vi derfor undersøge, om en midlertidig fjernelse af tarmbakterierne hos mennesker medfører forandringer i sukker- og knogleomsætningen, ligesom vi vil forsøge at afklare, ad hvilke biokemiske veje disse forandringer i givet fald sker. Som led i dette vil vi undersøge, hvordan bakteriefjernelsen indvirker på:

1. udskillelsen af en række tarm-, og sukkerhormoner, foruden markører for opbygningen og nedbrydningen af knoglerne samt betændelse.

2. udskillelsen af galdesyrer fra galdeblæren.

3. appetitregulering og hvilestofskifte.

4. forekomsten af bakterier og betændelsesmarkører i urin og spyt.

5. aktiviteten i forskellige gener i tolvfingertarmen

Vi vil desuden undersøge sammensætningen af bakterierne, når disse er kommet tilbage til tarmsystemet.

## DESIGN

I undersøgelsen skal indgå 12 raske mænd, af dansk etnicitet, i alderen 18-40 år. For at deltage i undersøgelsen må man ikke have sukkersyge eller nogen kendt knoglesygdom, man skal i det daglige indtage en nogenlunde gennemsnitlig, varieret dansk kost (ikke være vegetar, veganer eller spise efter særlig diæt). Desuden udelukkes man fra undersøgelsen, hvis man fejler noget i nyrer, lever, skjoldbruskkirtel og tarmsystemet eller nære slægtninge med arvelige mavetarmsygdomme, hvis man er ryger eller er i behandling med medicin, der kan påvirke det, vi vil undersøge (f.eks. binyrebarkhormon) eller hvis man er allergisk overfor den anvendte medicin.

Undersøgelsen strækker sig over 6 måneder og indebærer for den enkelte deltager i alt 6 besøg på afdelingen samt en 4 dages antibiotikakur i hjemmet.

Personer, som ønsker at deltage i undersøgelsen og som umiddelbart kan indgå i projektet, skal efter afgivelse af informeret samtykke have foretaget en række blodprøver, som skal afklare, om de opfylder kriterierne for deltagelse.

Hvis disse prøver er normale, inkluderes vedkommende i undersøgelsen, og der vil derefter blive foretaget en række undersøgelser:

Ved forsøgets start indsamles afførings-, urin og spytprøve og der foretages en såkaldt måltidstest (beskrevet nedenfor).

Forsøgsdeltageren indtager derefter over 4 dage antibiotika, der fjerner bakterierne i tarmsystemet, og derefter gentages såvel måltidstesten som indsamlingen af afføring, spyt og urin. En uge efter den første måltidstest indsamles igen afføring, spyt og urin og efter 6 uger gentages måltidstesten sammen med indsamling af afføring, urin og spyt. Ét halvt år efter at projektet er startet, indsamles for sidste gang afføring, urin og spyt.

Hos deltagere, der - udover at samtykke til deltagelse i hovedforsøget - samtykker til at få foretaget kikkertundersøgelser af tolvfingertarmen, vil disse blive udført dels på en selvstændig dag før antibiotikakuren (fra efter inklusion frem til dag -1) og på dag 3 (umiddelbart før sidste antibiotikaindtag). For disse deltagere er der således tale om 8 (og ikke 6) besøg på afdelingen.

En oversigt over forsøget er vist herunder:

-14 -1 0 1 2 3 4 8 42 180 dage

Screening

antibiotikakur

MT + OPS

MT + OPS

MT + OPS

OPS

OPS

MT = Måltidstest og ad libitum-måltid som beskrevet

OPS = Opsamling af afførings-, spyt- og urinprøver

Evt. gas = Udførelse af gastroduodenoskopi hos de deltagere som giver samtykke hertil

evt. gas

evt. gas

### Undersøgelser og procedurer

På dagen for måltidstesten møder forsøgsdeltageren i laboratoriet klokken 8 efter 10 timers faste. Inden forsøgsstart opsamles en urinprøve og der anlægges en venekanyle (dropnål) i en vene (et tyndt blodkar) i hånden eller underarmen. Herfra tages ca. 40 ml blod til bestemmelse af en række genparametre, betændelsesmarkører samt knoglemarkører i blodet. Der foretages derefter en måling af hvilestofskifte ved at opsamle forsøgsdeltagerens udåndingsluft i en særlig maske. Herefter bestemmes galdeblærens størrelse ved hjælp af en ultralydsscanning og det egentlige forsøg startes: Forsøgspersonen indtager 200 ml Nutridrink (ernæringsdrik med kakaosmag) samt 1,5 gram paracetamol (3 styk almindelige håndkøbssmertestillende tabletter) over ca. 10 minutter. Over de næste 4 timer opsamles løbende en række blodprøver via den anlagte venekanyle ligesom gasmåling på udåndingsluft og ultralydsscanning af galdeblæren gentages nogle gange. Desuden vil forsøgsdeltageren i løbet af de 4 timer blive stillet en række spørgsmål vedrørende appetit og mæthedsfornemmelse.

Blodprøverne skal vise, om sammensætningen af markører for sukker- og knogleomsætningen ændres, ligesom der også måles på udskillelsen af en række tarmhormoner. I alt tages i forbindelse med måltidstest ikke over 300 ml blod. Den løbende ultralydsscanning af galdeblæren skal bestemme den hastighed, hvormed galdeblæren trækker sig sammen.

Når måltidstesten er overstået, vil forsøgspersonerne blive tilbudt et standardmåltid, som de instrueres i at indtage, indtil de føler sig behageligt mætte. I forbindelse med dette stilles igen en række spørgsmål, og størrelsen af det indtagne måltid noteres.

Tarmbakterierne fjernes med 3 lægemidler (antibiotika) (Vancomycin (500mg), Meropenem (500mg) og Gentamicin (40mg)), som opløses i 1 glas (200ml) frugtjuice og drikkes 1 gang dagligt i 4 på hinanden følgende dage. Første dosis indtages umiddelbart efter måltidstestens afslutning, mens anden, tredje og fjerde dosis udleveres (i alt 9 glas) til forsøgspersonerne på 1. forsøgsdag og skal indtages (udenfor laboratoriet) på hver af de følgende 3 dage ved aftenstid, ligeledes opløst i 200 ml juice.

Deltagerne opfordres til ikke at ændre deres fødeindtag i løbet af undersøgelsen. Under de 4 dages antibiotikakur vil forsøgsdeltagere være i daglig telefonisk kontakt med en læge.

Der opsamles afføring, blod, urin og spyt til bakterieanalyser lige før bakteriefjernelse, lige efter-, 8 dage efter-, 42 dage efter- og 180 dage efter bakteriefjernelse. Desuden anvendes afføringsprøverne til bestemmelse af lægemiddelkoncentration og en enkelt prøve fryses til brug ved eventuel udvikling af svær diarré (se herom senere). Spytprøve opsamles ved at stimulere forsøgspersonens spytdannelse med et stykke paraffin som tygges, og herefter lade forsøgspersonen spytte ud i et bæger over de næste 3 minutter. Urin, blod og spytprøve anvendes til bestemmelse af bakterieforekomst samt indhold af en række betændelsesmarkører og cellesignalstoffer.

Hos deltagere der giver særskilt samtykke vil der foruden ovenstående undersøgelser blive foretaget kikkertundersøgelse af tolvfingertarmen (gastroduodenoskopi) før og efter antibiotikakuren. I forbindelse med forsøgspersonens inklusion i studiet vil en læge informere om kikkertundersøgelsen, herunder bivirkninger og risici. Forsøgspersonen har derefter mulighed for at tilvælge kikkertundersøgelsen (i tillæg til det øvrige program). Hvis forsøgspersonen samtykker til kikkertundersøgelsen foretages den som anført på en selvstændig dag før antibiotikakuren (fra efter inklusion frem til dag -1) og på dag 3 (umiddelbart før sidste antibiotikaindtag).

Undersøgelsen udføres i Endoskopienheden på Gentofte Hospital efter 8 timers faste. Den udføres af trænet personale efter afdelingens standardinstruks, hvilket indebærer en kortvarig let bedøvelse med stoffet Propofol. Bedøvelsen kan sammenlignes med en kort slumretilstand, hvor åndedrættet er upåvirket. Undersøgelsen varer ca. cirka 15 minutter, men der påregnes yderligere 5 minutter til forudgående bedøvelse.

I forbindelse med undersøgelsen udtages 11 vævsprøver fra tolvfingertarmens slimhinde til genanalyser og der opsamles sekret til bestemmelse af bakterieforekomst i tolvfingertarmen. Efter undersøgelsen instrueres deltageren i, at han ikke må være fører af et motorkøretøj resten af dagen.

Skulle der indtræde moderate/svære bivirkninger i forbindelse med antibiotikakuren (specielt diarre eller anden almenpåvirkning), vil kikkertundersøgelsen dag 3 blive aflyst. Ligeledes vil kikkertundersøgelsen blive aflyst, hvis forsøgsdeltageren er utilpas eller alment påvirket.

## ETISKE OVERVEJELSER

Den væsentligste ulempe for den enkelte forsøgsdeltager er risikoen for at udvikle diarré i tilslutning til antibiotikakuren. I de 4 dage antibiotikakuren gives, vil forsøgspersonen være i daglig kontakt med den forsøgsansvarlige læge, og skulle der opstå diarré, vil lægen træffe beslutning om, hvorvidt forsøgspersonen skal ophøre med antibiotika kuren eller kan fortsætte uhindret i undersøgelsen. I tilfælde af svær, længerevarende diarré er der mulighed for, at forsøgspersonen kan behandles med en såkaldt "fæcestransplantation", dvs. kan få tilført sin egen bakteriekultur med henblik på hurtig normalisering tarmfunktionen og dermed ophør af diarreen. Dette gøres i almindelige kliniske sammenhænge ved langvarig diarré, der ikke umiddelbart lader sig behandle, og det udføres ved at man indfører den afføringsprøve, deltageren har afgivet ved undersøgelsens start, i endetarmen.

De 3 typer anvendte antibiotika optages kun i meget begrænset omfang over tarmens slimhinde, og kroppens optag af stofferne er således minimalt. Derfor er risikoen for bivirkninger (fraset diarré) også lille. For at opspore og undgå eventuelle medicinbivirkninger vil ethvert tilfælde af nyopståede symptomer under- eller umiddelbart efter antibiotikakuren blive meddelt den forsøgsansvarlige læge i forbindelse med den daglige kontakt. Der er i forbindelse med antibiotikakuren en lille risiko for, at forsøgspersonen udvikler allergi overfor et eller flere af de 3 anvendte antibiotika. Konsekvensen heraf kan være, at forsøgspersonen i fremtidige tilfælde af svære infektioner ikke kan modtage det pågældende antibiotika og da kan være henvist til et antibiotika med ringere effekt (under 0,1% risiko herfor). I tilfælde af allergisk reaktion vil forsøgspersonen blive henvist til videre allergiudredning og rådgivning hos specialafdeling for allergiske sygdomme.

Overfladisk venebetændelse er en teoretisk om end sjælden komplikation til anlæggelse af venekatetre. Tilstanden er ufarlig.

Kikkertundersøgelsen er for forsøgspersonen forbundet med minimalt ubehag, da undersøgelsen foretages i let bedøvelse med propofol. Teoretisk set vil der ved enhver kikkertundersøgelse med bedøvelse være risiko for medicinbivirkning, lungeproblemer (lungebetændelse og iltmangel), hjerterytmeforstyrrelse, instrument læsion og perforation af slimhinden, blødning. Når proceduren udføres ved et planlagt program og på raske unge mennesker, er risikoen for komplikationer ved undersøgelsen minimal. Undersøgelsen gennemføres kun såfremt forsøgsdeltageren føler sig veltilpas, og vil blive aflyst i tilfælde af moderate/svære antibiotikabivirkninger på dag 3.

Blodtabet ved deltagelse udgør maksimalt 300 ml pr testdag og er således samlet under 900 ml blod fordelt på 3 opsamlinger over 6 uger. Hos raske er dette ikke forbundet med risici i sig selv: udtagelsen kan dog føre til træthed umiddelbart efter undersøgelsen. Deltagernes blodprocent vil løbende blive kontrolleret og deltagerne vil evt., efter lægelig vurdering, blive tilbudt tilskud af jerntabletter for at undgå jernmangel.

Anvendelsen af ultralydsscanning samt måling af udåndingsluft er ikke forbundet med nogen form for ubehag eller risiko.

## FORMIDLING

Alle resultater af undersøgelsen formidles i form af artikler, som søges publiceret i internationale tidsskrifter.

## ØKONOMI

Projektet er initieret af 1. reservelæge, ph.d. Filip Krag Knop, Diabetologisk Forskningsenhed, Medicinsk afd. F, Gentofte Hospital. Hverken Filip Krag Knop eller den øvrige forskningsgruppe bag projektet har økonomiske interesser i udførelsen eller resultaterne af projektet.

Driftsudgifter til utensilier, screeningsblodprøver og bioanalytikerhjælp dækkes af Medicinsk afdeling F, Gentofte Hospital. Projektets øvrige driftsudgifter påtænkes dækket via private og offentlige fonde, der løbende vil blive ansøgt. Fondsmidlerne vil blive indsat på en fondskonto tilknyttet projektet under Medicinsk afdeling F, Gentofte Hospital, som er under hospitalets revision.

På nuværende tidspunkt har projektet modtaget støtte fra Medicinsk afdeling F, Gentofte Hospital i form af 2 måneders lægeløn til initiering af projektet. Herudover har projektet ikke modtaget finansiel støtte. Den forsøgsansvarlige har ikke økonomisk tilknytning til private virksomheder, fonde m.v., som har interesser i forskningsprojektet. Oplysninger om modtaget støtte (navn på støttegiver, støttebeløb herunder udbetalingsmåden) vil blive eftersendt til Videnskabsetisk Komité for Region Hovedstaden med henblik på etisk stillingtagen og godkendelse.

## VEDERLAG OG UDGIFTSGODTGØRELSE TIL FORSØGSDELTAGERE

Der ydes dækning af dokumenterede transportudgifter, som deltagere måtte have i forbindelse med deltagelse. Grundet projektets tidsmæssige omfang for den enkelte deltager ydes der ulempegodtgørelse på kr. 4000 beskattes som B-indkomst) til den enkelte deltager (kr. 5.000 til deltagere som samtykker til gastroduodenoskopi før og efter antibiotikakur). Ulempegodtgørelsen udbetales fra projektets fondskonto til deltagerens NEM-konto, når undersøgelsesforløbet er afsluttet for den enkelte deltager. Såfremt en forsøgsdeltager vælger at træde ud af forsøget, før dette er fuldført, vil ulempegodtgørelsens størrelse svare til aktuelle fremmødetid.

#

# BILAG 2, DELTAGERINFORMATION

**Eradikation af den humane tarmflora**

*Effekt på postprandial tarmhormonsekretion, glukosemetabolisme, knogleomsætning og tarmmikrobiom*

#### Forespørgsel om deltagelse i det biomedicinske forskningsprojekt

Vi er en forskningsgruppe bestående af forskere fra

- Diabetologisk Forskningsenhed, Medicinsk afdeling F, Gentofte Hospital,
- Novo Nordisk Foundation Center for Basic Metabolic Research, Københavns Universitet
- Mikrobiologisk afdeling, Rigshospitalet,
- Mavetarmkirurgisk afdeling, Herlev Hospital.

Vi arbejder med at undersøge sammenhængen mellem menneskets tarmbakterieflora og udviklingen af sukkersyge og knogleskørhed.

Vi vil spørge, om du vil være med i et videnskabeligt forsøg, der beskæftiger sig med denne sammenhæng.

På de næste sider beskrives nærmere, hvad forsøget går ud på, og hvordan det udføres. Det er frivilligt at deltage i forsøget, og du kan når som helst trække dig ud af forsøget - også selv om du har skrevet under på at ville deltage.

Tag dig god tid til at læse papirerne, før du beslutter dig. Du vil få mindst to dages betænkningstid, og du har ret til at medbringe et familiemedlem eller en anden bekendt, når du modtager nærmere information om forsøget mundtligt

MVH

Læge Kristian Hallundbæk Mikkelsen

(kristianmikkel@gmail, telefon61699759) (kontaktperson og forsøgsansvarlig)

1. reservelæge, ph.d. Filip Krag Knop (projektinitiator)

Post.doc, læge, ph.d. Morten Frost Nielsen

Diabetologisk Forskningsenhed, Gentofte Hospital

## BAGGRUND OG FORMÅL

Type 2-Diabetes (T2-DM, tidligere kaldet ”gammelmandssukkersyge”) og knogleskørhed, som også kaldes osteoporose, er hyppigt forekommende sygdomme forårsaget af blandt andet arv og livsstilsfaktorer. Indenfor de senere år er man blevet opmærksom på, at tarmens bakterier kan have betydning for udvikling af disse sygdomme. Det er blevet hævdet, at tarmbakterierne kan indvirke mere eller mindre gavnligt på kroppen afhængigt af sammensætningen af bakterierne.

Studier har vist, at den menneskelige tarmbakterieflora synes at hænge sammen med forekomsten af T2-DM; og ved at ændre sammensætningen af tarmbakterierne hos dyr har man kunnet påvirke dyrenes sukkeromsætning. Også ved knogleskørhed har man påvist, at en ændret tarmbakterieflora kan påvirke knogleomsætningen hos både dyr og mennesker. Det er kendt, at udskillelsen af en række hormoner, galdesyrer samt visse fedtsyrer påvirkes, når bakteriefloraen i tarmen ændres, men forklaringen bag disse effekter er aktuelt ukendt.

I dette projekt vil vi derfor undersøge, om en midlertidig fjernelse af tarmbakterierne hos mennesker medfører forandringer i sukker- og knogleomsætningen, ligesom vi vil forsøge at afklare, ad hvilke biokemiske veje disse forandringer i givet fald sker. Som led i dette vil vi undersøge, hvordan bakteriefjernelsen indvirker på:

1. udskillelsen af en række tarm-, og sukkerhormoner, foruden markører for opbygningen og nedbrydningen af knoglerne samt betændelse.

2. udskillelsen af galdesyrer fra galdeblæren.

3. appetitregulering og hvilestofskifte.

4. forekomsten af bakterier og betændelsesmarkører i urin og spyt.

Vi vil desuden undersøge sammensætningen af bakterierne, når disse er kommet tilbage til tarmsystemet.

Til dette formål ønsker vi at undersøge 12 raske mænd, af etnisk dansk oprindelse i alderen 18-40 år.

For at deltage som forsøgsperson er det et krav, at du:

- ikke har sukkersyge eller knoglesygdom,
- ikke har lever- eller nyresygdom
- ikke har lav blodprocent
- ikke har fået antibiotika (heller ikke malariaforebyggelse) indenfor de seneste 6 måneder
- ikke har mavetarmsygdomme eller har nære slægtninge med arvelige mavetarmsygdomme
- ikke er undervægtig (BMI <18.5 kg/m2) eller overvægtig (BMI >25 kg/m2)
- ikke er allergisk overfor de antibiotika (Vancomycin, Gentamycin eller Meropenem) vi skal anvende i forsøget eller er allergisk overfor såkaldte beta-lactamantibiotika (herunder almindelig penicillin)
- ikke er ryger og ikke spiser efter særlig diæt

## FORSØGETS OPBYGNING

Undersøgelsen strækker sig over 6 måneder og indebærer for dig som deltager i alt 6 besøg på afdelingen samt en 4 dages antibiotikakur i hjemmet. Hvis du - udover at samtykke til deltagelse i hovedforsøget - samtykker til at få foretaget kikkertundersøgelser af din tolvfingertarm, vil disse undersøgelser blive udført dels på en selvstændig dag før antibiotikakuren (fra efter inklusion frem til dag -1) og på dag 3 (umiddelbart før sidste antibiotikaindtag). For dig som deltager i supplerende kikkertundersøgelser er der således tale om 8 (og ikke 6) besøg på afdelingen.

Herunder er vist en oversigt over forsøget og beskrevet hvad der foregår på de enkelte dage. Forud for programmet vil du blive skriftligt og mundtligt informeret omkring forsøget og du skal give skriftligt samtykke til medvirken i forsøget.

-14 -1 0 1 2 3 4 8 42 180 dage

Screening

antibiotikakur

MT + OPS

MT + OPS

MT + OPS

OPS

OPS

MT = Måltidstest og ad libitum-måltid som beskrevet

OPS = Opsamling af afførings-, spyt- og urinprøver

Evt. gas = Udførelse af gastroduodenoskopi hos de deltagere som giver samtykke hertil

evt. gas

evt. gas

### Screening

Som potentiel forsøgsdeltager møder du i laboratoriet efter 10 timers faste. Her ses du af en læge; vægt, højde og blodtryk noteres og vi tager en række screeningsblodprøver (ca. 10 ml blod) og stiller en række spørgsmål med henblik på at sikre at de ovenforstående krav til forsøgsdeltageren er overholdt.

Såfremt du på basis af resultaterne fra screeningsmødet kan fortsætte i projektet, aftales datoer for undersøgelsesdagene. Hvis blodprøveresultaterne er unormale, rådgives du med hensyn til videre udredning.

### Måltidstest

På dagen for måltidstesten møder du i laboratoriet klokken 8 efter 10 timers faste. Inden forsøgsstart opsamles en urinprøve, og der anlægges en venekanyle (dropnål) i en vene (et tyndt blodkar) i hånden eller underarmen. Herfra tages ca. 40 ml blod til bestemmelse af en række genparametre, bakteriegener, betændelsesmarkører samt knogleskørhedsmarkører i blodet. Der foretages nu en måling af dit hvilestofskifte ved at opsamle din udåndingsluft i en særlig maske. Herefter bestemmes galdeblærens størrelse ved hjælp af en ultralydsscanning, og det egentlige forsøg startes: Du indtager 200 ml Nutridrink (ernæringsdrik med kakaosmag) samt 1,5 gram paracetamol (3 styk almindelige håndkøbssmertestillende tabletter) over ca. 10 minutter. Over de næste 4 timer opsamles løbende en række blodprøver via den anlagte venekanyle ligesom gasmåling på udåndingsluft og ultralydsscanning af galdeblæren gentages nogle gange. Desuden vil vi i løbet af de 4 timer stille dig en række spørgsmål vedrørende appetit og mæthedsfornemmelse.

Blodprøverne skal vise, om sammensætningen af markører for sukker- og knogleomsætningen ændres, ligesom der også måles på udskillelsen af en række tarmhormoner. I alt tages i forbindelse med måltidstest ikke over 300 ml blod. Den løbende ultralydsscanning af galdeblæren skal bestemme den hastighed hvormed galdeblæren trækker sig sammen.

Når måltidstesten er overstået, vil du blive tilbudt et standardmåltid, som du instrueres i at indtage, indtil du føler dig behageligt mæt. I forbindelse med dette stilles du igen en række spørgsmål, og størrelsen af det indtagne måltidet noteres.

### Antibiotikakur

Tarmbakterierne fjernes med 3 lægemidler (antibiotika) (Vancomycin (500mg), Meropenem (500mg) og Gentamicin (40mg)), som opløses i 1 glas (200ml) frugtjuice og drikkes 1 gang dagligt i 4 på hinanden følgende dage. Første dosis indtages umiddelbart efter måltidstestens afslutning, mens anden, tredje og fjerde dosis udleveres (i alt 9 glas) til dig på 1. forsøgsdag og skal indtages (udenfor laboratoriet) på hver af de følgende 3 dage ved aftenstid, ligeledes opløst i 200 ml juice.

Du opfordres til ikke at ændre dit fødeindtag i løbet af undersøgelsen.

Under de 4 dages antibiotikakur vil du dagligt blive telefonisk kontaktet af en læge.

### Opsamling af afførings-, blod-, urin- og spytprøver

Der opsamles afføring, blod, urin og spyt til bakterieanalyser lige før bakteriefjernelsen, lige efter-, 8 dage efter-, 42 dage efter- og 180 dage efter bakteriefjernelsen. Desuden anvendes afføringsprøverne til bestemmelse af lægemiddelkoncentration, og en enkelt prøve fryses til brug ved eventuel udvikling af svær diarré (se herom senere). Spytprøve opsamles ved at stimulere din spytdannelse med et stykke paraffin som tygges, og herefter lade dig spytte ud i et bæger over de næste 3 minutter. Urin og spytprøve anvendes til bestemmelse af bakterieforekomst samt indhold af en række betændelsesmarkører og cellesignalstoffer. Om morgenen før opsamlingen af spytprøven skal du undlade at børste tænder idet dette kan påvirke resultatet af bakteriebestemmelsen.

### Kikkertundersøgelse af tolvfingertarmen

Hos udvalgte forsøgsdeltagere vil der foruden ovenstående undersøgelser blive foretaget kikkertundersøgelse af tolvfingertarmen (gastroduodenoskopi) før og efter antibiotikakuren. I forbindelse med din inklusion i studiet vil en læge informere om kikkertundersøgelsen, herunder bivirkninger og risici. Herefter har du mulighed for at tilvælge kikkertundersøgelsen (i tillæg til det øvrige program). Hvis du samtykker til kikkertundersøgelsen foretages den som anført på en selvstændig dag før antibiotikakuren (fra efter inklusion frem til dag -1) og på dag 3 (umiddelbart før sidste antibiotikaindtag).

Undersøgelsen udføres i Endoskopienheden på Gentofte Hospital efter 8 timers faste. Den udføres af trænet personale efter afdelingens standardinstruks, hvilket indebærer en kortvarig let bedøvelse med stoffet Propofol. Bedøvelsen kan sammenlignes med en kort slumretilstand, hvor åndedrættet er upåvirket. Undersøgelsen varer ca. cirka 15 minutter, men der påregnes yderligere 5 minutter til forudgående bedøvelse.

I forbindelse med undersøgelsen udtages 11 vævsprøver fra tolvfingertarmens slimhinde til genanalyser, og der opsamles sekret til bestemmelse af bakterieforekomst i tolvfingertarmen. Efter undersøgelsen instrueres du i, at du ikke må være fører af et motorkøretøj resten af dagen.

Skulle der indtræde moderate/svære bivirkninger i forbindelse med antibiotikakuren (specielt diarre eller anden almenpåvirkning), vil kikkertundersøgelsen dag 3 blive aflyst. Ligeledes vil kikkertundersøgelsen blive aflyst, hvis du er utilpas eller alment påvirket.

## BIVIRKNINGER, RISICI OG ULEMPER FOR FORSØGSDELTAGERE

Den væsentligste ulempe for dig er risikoen for at udvikle diarré i tilslutning til antibiotikakuren. I de 4 dage antibiotikakuren gives, vil du være i daglig kontakt med den forsøgsansvarlige læge, og skulle der opstå diarré, vil lægen træffe beslutning om, hvorvidt du skal ophøre med antibiotika kuren eller kan fortsætte uhindret i undersøgelsen. I tilfælde af svær, længerevarende diarré er der mulighed for, at du kan blive behandlet med en såkaldt "fæcestransplantation", dvs. kan få tilført din egen bakteriekultur med henblik på hurtig normalisering tarmfunktionen og dermed ophør af diarreen. Dette gøres i almindelige kliniske sammenhænge ved langvarig diarré, der ikke umiddelbart lader sig behandle, og det udføres ved at man indfører den afføringsprøve, du har afgivet ved undersøgelsens start, i endetarmen.

De 3 typer anvendte antibiotika optages kun i meget begrænset omfang over tarmens slimhinde, og kroppens optag af stofferne er således minimalt. Derfor er risikoen for bivirkninger (fraset diarré) også lille. For at opspore og undgå eventuelle medicinbivirkninger vil ethvert tilfælde af nyopståede symptomer under- eller umiddelbart efter antibiotikakuren blive meddelt den forsøgsansvarlige læge i forbindelse med den daglige kontakt. Der er i forbindelse med antibiotikakuren en lille risiko for, at du udvikler allergi overfor et eller flere af de 3 anvendte antibiotika. Konsekvensen heraf kan være, at du i fremtidige tilfælde af svære infektioner ikke kan modtage det pågældende antibiotika og da kan være henvist til et antibiotika med ringere effekt (under 0,1% risiko herfor). I tilfælde af at du udvikler en allergisk reaktion, vil du blive henvist til videre allergiudredning og rådgivning hos specialafdeling for allergiske sygdomme.

Overfladisk venebetændelse er en teoretisk om end sjælden komplikation til anlæggelse af venekatetre. Tilstanden er ufarlig.

Kikkertundersøgelsen er for dig forbundet med minimalt ubehag, da undersøgelsen foretages i let bedøvelse med propofol. Teoretisk set vil der ved enhver kikkertundersøgelse med bedøvelse være risiko for medicinbivirkning, lungeproblemer (lungebetændelse og iltmangel), hjerterytmeforstyrrelse, instrument læsion og perforation af slimhinden, blødning. Når proceduren udføres ved et planlagt program og på raske unge mennesker, er risikoen for komplikationer ved undersøgelsen minimal.

Undersøgelsen gennemføres kun såfremt du føler dig veltilpas og vil blive aflyst i tilfælde af moderate/svære antibiotikabivirkninger på dag 3.

Blodtabet ved deltagelse udgør maksimalt 300 ml pr testdag og er således samlet under 900 ml blod fordelt på 3 opsamlinger over 6 uger. Hos raske er dette ikke forbundet med risici i sig selv: udtagelsen kan dog føre til træthed umiddelbart efter undersøgelsen. Din blodprocent vil løbende blive kontrolleret og du vil evt., efter lægelig vurdering, blive tilbudt tilskud af jerntabletter med henblik på hurtig genopretning af et blodtabet.

Anvendelsen af ultralydsscanning samt måling af udåndingsluft er ikke forbundet med nogen form for ubehag eller risiko.

## ANONYMISERING

Personidentificerbare data og prøver anonymiseres efter projektets afslutning. Ekstra biologisk materiale (blodprøver, spytprøver, afføringsprøver, vævsprøver og sekreter) vil blive opbevaret i op til 15 år efter forsøgets afslutning med henblik på gentagelse af eventuelle fejlanalyser og eventuelt behov for yderligere analyser. Efter 15 år destrueres disse prøver. Anvendelse af disse prøver til et nyt projekt vil kræve en fornyet godkendelse af De Videnskabsetiske Komitéer for Region Hovedstaden. Dele af det udtagne materiale (tarmsekret/afføring og spyt) vil blive sendt til udlandet til laboratorieanalyse, hvor materialet vil være omfattet af det pågældende lands lovgivning på området.

## FORDELE OG ULEMPER

Projektet vil ikke komme dig til gode (fraset den almindelige helbredsundersøgelse, som er en del af screeningsbesøget), men det vil belyse sammenhængen mellem tarmbakterieflora og sukkersyge og knogleskørhed, og derigennem muligvis bidrage til udviklingen af nye behandlingsmuligheder og forbedre de eksisterende for patienter med sukkersyge og knogleskørhed.

## DINE RETTIGHEDER SAMT UDTRÆDELSE OG AFBRYDELSE AF FORSØGET

Inden deltagelse i undersøgelsen bør du som potentiel deltager læse *Forsøgspersonens rettigheder i et biomedicinsk forskningsprojekt* og *Før du beslutter dig* (begge udgivet af Den Centrale Videnskabsetiske Komité og udleveret sammen med resten af informationsmaterialet). Heri er det beskrevet, at du som forsøgsperson uden yderligere begrundelser til en hver tid kan træde ud af undersøgelsen. Ekstraordinære omstændigheder, der umuliggør fuldførelse af forsøget for dig vil resultere i afbrydelse af det pågældende forsøgsforløb. Ligeledes vil ekstraordinære hændelser, der medfører, at projektet ikke lader sig fuldføre i sin helhed, føre til afbrydelse af forsøget for alle igangværende forsøgsdeltagere. Afbrydelse af forsøget vil afstedkomme en grundig information om årsagen hertil til alle involverede forsøgsdeltagere.

I tilfælde af utilsigtede hændelser dækker den almindelige patientforsikring. For rettigheder vedrørende aktindsigt, klageadgang og erstatning henvises til den vedlagte skrivelse *Forsøgspersoners rettigheder i et biomedicinsk forskningsprojekt* fra Den Centrale Videnskabsetiske Komité. Ønsker du information om resultatet af undersøgelserne, kan denne fås ved henvendelse til forsøgsansvarlige læge Kristian Hallundbæk Mikkelsen (se kontaktoplysninger på forsiden).

## GODKENDENDE MYNDIGHED OG ØKONOMI

Projektet er anmeldt til De Videnskabsetiske Komitéer for Region Hovedstaden og meldt til datatilsynet.

Projektet er initieret af 1. reservelæge, ph.d. Filip Krag Knop, Diabetologisk Forskningsenhed, Medicinsk afd. F, Gentofte Hospital. Hverken Filip Krag Knop eller den øvrige forskningsgruppe bag projektet har økonomiske interesser i udførelsen eller resultaterne af projektet. Projektet påtænkes finansieret via fondsmidler fra private og offentlige fonde uden økonomisk tilknytning til forskergruppen bag forskningsprojektet. Bevillingsgivere vil ikke få indflydelse på offentliggørelse af de opnåede resultater. Modtagne midler indsættes på en fondskonto tilknyttet til projektet. Kontoen administreres af Gentofte Hospital. Hverken medicinalindustrien eller øvrige private virksomheder er involveret i projektet.

Grundet projektets tidsmæssige omfang for dig yder vi ulempegodtgørelse på kr. 4000 (beskattes som B-indkomst) for din deltagelse (kr. 5.000 hvis du samtykker til gastroduodenoskopi før og efter antibiotikakur). Ulempegodtgørelsen udbetales fra projektets fondskonto til deltagerens NEM-konto, når undersøgelsesforløbet er afsluttet for den enkelte deltager. Såfremt du vælger at træde ud af forsøget før dette er fuldført, vil ulempegodtgørelsens størrelse svare til aktuelle fremmødetid.

## KONTAKTPERSON PÅ PROJEKTET

Projektets kontaktperson er læge Kristian Hallundbæk Mikkelsen, som også er klinisk ansvarlig læge. Ethvert spørgsmål vedrørende projektet eller deltagelse besvares med glæde på e-mail: kristianmikkel@gmail.com eller telefon: 61699759.

#

# BILAG 3, SAMTYKKEERKLÆRING, DELTAGELSE I FORSKNINGSPROJEKT

**Eradikation af den humane tarmflora**

*Effekt på postprandial tarmhormonsekretion, glukosemetabolisme, knogleomsætning og tarmmikrobiom*

Erklæring fra forsøgspersonen:

Jeg har læst den skriftlige information om forskningsprojektet og fået mundtlig information i et sprog, som jeg forstår. Jeg ved nok om formålet, metoderne, fordele og ulemper til at sige ja til at deltage. Jeg er informeret om, at det er frivilligt at deltage, og at jeg når som helst og uden begrundelse kan trække mit samtykke tilbage og udtræde af forsøget, uden at dette påvirker min ret til behandling eller andre rettigheder.

Jeg indvilger i at deltage i forskningsprojektet og har modtaget en kopi af dette samtykkeark samt en kopi af den skriftlige information til eget brug.

- Jeg ønsker oplysninger om egen helbredstilstand, der måtte fremkomme under gennemførelsen af projektet (sæt kryds):

Ja: ⁭ Nej:⁭

- Jeg ønsker information om de i projektet opnåede resultater, herunder evt. konsekvenser for mig (sæt kryds):

Ja:⁭ Nej:⁭

- Jeg giver hermed tilladelse til at materiale udtaget under forsøgets gennemførelse opbevares med henblik på senere analyser (sæt kryds):

Ja:⁭ Nej:⁭

Forsøgspersonens navn

Dato Underskrift

Erklæring fra den forsøgsansvarlige:

Jeg erklærer, at nedenstående forsøgsperson har modtaget mundtlig og skriftlig information om forskningsprojektet. Efter min bedste overbevisning er der givet tilstrækkelig information, herunder om fordele og ulemper, til at træffe et informeret valg.

Den forsøgsansvarliges navn

Dato Underskrift

# BILAG 4, SAMTYKKEERKLÆRING, GENNEMFØRELSE AF KIKKERTUNDERSØGELSER

**Eradikation af den humane tarmflora**

*Effekt på postprandial tarmhormonsekretion, glukosemetabolisme, knogleomsætning og tarmmikrobiom*

Erklæring fra forsøgspersonen:

Jeg har læst den skriftlige information om gastroduodenoskopi (kikkertundersøgelse af tolvfingertarm) og fået mundtlig information i et sprog, som jeg forstår. Jeg ved nok om formålet, metoderne, fordele og ulemper til at sige ja til at deltage. Jeg er informeret om, at det er frivilligt at deltage, og at jeg når som helst og uden begrundelse kan trække mit samtykke tilbage og udtræde af forsøget, uden at dette påvirker min ret til behandling eller andre rettigheder. Jeg er desuden bekendt med at mit samtykke til gennemførelse af gastroduodenoskopi ikke er en forudsætning for at kunne deltage i forskningsprojektet "Eradikation af den humane tarmflora. Effekt på postprandial tarmhormonsekretion, glukosemetabolisme, knogleomsætning og tarmmikrobiom".

Jeg indvilger i at deltage i gastroduodenoskopi før og efter antibiotikakur og har modtaget en kopi af dette samtykkeark samt en kopi af den skriftlige information til eget brug.

- Jeg ønsker oplysninger om egen helbredstilstand, der måtte fremkomme under gennemførelsen af kikkertundersøgelsen (sæt kryds):

Ja: ⁭ Nej:⁭

- Jeg giver hermed tilladelse til at materiale udtaget under kikkertundersøgelserne opbevares med henblik på senere analyser (sæt kryds):

Ja:⁭ Nej:⁭

Forsøgspersonens navn

Dato Underskrift

Erklæring fra den forsøgsansvarlige:

Jeg erklærer, at nedenstående forsøgsperson har modtaget mundtlig og skriftlig information om forskningsprojektet. Efter min bedste overbevisning er der givet tilstrækkelig information, herunder om fordele og ulemper, til at træffe et informeret valg.

Den forsøgsansvarliges navn

Dato Underskrift

# BILAG 5, ANNONCETEKST PÅ WWW.FORSOEGSPERSON.DK SAMT TEKST I OPSLAG PÅ GENTOFTE HOSPITAL

**Eradikation af den humane tarmflora**

*Effekt på postprandial tarmhormonsekretion, glukosemetabolisme, knogleomsætning og tarmmikrobiom*

**Deltagere til videnskabelig undersøgelse søges:**

Vi er en forskningsgruppe som undersøger sammenhængen mellem tarmens bakterieflora og udviklingen af sukkersyge og knogleskørhed.

Vi ønsker i et nyt forsøg at fjerne den normalt forekommende tarmflora for at se, hvordan dette påvirker omsætningen af sukker, knogle og en række hormoner og signalstoffer i kroppen.

Bakterierne vil blive fjernet ved hjælp af en 4 dages kur med antibiotika (antibakteriemedicin) som drikkes. Før og efter fjernelsen vil vi foretage en række undersøgelser; vi vil opsamle afførings, spyt og urinprøver og foretage en række blodprøver i forbindelse med et testmåltid. Dem der måtte give et særskilt samtykke til det, vil også få foretaget en kikkertundersøgelse af deres tolvfingertarm.

Til formålet ønsker vi at undersøge 12 raske mænd, af kaukasisk oprindelse i alderen 18-40 år.

For at kunne deltage som forsøgsperson er det et krav at du:

- ikke har sukkersyge eller knoglesygdom,
- ikke har lever- eller nyresygdom
- ikke har lav blodprocent
- ikke har fået antibiotika (heller ikke malariaforebyggelse) indenfor de seneste 6 måneder
- ikke har mavetarmsygdomme eller har nære slægtninge med arvelige mavetarmsygdomme
- ikke er undervægtig (BMI <18.5 kg/m2) eller overvægtig (BMI >25 kg/m2)
- ikke er allergisk overfor de antibiotika (Vancomycin, Gentamycin eller Meropenem) vi skal anvende i forsøget eller er allergisk overfor såkaldte beta-lactamantibiotika (herunder almindelig penicillin)
- ikke er ryger og ikke spiser efter særlig diæt

Forsøget forløber over et halvt år og indebærer i alt 6 besøg i vores laboratorium. 5 gange skal forsøgsdeltageren opsamle og aflevere afførings, urin og spytprøver og 3 gange vil vi udføre en måltidsundersøgelse. De 3 forsøgsdage med måltidsundersøgelsen varer ca. 6 timer mens de øvrige 3 besøg er af ganske kort varighed.

Vi tilbyder et vederlag for din tid i forbindelse med forsøgsdeltagelse.

Hvis du eventuelt kunne være interesseret i at deltage og opfylder ovenstående kriterier, er du velkommen til at kontakte den forsøgsansvarlige læge for mere uforpligtende information omkring projektet.

MVH

Kristian Hallundbæk Mikkelsen

Forsøgsansvarlig læge

Diabetologisk Forskningsenhed, Gentofte Hospital

mail: kristianmikkel@gmail.com

telefon: 61699759

1. reservelæge, ph.d. Filip Krag Knop

Post.doc, læge, ph.d. Morten Frost Nielsen

Diabetologisk Forskningsenhed, Gentofte Hospital

# BILAG 6, KOSTREGISTRERINGSSKEMA

**Eradikation af den humane tarmflora**

*Effekt på postprandial tarmhormonsekretion, glukosemetabolisme, knogleomsætning og tarmmikrobiom*

**Du bedes udfyld skemaet vedrørende dit fødeindtag vedrørende dit fødeindtag over de sidste 4 dage. Hvor mange gange har du spist følgende:**

En ring i hver linje

|  |  | 0 | 1-2  gange i løbet af de sidste 4 dage | 3-4  gange i løbet af de sidste 4 dage | 1  gang pr. dag | 2  eller flere gange pr. dag |
| --- | --- | --- | --- | --- | --- | --- |
| **a.** | **Plantemargarine** | 0 | 1 | 2 | 3 | 4 |
| **b.** | **Smør** | 0 | 1 | 2 | 3 | 4 |
| **c.** | **Ost** | 0 | 1 | 2 | 3 | 4 |
| **d.** | **Mælk** | 0 | 1 | 2 | 3 | 4 |
| **e.** | **Yoghurt, inkl. Actimel, cultura og A38** | 0 | 1 | 2 | 3 | 4 |
| **f.** | **Rugbrød, fuldkorn** | 0 | 1 | 2 | 3 | 4 |
| **g.** | **Rugbrød, uden kerner** | 0 | 1 | 2 | 3 | 4 |
| **h.** | **Havregrød** | 0 | 1 | 2 | 3 | 4 |
| **i.** | **Hvidt brød (normal)** | 0 | 1 | 2 | 3 | 4 |
| **j.** | **Hvidt brød (fuldkorn)** | 0 | 1 | 2 | 3 | 4 |
| **k.** | **Kartofler** | 0 | 1 | 2 | 3 | 4 |
| **l.** | **Grøntsager (kogte)** | 0 | 1 | 2 | 3 | 4 |
| **m.** | **Grøntsager (rå)** | 0 | 1 | 2 | 3 | 4 |
| **n.** | **Frisk frugt** | 0 | 1 | 2 | 3 | 4 |
| **o.** | **Juice** | 0 | 1 | 2 | 3 | 4 |
| **p.** | **Ris** | 0 | 1 | 2 | 3 | 4 |
| **q.** | **Spaghetti/pasta** | 0 | 1 | 2 | 3 | 4 |
| **r.** | **Leverpostej** | 0 | 1 | 2 | 3 | 4 |
| **s.** | **Kød** | 0 | 1 | 2 | 3 | 4 |
| **t.** | **Æg** | 0 | 1 | 2 | 3 | 4 |
| **u.** | **Fisk** | 0 | 1 | 2 | 3 | 4 |
| **v.** | **Pølse** | 0 | 1 | 2 | 3 | 4 |
| **x.** | **Kage** | 0 | 1 | 2 | 3 | 4 |
| **y.** | **Marmelade** | 0 | 1 | 2 | 3 | 4 |
| **z.** | **Sodavand, is** | 0 | 1 | 2 | 3 | 4 |
| **æ.** | **Chokolade, slik** | 0 | 1 | 2 | 3 | 4 |

**Har du ændret dine spisevaner i løbet af de sidste 4 dage**

Ja 1

Nej 2

Ved ikke 8

# BILAG 7, FORSØGSPERSONERS RETTIGHEDER I ET BIOMEDICINSK FORSKNINGSPROJEKT

**Eradikation af den humane tarmflora**

*Effekt på postprandial tarmhormonsekretion, glukosemetabolisme, knogleomsætning og tarmmikrobiom*

**DET VIDENSKABSETISKE KOMITÉSYSTEM**

**Forsøgspersonens rettigheder i et biomedicinsk forskningsprojekt.**

Som deltager i et biomedicinsk forskningsprojekt skal du vide at:

- din deltagelse i forskningsprojektet er helt frivillig og kan kun ske efter, at du har fået både skriftlig og mundtlig information om forskningsprojektet og underskrevet samtykkeerklæringen
- du til enhver tid mundtligt, skriftligt eller ved anden klar tilkendegivelse kan trække dit samtykke til deltagelse tilbage og udtræde af forskningsprojektet. Såfremt du trækker dit samtykke tilbage påvirker dette ikke din ret til nuværende eller fremtidig behandling eller andre rettigheder, som du måtte have
- du har ret til at tage et familiemedlem, en ven eller en bekendt med til informationssamtalen
- du har ret til betænkningstid, før du underskriver samtykkeerklæringen
- oplysninger om dine helbredsforhold, øvrige rent private forhold og andre fortrolige oplysninger om dig, som fremkommer i forbindelse med forskningsprojektet, er omfattet af tavshedspligt
- opbevaring af oplysninger om dig, herunder oplysninger i dine blodprøver og væv, sker efter reglerne i lov om behandling af personoplysninger og sundhedsloven
- der er mulighed for at få aktindsigt i forsøgsprotokoller efter offentlighedslovens bestemmelser. Det vil sige, at du kan få adgang til at se alle papirer vedrørende din deltagelse i forsøget, bortset fra de dele, som indeholder forretningshemmeligheder eller fortrolige oplysninger om andre
- der er mulighed for at klage og få erstatning efter reglerne i lov om klage og erstatningsadgang inden for sundhedsvæsenet
