# Supplementary material for: Effect of Antibiotics on Gut Microbiota, Gut Hormones and Glucose Metabolism
Source: PLoS One. 2015 Nov 12;10(11):e0142352. doi: 10.1371/journal.pone.0142352 (PMC4643023; doi:10.1371/journal.pone.0142352)
Supplement: S1 Table — No significant changes were observed in any of the above variables from before to immediately after or 42 days after the antibiotics course. Data shown as mean values (with 95% confidence intervals (CI)). (DOCX) [file pone.0142352.s005.docx]

**S1 Table. Mean values (with 95% confidence intervals (CI)) for resting metabolic rate, gastric emptying (time to peak in plasma paracetamol concentration), fasting and postprandial gallbladder volumes, composite appetite score and food intake during ad libitum meal.**

|  | **Day 0** | **95% CI** | **Day 4** | **95% CI** | **Day 42** | **95% CI** |
| --- | --- | --- | --- | --- | --- | --- |
| **Resting metabolic rate (kcal/day)** | **1985** | (1768-2202) | **1908** | (1626-2190) | **1875** | (1593-2157) |
| **Time to peak plasma paracetamol (min)** | **100** | (83-117) | **115** | (95-135) | **100** | (80-120) |
| **Gallbladder volume** |  |  |  |  |  |  |
| **Basal (cm^3^)** | **39** | (31-47) | **41** | (34-47) | **35** | (28-41) |
| **25 min postprandial (cm^3^)** | **13** | (7-20) | **14** | (7-21) | **13** | (6-20) |
| **55 min postprandial (cm^3^)** | **11** | (7-14) | **9** | (4-14) | **10** | (5-15) |
| **90 min postprandial (cm^3^)** | **12** | (7-17) | **9** | (4-14) | **10** | (5-15) |
| **235 min postprandial (cm^3^)** | **46** | (37-54) | **41** | (31-51) | **48** | (38-58) |
| **Composite appetite score (arb. units)** | **12479** | (12307-12652) | **12456** | (12287-12625) | **12315** | (12146-12485) |
| **Weight of ad libitum meal (g)** | **938** | (787-1088) | **926** | (779-1072) | **970** | (824-1116) |
